# Analyzing and interpreting spatial and temporal variability of the United States county population distributions using Taylor's law

**DOI:** 10.1371/journal.pone.0226096

**Published:** 2019-12-11

**Authors:** Meng Xu, Joel E. Cohen

**Affiliations:** 1 Department of Mathematics, Pace University, New York, New York, United States of America; 2 Laboratory of Populations, The Rockefeller University and Columbia University, New York, New York, United States of America; 3 Earth Institute and Department of Statistics, Columbia University, New York, New York, United States of America; 4 Department of Statistics, University of Chicago, Chicago, Illinois, United States of America; National Center for Global Health and Medicine, JAPAN

## Abstract

We study the spatial and temporal variation of the human population in the United States (US) counties from 1790 to 2010, using an ecological scaling pattern called Taylor's law (TL). TL states that the variance of population abundance is a power function of the mean population abundance. Despite extensive studies of TL for non-human populations, testing and interpreting TL using data on human populations are rare. Here we examine three types of TL that quantify the spatial and temporal variation of US county population abundance. Our results show that TL and its quadratic extension describe the mean-variance relationship of county population distribution well. The slope and statistics of TL reveal economic and demographic trends of the county populations. We propose TL as a useful statistical tool for analyzing human population variability. We suggest new ways of using TL to select and make population projections.

## Introduction

Spatial and temporal distributions of human populations hold important information about human societies and environments. City planning, business development, immigration policy, and many other activities rely on knowledge about the spatial and temporal distribution of people and their mobility [1–5]. Human population distributions in different areas are related. For example, metropolitan areas with high population density require large and sustained supplies of natural resources and products, which can affect the distribution of humans in neighboring areas. Human population distribution interacts with the environment and its resources, such as desertification [6] and biodiversity loss [7,8], and is affected by external conditions such as climate change and other environmental shifts [9–12]. Studying the spatial and temporal distribution of humans is a step towards describing and understanding such complex social and ecological interactions.

Human populations are not uniformly distributed in space or time, but depend on resources (e.g., food, jobs, and weather) and social development (e.g., urban and rural). Human population densities and numbers vary spatially and temporally. Specifically, the spatial unevenness or "centralization" of the population distribution is commonly characterized using two measures from economic theory: the Gini coefficient [13] and the Hoover index [14]. Both measures quantify the dissimilarity between the population count and inhabited area, and are intrinsically affected by the spatial scales used in the calculation. On the other hand, temporal variation of the population distribution is often measured by the coefficient of variation, which may vary with the mean population abundance. While these statistics have their own caveats, they are useful tools of describing the variation patterns to understand human behaviors (e.g., migration) and their impact on the environment.

Here we propose another measure to describe human population variation in space and time: Taylor's law (TL). TL is an ecological scaling pattern stating that the variance of species abundance (measured by number of individuals, i.e., "count," or by count per unit area, i.e., "density") is a power function of the mean species abundance. TL holds when the logarithm of the variance is well approximated as a linear function of the logarithm of the mean. The slope of the log(mean)-log(variance) relationship equals the exponent of the power function from the mean to the variance. For a given unit change in the log(mean) of population abundance, TL's slope equals the change in log(variance), which measures heterogeneity or scatter in the distribution of population abundance.

TL has been confirmed for numerous biological taxa and non-biological variables [15,16]. Bliss [17], Fracker and Brischle [18], Hayman and Lowe [19] and Taylor [20] proposed and showed empirically that a power law described well the variance of population count or population density as a function of the mean population count or population density in multiple samples. Taylor and colleagues [21–24] confirmed the empirical validity of TL using large-scale sample data of aphid, moth, and bird species across the United Kingdom. Most but not all empirical estimates of TL's slope fell between one and two (see Fig 2 in [25]). TL has also been verified for non-biological variables such as crime incidents [26], stock illiquidity [27], prime numbers [28], and tornado outbreaks [29]; in this last case, the slope exceeded four. Several models have been proposed to study the biological or statistical mechanisms of TL [30–32]. A unified explanation of TL is still lacking and probably does not exist, given the diversity of empirical situations where TL applies. Tests of TL against human population data are few [16,21,33–36]. Only Taylor et al. [21] (p. 392) and Taylor [16] (p. 392–394) analyzed the United States (US) population data. Other works tested TL using human population data from Norway, Italy and the whole world.

The slope of TL has three advantages for measuring the variability of sets of human population distributions. First, TL's empirical and theoretical roots in ecology can suggest possible mechanisms of human population distribution. Second, TL's slope measures the relative change in log(variance) with respect to a unit increase in log(mean) and, when TL is valid, the slope is independent of the average population abundance. This feature overcomes the density-dependence of other measures (e.g., coefficient of variation and the relative dispersion index from the mean-variance relationship of negative binomial distribution [37]) and makes TL ideal for comparisons of multiple populations across time or space. Third, TL can be tested under various spatial and temporal structures of population distribution. Such flexibility makes TL's slope a consistent measure for spatial and temporal variation.

Our goal here is to give a detailed empirical example, based on the history of the human population of the United States, to highlight for demographers and human ecologists the usefulness of TL as a statistical tool for analyzing human population variation. While previous studies have confirmed the usefulness of TL for human and nonhuman populations, we provide the first comprehensive analysis of different types of TL using US county populations, and we interpret the results from a demographic perspective.

We use TL to characterize the spatial and temporal variation of US county population count and population density reported in the decennial censuses from 1790 to 2010. We use counties as the basic spatial units. As the subordinate administrative divisions of the states, counties allow us to study the within-state population distribution and change. Although analyses of TL can be done similarly for populations from larger or smaller spatial scales (e.g., states and census tracts), they are not included in this paper due to the length limit. We test empirically three types of TL: spatial hierarchical TL, spatial TL and temporal TL. Then we focus on the interpretation and implication of statistics derived from these tests. We address the following specific questions: 1. What type of variation is quantified by each TL? 2. Is TL valid for all censuses and all states? If yes, what demographic information can be extracted from TL and what is the usefulness of such information? If not, what causes the exception(s)? 3. What are the similarities and differences in TL patterns between the US and other countries?

## Materials and methods

### Data

County data downloaded from NHGIS (National Historical Geographic Information System) Data Finder on 19 July 2018 [38] provide county population count (number of individuals residing in a county) in the decennial censuses from 1790 to 2010. County area (in square meters) and coordinates (latitude and longitude of the geographic centroid of each county) come from the 2000 TIGER/Line shapefile from 1790 to 2010. We join 23 shapefiles (one for each census), which specify each county's area, with the county population count data using the identifier "GISJOIN." GISJOIN is the GIS Join Match Code unique for each county that ever existed between 1790 and 2010. A "GISJOIN" code starts with letter "G" and is followed by two digital parts, the three-digit NHGIS Integrated State Code (STATENH) and the four-digit NHGIS Integrated County Code (COUNTYNH). For example, Autauga County of Alabama has GISJOIN code "G0100010," where "010" and "0010" are its state and county codes respectively. We denote states by their initials and territory by letter "T" (e.g., ND for North Dakota, NMT for New Mexico Territory and UTT for Utah Territory). Using these joined data, we define and calculate the population density (individuals km^-2^) of a county in a census as the ratio of county population count divided by the county area (in square kilometers, or km^2^). County population count and density are the two abundance measures used in this work.

The data include 50 states and 31 state-equivalents (29 historical territories, Puerto Rico (PR), and District of Columbia (DC)) and 4,128 counties and county-equivalents (e.g., parishes, boroughs, independent cities, and municipalities), historical or existing. We do not distinguish between a state and its equivalents, or between a county and its equivalents, and refer to them uniformly as state or county, respectively. On average, the number of counties per state is about 51 (≈ 4,128/81) during the entire census period (1790–2010). The largest county (in area) that ever existed is Northern District of AKT in the 1880 census (899,538.5 km^2^). The smallest county (in area) is Winchester City of VA in the 1900 census (0.28 km^2^). The most populous county is Los Angeles County of CA in the 2010 census (9,818,605 individuals). 72 combinations of counties and censuses report zero individuals. The densest county is New York County of NY in the 1920 census (38,279.08 individuals km^-2^). Only 62 combinations of counties and censuses have zero individuals per km^2^ because 10 of the 72 combinations with zero individuals lack area measurements. We do not correct the early censuses for their omission or misrepresentation of native American and slave populations.

### Three types of Taylor's law

Testing Taylor's law (TL) requires a hierarchical structure of the spatial and temporal population distribution. First, the mean and the variance of population abundance must be calculated at the lower spatial or temporal levels. Second, the calculated means and variances are grouped by the higher spatial or temporal levels to detect their relationship. US census data contain two nested spatial levels (state and county) and one temporal level (census), allowing us to test three types of TL.

If we calculate the spatial mean and spatial variance of county population abundance over counties within each state and each census, and find a linear relationship between the logarithmic mean and logarithmic variance across states within each census, it yields a spatial hierarchical TL. If we use the same set of means and variances and observe a linear relationship between their logarithms across censuses within each state, it corresponds to a spatial TL. Last, if we calculate the temporal mean and temporal variance of county population abundance over censuses within each county and each state, and find a linear relationship between their logarithms across counties within each state, it corresponds to a temporal TL.

Both the spatial hierarchical TL and the spatial TL examine the spatial distribution of population abundance of counties within each state, across states and across censuses respectively. Specifically, the spatial hierarchical TL analyzes the spatial heterogeneity (variation across states) of the spatial mean and the spatial variance of county population abundance. A greater slope of the spatial hierarchical TL means a greater degree of change between states in the spatial variance of county population abundance with respect to its spatial mean. This comparison is useful for the study of urbanization or "centralization" pattern in human geography [39].

The spatial TL analyzes the variation across censuses of the same means and variances. A greater slope of the spatial TL represents a greater degree of change between censuses in the spatial variance of county population abundance for a given unit of change in its spatial mean. This comparison can be used to study the temporal pattern of county population distribution for each state.

The temporal TL analyzes the variation across counties of the temporal mean and temporal variance of county population abundance. A greater slope in the temporal TL means a greater degree of change between counties in the temporal variance of county population abundance with respect to its temporal mean. This comparison can help us identify counties that experience high or low fluctuation in population abundance over time within a state.

Repeated testing of the same type of TL can extract further information about variation in the county population distribution. Specifically, repeated testing of the spatial hierarchical TL for each census allows us to detect a temporal trend of urbanization or counterurbanization. Repeated testing of spatial TL and temporal TL for each state provides a new way of classifying states according to their characteristic county population temporal dynamics.

### Statistical analysis of Taylor's law

We define the number of spatial or temporal units used in calculating a single mean-variance pair as the number of sampling units, denoted by *n*. We denote the number of pairs of finite positive mean and finite positive variance used in a single test of TL as *N*. In the main text we test each TL with *N* ≥ 5, with no constraint on the number *n* of sampling units used to calculate a mean-variance pair. In S1 File we repeat the analysis of all three types of TL when the number of sampling units for each mean-variance pair is at least 15 (*n* ≥ 15) and the number of pairs of finite positive mean and finite positive variance is at least five (*N* ≥ 5) (following the recommendation in [40]).

We test whether log(variance) is an approximately linear function of log(mean) using ordinary least-squares (ols) linear and quadratic regressions. Throughout log = log_10_. For each test of TL (for a unique combination of TL type and abundance measure), we first fit log(variance) as a linear function of log(mean) using the ols linear regression:

log(variance)=a+blog(mean)+ϵ, (1)

where the TL parameters *a* and *b* are respectively the intercept and slope of TL, and *ϵ* is the normal error with zero mean and constant variance. Following [21] (p. 388, their Eq 14), we also fit the same log(mean)-log(variance) pairs using the ols quadratic regression to examine the curvilinearity between log(mean) and log(variance):

$$\log\left(variance\right)=c+d\;\log\left(mean\right)+e{\left[\log\left(mean\right)\right]}^{2}+\epsilon .$$ (2)

Here *e* is the quadratic coefficient. If *e* > 0, the nonlinearity between log(mean) and log(variance) is convex. If *e* < 0, the nonlinearity is concave. We call the mean-variance relationship described by Eq 2 the quadratic TL (QTL). If *b* of Eq 1 is significantly nonzero and *e* of Eq 2 is not significantly different from zero, then we do not reject the hypothesis that TL is an appropriate model for the mean-variance relationship. If *e* of Eq 2 is significantly different from zero, then we prefer QTL over TL as a description of the mean-variance relationship. Neither TL nor QTL is suitable for describing the relationship when the linear and quadratic regressions are not significant. Here "significance" means that the p-value of the corresponding parameter or regression is less than 0.05 or, after Bonferroni correction, the p-value is less than 0.05/number of tests. We obtain the point estimate, standard error and p-value of each coefficient parameter of TL (*a* and *b*) and QTL (*c*, *d* and *e*), and the p-value of the overall regression of QTL. We also record the adjusted coefficient of determination (adj. *R*^2^) for each ols regression. We perform regression diagnostics for the linear and quadratic models and present the results in the S1 File.

For each type of TL, we use one minus adj. *R*^2^ (1-adj. *R*^2^) of the best-fitting model (TL or QTL) to measure the dissimilarity of county distribution among spatial and temporal units relative to the standard of TL or QTL. We may think of 1-adj. *R*^2^ as one measure of the degree to which a log(mean)-log(variance) pair deviates from the linear or quadratic function (Eqs 1 and 2). Higher values of 1-adj. *R*^2^ correspond to larger deviations, indicating a greater degree of dissimilarity. Specifically, for the spatial hierarchical (Q)TL and spatial (Q)TL, 1-adj. *R*^2^ reflects the dissimilarity (from the (Q)TL standard) in the spatial distribution of county population abundance among states within each census and among censuses within each state, respectively. For the temporal (Q)TL, 1-adj. *R*^2^ measures the dissimilarity (from the (Q)TL standard) in the temporal distribution of county population abundance among counties within each state.

All figures and statistical analyses, including summary statistics and ordinary least-squares regressions, are done in R [41]. The data that support the findings of this study are openly available in IPUMS National Historical Geographic Information System: Version 13.0 [Database] at http://doi.org/10.18128/D050.V13.0.

## Results

Results when county population count is used as the abundance measure are in the main text. Results regarding county population density are in the S1 File.

### Empirical confirmation of TL

**Fig 1:**
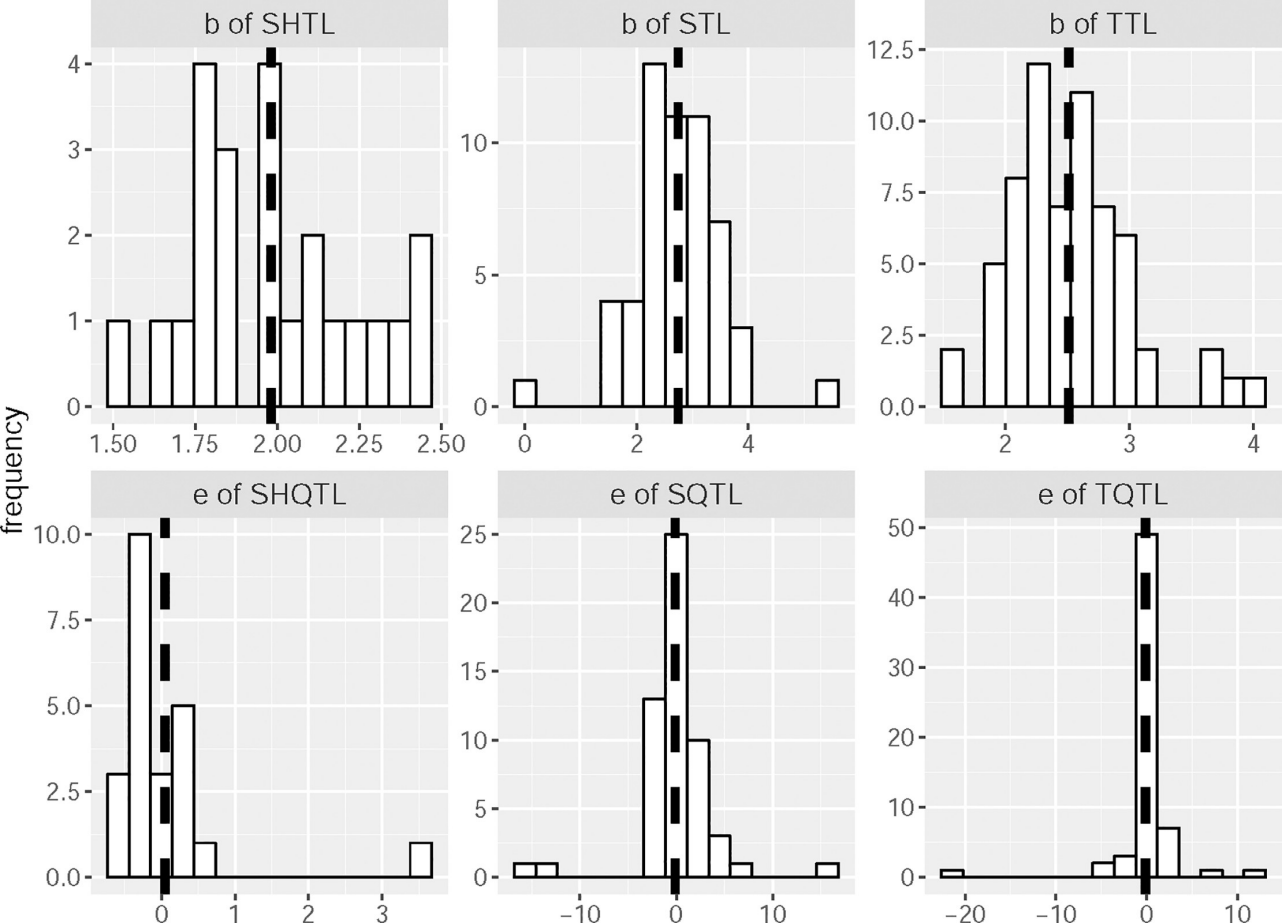
Point estimate of regression coefficient of Taylor's law (TL) and quadratic Taylor's law (QTL). *b* is the slope estimate of the spatial hierarchical TL (SHTL), spatial TL (STL) or temporal TL (TTL). *e* is the quadratic coefficient of the spatial hierarchical quadratic TL (SHQTL), spatial quadratic TL (SQTL) or temporal quadratic TL (TQTL). Dark dashed line shows the average value for the corresponding estimate.

Visually, the spatial hierarchical TL describes well the relationship between mean and variance of county population count (S1 Fig). Spatial TL, temporal TL and their quadratic forms are adequate models for the corresponding mean-variance relationship in a majority of states (S2 and S3 Figs). In only a few states neither the linear nor the quadratic model depicts the mean-variance relationship. Regression analysis confirms these observations (Table 1). The quadratic model (Eq 2) is favored over the linear model (Eq 1) in more than half of the states for the spatial TL, and in one quarter of the states for the temporal TL. The linear model is preferred in more states after Bonferroni correction of regression significance. Variability in the slope of spatial hierarchical TL is smaller than that of the spatial and temporal TL (Fig 1). Quadratic coefficients of all three types of QTL are close to zero on average, with average values 0.045 for the spatial hierarchical QTL, -0.12 for the spatial QTL, and -0.14 for the temporal QTL, but there are extreme outliers for each form of QTL.

**Table 1:** Summary of the regression statistics of the Taylor's law (TL) (Eq 1) and quadratic Taylor's law (QTL) (Eq 2) models using county population count.

measure	model	number of tests	average				proportion of p-value < 0.05		proportion of p-value < 0.05/number of tests	
			*n*	*N*	adj. *R*^2^ of Eq 1	adj. *R*^2^ of Eq 2	*b* in Eq 1	*e* in Eq 2	*b* in Eq 1	*e* in Eq 2
count	spatial hierarchical (Q)TL	23	51	42	0.76	0.76	22/23 (96%)	0/23 (0%)	22/23 (96%)	0/23 (0%)
	spatial (Q)TL	55	53	17	0.88	0.90	53/55 (96%)	30/55 (55%)	49/55 (89%)	17/55 (29%)
	temporal (Q)TL	64	12	53	0.80	0.81	60/64 (94%)	16/64 (25%)	53/64 (83%)	7/64 (11%)

*b* denotes the slope estimate of TL and *e* denotes the quadratic coefficient estimate of QTL. *n* represents the number of spatial or temporal units used to calculate a mean-variance pair. *N* represents the number of mean-variance pairs in a single testing of TL or QTL. The proportion of significantly nonzero regression coefficients was calculated without (significance level equals 0.05) and with (significance level equals 0.05/number of tests) Bonferroni correction separately.

Specifically, regardless of Bonferroni correction, spatial hierarchical TL (Eq 1) is not rejected in favor of its quadratic form (Eq 2) in all but one of the 23 censuses (S1 and S2 Tables). In the 1790 census, neither the linear nor the quadratic model is superior to the constant model (S1 and S2 Tables), indicating no consistent relationship between the means and the variances of county population across states during the early settlement of US.

Spatial TL and QTL are tested in 55 states (S3 and S4 Tables). Without Bonferroni correction, in two of the 55 states (AKT and NMT), neither the linear nor the quadratic model describes the mean-variance relationship well (regression p-value>0.05). In the other 53 states, spatial TL is not rejected in 23 states and spatial QTL is not rejected in 30 states. With Bonferroni correction, neither the spatial TL nor the spatial QTL describes the mean-variance relationship well in five states (AKT, AZT, NMT, ND and UTT). In the other 50 states, spatial TL is not rejected in 32 states and spatial QTL is not rejected in 18 states.

Temporal TL and QTL are tested in 64 states (S5 and S6 Tables). Without Bonferroni correction, neither the temporal TL nor the temporal QTL describes adequately the mean-variance relationship in four states (AKT, ART, FLT and WYT). In the remaining 60 states, temporal TL is not rejected in 44 states and temporal QTL is not rejected in 16 states. With Bonferroni correction, neither the temporal TL nor the temporal QTL describes adequately the mean-variance relationship in 11 states (AKT, AZT, ART, DC, FLT, IDT, MIT, MTT, NMT, RI and WYT). In the remaining 53 states, temporal TL is not rejected in 46 states and temporal QTL is not rejected in seven states.

For the spatial hierarchical TL, the mean-variance pairs are scattered in the 1790 census (S1 Fig). In particular, in DE (lowest point) and VA (highest point) we see the largest deviations from the fitted regression lines. The scatter disappears in the subsequent censuses. The violation of spatial TL or QTL of county population count in AKT and NMT is due to their drastic territorial changes preceding their transition to statehood (S2 Fig, S3 and S4 Tables). Among all the 50 current states, ND shows a unique mean-variance relationship: in most censuses the average county population count changes slightly while the variance continues to rise. The reason is that, during most of the 20th century (after the large increase in population count during the earlier censuses due to the farmers migrating into ND), ND people moved from agricultural lands to large cities, especially young adults emigrating from rural areas [42]. This internal migration caused an uneven population distribution among ND counties. The temporal TL and QTL are rejected in states with either large territorial changes (AKT) or short census periods (two censuses available in ART, FLT and WYT) (S3 Fig, S5 and S6 Tables).

### Population growth trend and pattern illustrated by TL and QTL

**Fig 2:**
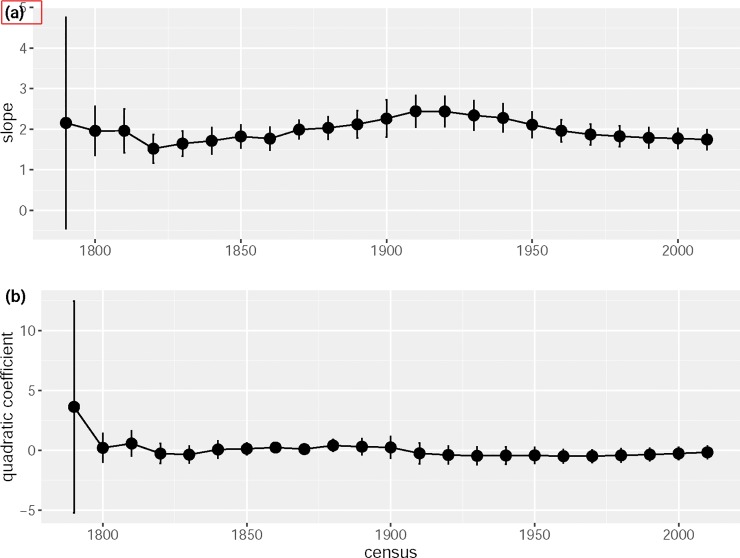
**Time series of (a) the slope estimate of spatial hierarchical TL and of (b) the quadratic coefficient estimate of the spatial hierarchical quadratic TL.** The solid circle represents the point estimate and the vertical bar shows the corresponding 95% confidence interval.

TL and QTL of all three types reveal useful information about the population dynamics of US counties. First, the slope of the spatial hierarchical TL fluctuates before the 20th century and reaches a record high in 1910 (Fig 2), reflecting the early boundary changes and the population centralization caused by urbanization in the 19th century. Since the 1910 census, the slope has been declining. This decline occurred because suburbanization (or counterurbanization) or the emergence of new metropolitan areas across US counties during the 20th century led to increasing spatial evenness in county population count as a function of increasing mean county population count. Compared to other types of TL, the spatial hierarchical TL shows smallest variability in its slope, ranging from 1.5 to 2.5. If the trend in slope over the past five decades continues, the slope will likely fall in the 2020 census.

**Fig 3:**
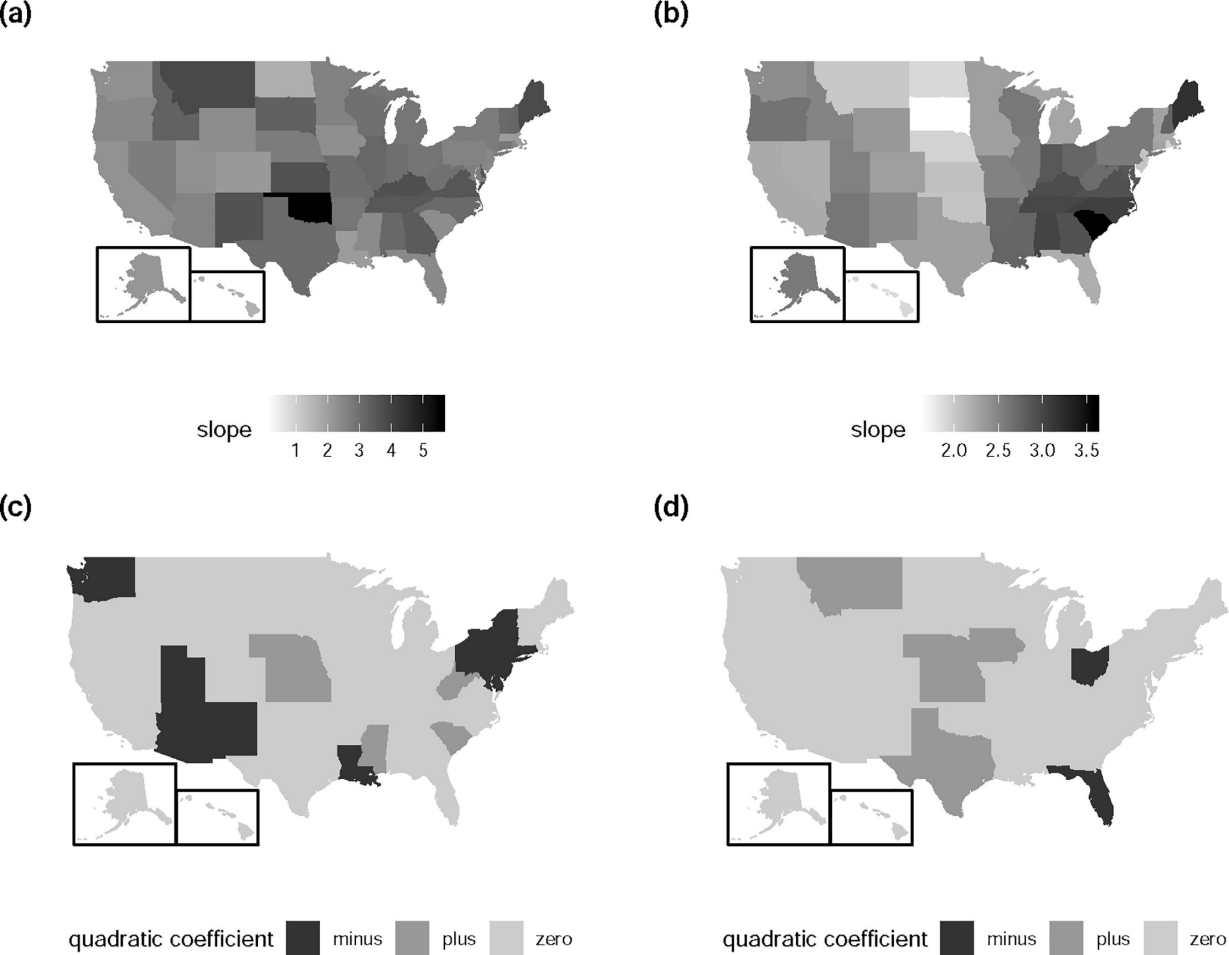
**US maps showing the slope (a and b) and the sign of quadratic coefficient (c and d) of the spatial (Q)TL (a and c) and temporal (Q)TL (b and d).** Sign of the quadratic coefficient is determined by the coefficient significance after Bonferroni correction. Specifically, "zero" is defined when the p-value of the quadratic coefficient (*e* of Eq 2) is greater than 0.05/number of tests (55 for spatial (Q)TL and 64 for temporal (Q)TL), meaning that the coefficient is not significantly different from zero. "minus" is defined when the p-value is smaller than 0.05/number of tests and the point estimate of *e* is less than zero. "plus" is defined when the p-value is smaller than 0.05/number of tests and the point estimate of *e* is larger than zero. This US map is made with the package fiftystater [43] in R. fiftystater is free software that can be redistributed and/or modified under the terms of the GNU General Public License as published by the Free Software Foundation, version 3.

The spatial TL exhibits great differences among states. With Bonferroni correction, TL is the superior model in 31 current states. The slope of TL is not different from two in AK, AR, CO, HI, IA, MA, MT, OK, OR and WY, and is significantly greater than two in AL, CA, FL, GA, ID, IL, IN, KY, ME, MI, MN, MO, NV, NH, NC, OH, TN, TX, VT, VI and WI (Fig 3A). The higher slopes reflect the greater degree of centralization in county population distribution.

With Bonferroni correction, the spatial QTL depicts the mean-variance relationship better than TL in 18 current states. With the exception of SD, the quadratic coefficient (*e* in Eq 2) is significantly smaller than zero in AZ, CT, DE, LA, MD, NJ, NM, NY, PA, RI, UT and WA, and greater than zero in KS, MS, NE, SC and WV (Fig 3C). The former group contains states with declining centralization over time (i.e., log(variance) grows slower than linearly with log(mean)), probably due to counterurbanization during the past century. The latter group includes states with increasing centralization over time (i.e., log(variance) grows faster than linearly with log(mean)), suggesting rapid metropolitan development in these states. The mean-variance relationship in some states may be better fitted by a cubic function than by a linear or quadratic function (e.g., AR, IN and MS in S7 Fig).

Temporal TL and QTL reveal differences in the temporal variability of population count among counties within a state. Since most states have counties with small population change over the censuses, counties with great population fluctuation will dictate the slope of temporal TL. With Bonferroni correction, the temporal TL is the better model compared to the temporal QTL in 41 current states. The slope is greater than two in AL, AR, CA, CO, GA, ID, IL, IN, KY, LA, MI, MO, NY, NC, OR, PA, SC, TN, UT, VI, WV and WI, and not different from two in AK, AZ, CT, HI, ME, MD, MA, MN, MS, NV, NH, NJ, NM, ND, OK, SD, VT, WA and WY (Fig 3B). A higher slope implies that some counties experience greater population change. With Bonferroni correction, the temporal QTL is the better model compared to the temporal TL in seven current states. The quadratic coefficient is smaller than zero in FL and OH, and greater than zero in IA, KS, MT, NE and TX (Fig 3D).

### Spatial and temporal dissimilarity of county population distribution

According to the spatial hierarchical TL, except the 1790 census, the dissimilarity (deviation from TL) of county spatial population distribution among states is highest in the 1900 census (1-adj. *R*^2^ = 0.34), followed by the 1810 and 1800 censuses (with 1-adj. *R*^2^ equaling 0.29 and 0.28 respectively) (S1 and S2 Tables). This observation may be attributed to large wave of international immigration to the US at the beginning of the 20th century and the geographic change during the earlier census periods. The 1870 census corresponds to the smallest across-state dissimilarity (deviation from TL) among all censuses with 1-adj. *R*^2^ of 0.13. The dissimilarity in the most recent 2010 census ranks 12th among all 23 censuses.

The spatial TL (or QTL) points to IA, OK and MT as the three states with the largest dissimilarity (deviation from TL or QTL) of county spatial population distribution among censuses (with 1-adj. *R*^2^ equaling 0.26, 0.18 and 0.15 respectively) (S3 and S4 Tables), which may be explained by various economic and political events in their state history. First, IA experienced two distinct periods of population change. From 1850 to 1900, farming and coal industries brought many out-of-state immigrants to IA who contributed to rapid population growth [44]. From 1910 to 2010, IA experienced gradual change in its economic sectors, mainly from agriculture to manufacturing operations, which led to small population growth and population redistribution from rural to urban areas due to within-state migration. During 1930s-1950s, OK's population declined (decreasing mean) and lost farming populations to urban areas (increasing variance) due to the Dust Bowl [45]. In MT, the Enlarged Homestead Act in 1909 (a law passed by the US to promote dryland cultivation in MT) seems to have contributed to the spatial evenness of county population distribution, as reflected by the drop-off in variance in the 1910 census. In the other 50 states where spatial TL or QTL describes the data well (without Bonferroni correction), 45 states have a 1-adj. *R*^2^ lower than 0.1.

Using the temporal TL (or QTL), the three states with the largest dissimilarities (deviation from TL or QTL) are MS, SD and OK, with 1-adj. *R*^2^ equaling 0.35, 0.31 and 0.29 respectively (S5 and S6 Tables). In MS, the relatively small change in population of several counties (e.g., Jefferson Davis County, Hinds County and Benton County) increases the overall dissimilarity of temporal distribution of county population among counties. In SD, the presence of historically short-lived counties (e.g., Sterling County and Schnasse County) and two counties (Minnehaha County and Pennington County) with fast-growing cities (Sioux Falls and Rapid City) contribute to the across-county dissimilarity. In OK, the dissimilarity is driven by Oklahoma County and Tulsa County, sites of the state's two most populous cities (Oklahoma City and Tulsa). HI, CA and OR have the least dissimilarity among current states, with 1-adj. *R*^2^ equaling 0.01, 0.02 and 0.04 respectively.

## Discussion

### Comparison of TL patterns in the US and other countries

To the authors' knowledge, all prior works on TL for human population abundance examined the spatial hierarchical or spatial (Q)TL. In addition, most studies focused on testing the spatial TL at the national level, instead of at the subnational level as done in the current work. (The exceptions in [16,33] are described below.) Nevertheless, the comparison of spatial TL across countries provides useful insights about changes in population distributions.

Taylor et al. [21] were the first to test a spatial TL against human population census data. Despite omitting details about the data and fitting method, they observed that the spatial TL described the relationship of mean and variance of male population abundance across multiple censuses, with a slope significantly greater than two (2.04±0.01). This result probably describes variation among states, not counties, and is consistent with our finding: at the county level within states, in most states the spatial TL slope was greater than two (Fig 1). Independently, using the US county-level data, Taylor [16] examined the spatial hierarchical TL (which he called "ensemble" TL) and observed the same temporal trend of its slope as in our Fig 2A (see Fig 11.6 in [16]).

Two independent works studied the spatial TL using human population density outside the US. Cohen et al. [34] tested a spatial TL for the mean and variance of Norwegian county (equivalent to the US state) population densities across 33 years. They also repeated the same analysis using Norwegian region (equivalent to US region or division) and municipality (equivalent to the US county) population densities. They found that the spatial TL pattern depends on the temporal scale and reflects the population change caused by economic development. This finding is consistent with our findings that the spatial TL can reveal different development stages of county populations within a state (see **Demographic insights from observed TL patterns** below).

Using the methods of Cohen et al. [34], Benassi and Naccarato [33] tested a spatial TL for the mean and variance of Italian regional (equivalent to US divisional) population densities across 41 years. Both studies used three different weighting methods (equally weighted, areally weighted and population weighted) to calculate the set of spatial means and spatial variances. Since the areally weighted means and variances of population density are equivalent to the equally weighted means and variances of population count ($\sum density_{i}\cdot \frac{area_{i}}{area}=\sum \frac{count_{i}}{area_{i}}\cdot \frac{area_{i}}{area}=\frac{\sum count_{i}}{area}$), we compare our results to the spatial TL of population density under areal weighting [33,34]. The spatial TL slope is greater than two for Norwegian county (or region or municipality) population densities and less than two for Italian region population densities. These cross-country and cross-state comparisons suggest that the spatial TL slopes reflect the different patterns of change in the spatial population distribution.

**Fig 4:**
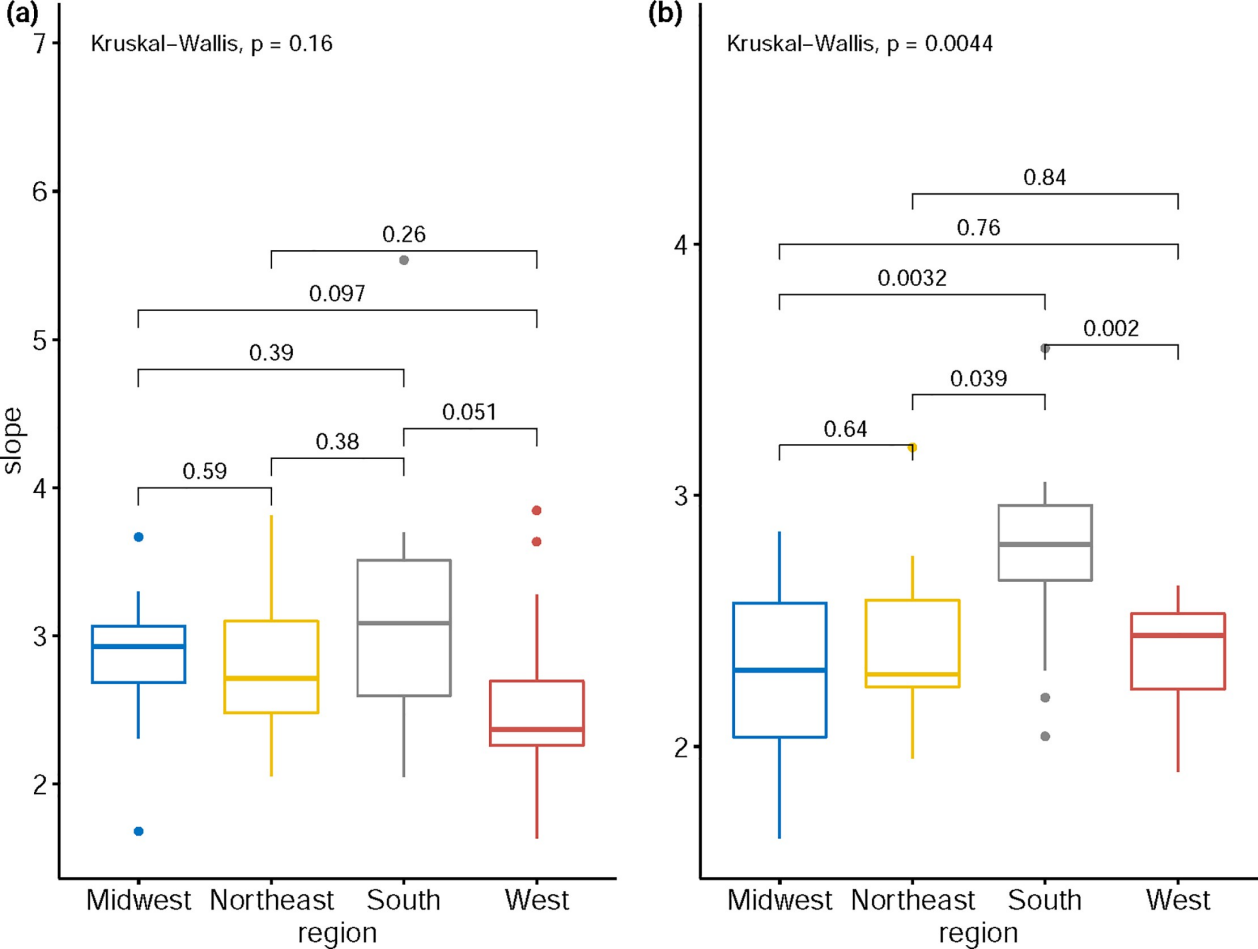
**Comparison of the slope of spatial TL (a) and temporal TL (b) across four regions (Midwest, Northeast, South and West) of the US.** The overall comparison is tested by the Kruskal-Wallis test and each pairwise comparison is done by the Wilcoxon rank sum test. The number above each pair shows the corresponding p-value. The three horizonal bars in each box plot, from top to bottom, represent the third quartile (Q3), median (Q2), and first quartile (Q1) respectively. The upper and lower whiskers are defined respectively as min(max(slope), Q3+1.5*(Q3-Q1)) and max(min(slope), Q1-1.5*(Q3-Q1)). Outliers (denoted by dots) denote slope values that are greater than Q3+1.5*(Q3-Q1) or smaller than Q1-1.5*(Q3-Q1).

Benassi and Naccarato [33] also studied the spatial TL subnationally for each of the three Italian macro-geographical areas (North, Centre, South and Island, equivalent to US regions). A spatial mean and a spatial variance of population density were calculated across the provinces (equivalent to US states) within each area. Using the seemingly unrelated regression method, they found that the spatial TL slope was smaller in the North area than in the South area. The authors attributed this observation to the steady influx of international immigration to the North, the less-developed economic condition of the South, and the internal migration from South to North. Comparing the state-level slopes of spatial and temporal TL among US regions shows that the South has the highest median slope (Fig 4). These observations reflect the similarity of regional characteristics in economic development and migration between the US and Italy [46–48]. They also indicate the usefulness of spatial TL in reflecting economic and demographic patterns.

Our analysis shows that the spatial TL is not the only relation between the mean and variance of spatial population distribution of US counties. The spatial QTL in multiple states reflects a different evolution of spatial population distribution. Other works that tested spatial TL using the Norwegian [34] and world [35] population density have also documented curvature in the mean-variance relationship.

### Demographic insights from observed TL patterns

TL quantifies the variability of the spatial or temporal distribution of county population abundance in relation to the mean county population abundance. We show that the observed TL pattern and slope can identify economic, demographic, and political events. The insights derived from TL thus can help government and policy makers to plan economic and demographic development at local or regional levels. To illustrate this, we summarize and distill the main points from our results.

The temporal trend in the slope of the spatial hierarchical TL classifies the US county population distribution dynamics into two periods. During the 19th century, the US frequently acquired land and established states, causing fluctuation in the slope. In addition, the industrial revolution propelled population urbanization, leading to an overall increase in the slope starting 1820. The territorial boundaries of the contiguous US became stable after the state of Arizona was added as the 48th state in 1912 (before Alaska and Hawaii in 1959). During the 20th century, suburbanization and counterurbanization were the dominating factors that shaped population distribution [49–53]. In recent decades, the formation of megalopolises enhanced the connectivity between urban areas [54,55]. These factors together contribute to the decrease of the spatial hierarchical TL slopes. The spatial hierarchical TL does not indicate a "clean break" of US population distribution around 1970 compared to previous decades [56,57].

**Fig 5:**
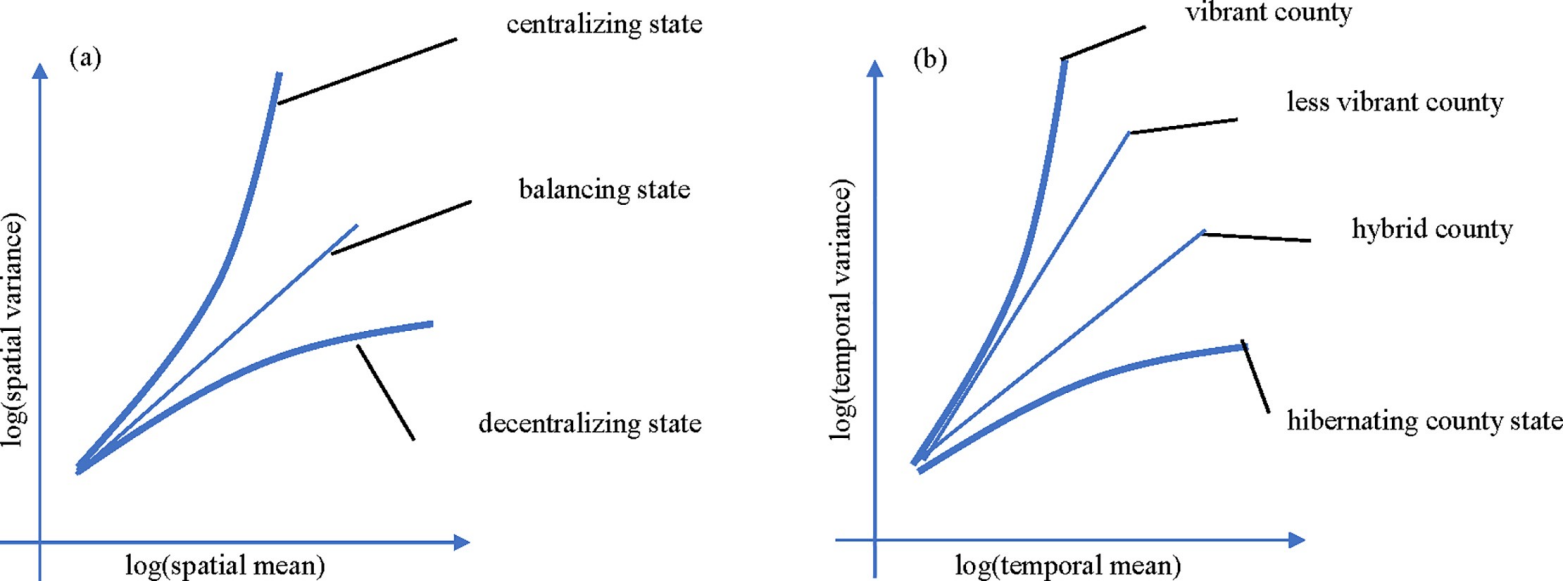
Temporal mean-temporal variance relationships on log-log scale. These illustrative examples are selected from a continuum of variation; they do not represent distinct categories. (a) States showing sublinear (superlinear) increase in log(spatial variance) as a function of log(spatial mean) are defined as decentralizing (centralizing) states, compared to the balancing states where log(spatial variance) grows linearly with log(spatial mean). (b) States are classified by the temporal fluctuation of county population count of their most populous (on average) counties: hibernating county (county with the least temporal fluctuation), hybrid county (county with intermediate temporal fluctuation), less vibrant county (county with large temporal fluctuation) and vibrant county (county with the largest temporal fluctuation).

The spatial TL and spatial QTL reveal a continuum of patterns in the log(mean)-log(variance) relationship of county population distribution: convexity (or superlinearity), linearity, and concavity (or sublinearity) (Fig 5A). States showing a convex relationship are likely in their early developmental stage, experiencing fast growth in their metropolitan areas. States showing a concave relationship have probably passed the early developmental stage and entered a period when suburbanization or counterurbanization leads to a more evenly distributed county population. The cubic pattern observed in some of the early states corroborates this claim. States with a linear relationship are probably in the stage where both centralization and decentralization are occurring in multiple areas simultaneously within the state. These illustrative examples are selected from a continuum of variation; they do not represent distinct categories.

The temporal TL and temporal QTL reflect the spatial heterogeneity of counties' temporal population distribution, since the linearity or curvature of temporal variances as a function of temporal means is largely determined by the most populous county in a state (Fig 5B). When the most populous county exhibits higher temporal fluctuation in population abundance (vibrant county), the mean-variance relationship is convex. A county with large time-averaged population that experiences small population change over time (a hibernating county) will produce a concave relationship. The linearity in the relationship could describe counties with intermediate population change over time, in either less vibrant county (TL slope > 2) or hybrid county (TL slope = 2).

What demographic factors determine the values of the TL slope? Our analysis here does not provide a direct answer. Cohen et al. [34] used a stochastic multiplicative population growth model to predict the slope of a spatial hierarchical TL at large time. They found that the size of the slope is analytically related to the population growth pattern: slope > 2 for supercritical population (population abundance increases on average) and slope < 2 for subcritical population (population abundance decreases on average). Our current work shows that the slope of the spatial hierarchical TL is less than two in recent censuses, implying that the average county population is losing population under the assumption of multiplicative growth. This claim is contradicted by the finding that population gain is still the main trend for a majority of US counties in the past century [58]. A plausible explanation for such a discrepancy is that the observed TL slope cannot be predicted solely by multiplicative demographic events (e.g., birth and death), but may be substantially affected by additive processes such as internal migration and international immigration. The demographic interpretation of the values of the TL slope deserves further research.

### Other uses of TL in demographic research

This work gives a comprehensive analysis of TL for US human population data at the county level and shows the multifaceted uses of TL in extracting important socioeconomic and demographic signals. In this subsection, we address two questions. First, is the widespread (though not universal) agreement of county population distribution with TL a result of intrinsic characteristics of human populations or a purely statistical consequence of random sampling from some underlying distribution? Second, can TL be used to construct and select among alternative demographic projections?

First, Cohen and Xu [32] predicted TL parameters using random samples of probability distributions. They showed that TL's slope is proportional to the skewness of a distribution, when sampled observations are identically and independently distributed. Given the strong temporal and spatial autocorrelation in the US census data (see S1 File), the simple random sampling model by Cohen and Xu alone cannot explain TL for human population data. It is therefore necessary to construct regression models capable of incorporating the spatial and temporal structure of US county populations to analyze the statistical mechanisms of TL in this context. Alternatively, it is possible to build mathematical models to study the impact of birth, death, and migration on observed TL patterns [25,59]. The interactions between demographic stochasticity and data structure, and their contributions to TL, remain an important topic for future research.

Second, Xu et al. [36] used the spatial TL to select the most plausible projections of Norwegian human population, based on the similarity of TL slopes between data and projections. They found that the short-term population projections selected by TL agreed most closely with the recent data. Their method is purely empirical and statistical, and does not rely on any assumptions regarding population growth or migration. Their approach can be used for the US census data studied here. In addition to the spatial TL used by Xu et al. [36], the current work points to the possibility of selecting the most plausible population projection(s) using multiple TLs and QTLs. This multiplicity of approaches can be useful in case one type of TL fails to distinguish among several projections (as happened in [36]). For example, when multi-year state-level projections are provided, one could test and compare the parameters of spatial and temporal TL (or QTL) between data and projections. If multi-year county-level projections are available, one could use the spatial, temporal, and spatial hierarchical TL together to select among alternative projections.

Another potential application of TL in demographic research is to project population distributions based on TL parameters estimated from historical data. Tweedie [60], Jørgensen [61], Kendal and Jørgensen [62,63] and many other papers have explored various probability distributions that follow TL either exactly or asymptotically. If TL and appropriate parametric models can be further confirmed for human population counts or densities, then one can estimate parameters of one or several parametric probability distributions from the historical data and construct (and test) the associated abundance distribution. If multiple censuses are available, one can predict the TL parameters based on their historical trend and construct corresponding population distribution projections. This method can be tested using populations at different spatial scales (e.g., states, counties, census tracts). This approach would offer a novel but still untested way of making population projections based on the temporal or spatial population distribution in historical data described by TL.

## Supporting information

S1 File Regression diagnostics and miscellaneous testing of Taylor's law models. (DOCX)

S1 Fig Spatial variance of county population count against spatial mean of county population count on log-log scale across states within each census. (PDF)

S2 Fig Spatial variance of county population count against spatial mean of county population count on log-log scale across censuses within each state. (PDF)

S3 Fig Temporal variance of county population count against temporal mean of county population count on log-log scale across counties within each state. (PDF)

S4 Fig Regression coefficient estimates for TL and QTL using county population density. (PDF)

S5 Fig Spatial variance of county population density against spatial mean of county population density on log-log scale across states within each census. (PDF)

S6 Fig Time series of (a) the slope estimate of spatial hierarchical TL and (b) quadratic coefficient of spatial hierarchical QTL using county population density.
(PDF)

S7 Fig Spatial variance of county population density against spatial mean of county population density on log-log scale across censuses within each state. (PDF)

S8 Fig Point estimates of the slope of (a) spatial TL and (b) temporal TL, and the sign of the quadratic coefficients of (c) spatial QTL and (d) temporal QTL by US states, using county population density.
(PDF)

S9 Fig Temporal variance of county population density against temporal mean of county population density on log-log scale across counties within each state. (PDF)

S10 Fig Residuals against fitted values of the linear regression for log(spatial variance of county count) as a function of log(spatial mean of county count) across states in a given census. (PDF)

S11 Fig Residuals against fitted values of the linear regression for log(spatial variance of county density) as a function of log(spatial mean of county density) across states in a given census. (PDF)

S12 Fig Residuals against fitted values of the quadratic regression for log(spatial variance of county count) as a function of log(spatial mean of county count) across states in a given census. (PDF)

S13 Fig Residuals against fitted values of the quadratic regression for log(spatial variance of county density) as a function of log(spatial mean of county density) across states in a given census. (PDF)

S14 Fig Standardized residuals against their normal quantiles for the ols linear regression fitted to log(spatial variance of county count) as a function of log(spatial mean of county count) across states in a given census. (PDF)

S15 Fig Standardized residuals against their normal quantiles for the ols linear regression fitted to log(spatial variance of county density) as a function of log(spatial mean of county density) across states in a given census. (PDF)

S16 Fig Standardized residuals against their normal quantiles for the ols quadratic regression fitted to log(spatial variance of county count) as a function of log(spatial mean of county count) across states in a given census. (PDF)

S17 Fig Standardized residuals against their normal quantiles for the ols quadratic regression fitted to log(spatial variance of county density) as a function of log(spatial mean of county density) across states in a given census. (PDF)

S18 Fig Residuals against fitted value of the ols linear regression for log(spatial variance of county count) as a function of log(spatial mean of county count) across censuses for a given state. (PDF)

S19 Fig Residuals against fitted value of the ols linear regression for log(spatial variance of county density) as a function of log(spatial mean of county density) across censuses for a given state. (PDF)

S20 Fig Standardized residuals against their normal quantiles for the ols linear regression fitted to log(spatial variance of county count) as a function of log(spatial mean of county count) across censuses for a given state. (PDF)

S21 Fig Standardized residuals against their normal quantiles for the ols linear regression fitted to log(spatial variance of county density) as a function of log(spatial mean of county density) across censuses for a given state. (PDF)

S22 Fig Autocorrelation function against lag for the residuals of ols linear regression (up to lag 10) fitted to log(spatial variance of county count) as a function of log(spatial mean of county count) across censuses in a given state. (PDF)

S23 Fig Autocorrelation function against lag for the residuals of ols linear regression (up to lag 10) fitted to log(spatial variance of county density) as a function of log(spatial mean of county density) across censuses in a given state. (PDF)

S24 Fig Residuals against fitted value of the ols quadratic regression for log(spatial variance of county count) as a function of log(spatial mean of county count) across censuses for a given state. (PDF)

S25 Fig Residuals against fitted value of the ols quadratic regression for log(spatial variance of county density) as a function of log(spatial mean of county density) across censuses for a given state. (PDF)

S26 Fig Standardized residuals against their normal quantiles for the ols quadratic regression fitted to log(spatial variance of county count) as a function of log(spatial mean of county count) across censuses for a given state. (PDF)

S27 Fig Standardized residuals against their normal quantiles for the ols quadratic regression fitted to log(spatial variance of county density) as a function of log(spatial mean of county density) across censuses for a given state. (PDF)

S28 Fig Autocorrelation function against lag for the residuals of ols quadratic regression (up to lag 10) fitted to log(spatial variance of county count) as a function of log(spatial mean of county count) across censuses in a given state. (PDF)

S29 Fig Autocorrelation function against lag for the residuals of ols quadratic regression (up to lag 10) fitted to log(spatial variance of county density) as a function of log(spatial mean of county density) across censuses in a given state. (PDF)

S30 Fig Residuals against fitted value of the ols linear regression for log(temporal variance of county count) as a function of log(temporal mean of county count) across counties for a given state. (PDF)

S31 Fig Residuals against fitted value of the ols quadratic regression for log(temporal variance of county count) as a function of log(temporal mean of county count) across counties for a given state. (PDF)

S32 Fig Standardized residuals against their normal quantiles for the ols linear regression fitted to log(temporal variance of county count) as a function of log(temporal mean of county count) across counties for a given state. (PDF)

S33 Fig Standardized residuals against their normal quantiles for the ols quadratic regression fitted to log(temporal variance of county count) as a function of log(temporal mean of county count) across counties for a given state. (PDF)

S34 Fig Residuals against fitted value of the ols linear regression for log(temporal variance of county density) as a function of log(temporal mean of county density) across counties for a given state. (PDF)

S35 Fig Residuals against fitted value of the ols quadratic regression for log(temporal variance of county density) as a function of log(temporal mean of county density) across counties for a given state. (PDF)

S36 Fig Standardized residuals against their normal quantiles for the ols linear regression fitted to log(temporal variance of county density) as a function of log(temporal mean of county density) across counties for a given state. (PDF)

S37 Fig Standardized residuals against their normal quantiles for the ols quadratic regression fitted to log(temporal variance of county density) as a function of log(temporal mean of county density) across counties for a given state. (PDF)

S38 Fig Spatial variance of county count against spatial mean of county count across states in each census. (PDF)

S39 Fig Spatial variance of county density against spatial mean of county density across states in each census. (PDF)

S40 Fig Spatial variance of county count against spatial mean of county count across censuses in each state that occurs in at least 5 censuses. (PDF)

S41 Fig Spatial variance of county density against spatial mean of county density across censuses in each state that occurs in at least 5 censuses. (PDF)

S42 Fig Point estimates of the slope ((a) and (b)) of ols linear regressions and the sign of the quadratic coefficient ((c) and (d)) of ols quadratic regressions for spatial TL for each state that occurs in at least 5 censuses, using count ((a) and (c)) and density ((b) and (d)).
(PDF)

S43 Fig Temporal variance of county count against temporal mean of county count across counties in each of 36 states. (PDF)

S44 Fig Temporal variance of county density against temporal mean of county density across counties in each of 36 states. (PDF)

S45 Fig Point estimates of the slope ((a) and (b)) of ols linear regressions and the sign of the quadratic coefficient ((c) and (d)) of ols quadratic regressions for spatial TL for each state, using count ((a) and (c)) and density ((b) and (d)).
(PDF)

S46 Fig Scatterplots of the point estimate of *b* of the spatial hierarchical TL (shtl) and *e* of the spatial hierarchical quadratic TL (shqtl) using count, with (vertical axis) and without (horizontal axis) the minimum number of sampling units requirement (*n* ≥ 15). (PDF)

S47 Fig Scatterplots of the point estimate of *b* of the spatial hierarchical TL (shtl) and *e* of the spatial hierarchical quadratic TL (shqtl) using density, with (vertical axis) and without (horizontal axis) the minimum number of sampling units requirement (*n* ≥ 15). (PDF)

S48 Fig Scatterplots of the point estimate of *b* of the spatial TL (stl) and *e* of the spatial quadratic TL (sqtl) using count, with (vertical axis) and without (horizontal axis) the minimum number of sampling units requirement (*n* ≥ 15). (PDF)

S49 Fig Scatterplots of the point estimate of *b* of the spatial TL (stl) and *e* of the spatial quadratic TL (sqtl) using density, with (vertical axis) and without (horizontal axis) the minimum number of sampling units requirement (*n* ≥ 15). (PDF)

S50 Fig Scatterplots of the point estimate of *b* of the temporal TL (ttl) and *e* of the temporal quadratic TL (tqtl) using count, with (vertical axis) and without (horizontal axis) the minimum number of sampling units requirement (*n* ≥ 15). (PDF)

S51 Fig Scatterplots of the point estimate of *b* of the temporal TL (ttl) and *e* of the temporal quadratic TL (tqtl) using density, with (vertical axis) and without (horizontal axis) the minimum number of sampling units requirement (*n* ≥ 15). (PDF)

S1 Table Ordinary least-squares (ols) linear regression statistics of spatial hierarchical Taylor's law, using count and density separately. (XLSX)

S2 Table Ordinary least-squares (ols) quadratic regression statistics of spatial hierarchical quadratic Taylor's law, using count and density separately. (XLSX)

S3 Table Ordinary least-squares (ols) linear regression statistics of spatial Taylor's law, using count and density separately. (XLSX)

S4 Table Ordinary least-squares (ols) quadratic regression statistics of spatial quadratic Taylor's law, using count and density separately. (XLSX)

S5 Table Ordinary least-squares (ols) linear regression statistics of temporal Taylor's law, using count and density separately. (XLSX)

S6 Table Ordinary least-squares (ols) quadratic regression statistics of temporal quadratic Taylor's law, using count and density separately. (XLSX)

S7 Table Summary of the regression statistics of the Taylor's law (TL) (Eq 1) and quadratic Taylor's law (QTL) (Eq 2) models using county population density. (XLSX)

S8 Table Summary statistics of county area, county count and county density in each state and census. (XLSX)

S9 Table Diagnostic statistics of the ols linear regression for spatial hierarchical Taylor's law, using count and density separately. (XLSX)

S10 Table Diagnostic statistics of the ols quadratic regression for spatial hierarchical quadratic Taylor's law, using count and density separately. (XLSX)

S11 Table Diagnostic statistics of the ols linear regression for spatial Taylor's law, using count and density separately. (XLSX)

S12 Table Diagnostic statistics of the ols quadratic regression for spatial quadratic Taylor's law, using count and density separately. (XLSX)

S13 Table Diagnostic statistics of the ols linear regression for temporal Taylor's law, using count and density separately. (XLSX)

S14 Table Diagnostic statistics of the ols quadratic regression for temporal quadratic Taylor's law, using count and density separately. (XLSX)

S15 Table Ordinary least-squares (ols) linear regression statistics of the spatial hierarchical TL with the minimum number of sampling units requirement (n ≥ 15), using count and density separately. (XLSX)

S16 Table Ordinary least-squares quadratic regression statistics of the spatial hierarchical quadratic Taylor's law (QTL) with the minimum number of sampling units requirement (n ≥ 15), using count and density separately. (XLSX)

S17 Table Ordinary least-squares linear regression statistics of the spatial TL with the minimum number of sampling units requirement (n ≥ 15), using count and density separately. (XLSX)

S18 Table Ordinary least-squares quadratic regression statistics of the spatial QTL with the minimum number of sampling units requirement (n ≥ 15), using count and density separately. (XLSX)

S19 Table Ordinary least-squares linear regression statistics of the temporal TL with the minimum number of sampling units requirement (n ≥ 15), using count and density separately. (XLSX)

S20 Table Ordinary least-squares quadratic regression statistics of the temporal QTL with the minimum number of sampling units requirement (n ≥ 15), using count and density separately. (XLSX)

S21 Table Diagnostic statistics of the ols linear regression for the spatial hierarchical TL with the minimum number of sampling units requirement (n ≥ 15), using count and density separately. (XLSX)

S22 Table Diagnostic statistics of the ols quadratic regression for the spatial hierarchical QTL with the minimum number of sampling units requirement (n ≥ 15), using count and density separately. (XLSX)

S23 Table Diagnostic statistics of the ols linear regression for the spatial TL with the minimum number of sampling units requirement (n ≥ 15), using count and density separately. (XLSX)

S24 Table Diagnostic statistics of the ols quadratic regression for the spatial QTL with the minimum number of sampling units requirement (n ≥ 15), using count and density separately. (XLSX)

S25 Table Diagnostic statistics of the ols linear regression for the temporal TL with the minimum number of sampling units requirement (n ≥ 15), using count and density separately. (XLSX)

S26 Table Diagnostic statistics of the ols quadratic regression for the temporal QTL with the minimum number of sampling units requirement (n ≥ 15), using count and density separately. (XLSX)

## References

[1] Gordon P, Richardson HW, Wong HL. The distribution of population and employment in a polycentric city: the case of Los Angeles. Environ Plann A. 1986;18: 161–173. 10.1068/a180161 12340568

[2] Plane DA, Rogerson PA. The geographical analysis of population: with applications to planning and business. London: Wiley; 1994.

[3] Alberti M, Marzluff JM, Shulenberger E, Bradley G, Ryan C, Zumbrunnen C. Integrating humans into ecology: opportunities and challenges for studying urban ecosystems. BioScience. 2003;53: 1169–1179.

[4] Grieco EM, Trevelyan E, Larsen L, Acosta YD, Gambino C, de la Cruz P, et al The size, place of birth, and geographic distribution of the foreign-born population in the United States: 1960 to 2010. US Census Bureau, Population Division Working Paper. 2012;96 Available from: https://www.census.gov/content/dam/Census/library/working-papers/2012/demo/POP-twps0096.pdf

[5] Giles-Corti B, Vernez-Moudon A, Reis R, Turrell G, Dannenberg AL, Badland H, et al City planning and population health: a global challenge. Lancet. 2016;388: 2912–2924. 10.1016/S0140-6736(16)30066-6 27671668

[6] Meyer WB, Turner BL. Human population growth and global land-use/cover change. Annu Rev Ecol Syst. 1992;23: 39–61.

[7] Cardillo M, Purvis A, Sechrest W, Gittleman JL, Bielby J, Mace JM. Human population density and extinction risk in the world's carnivores. PLoS Biol. 2004;2: e197 10.1371/journal.pbio.0020197 15252445 PMC449851

[8] Cincotta RP, Wisnewski J, Engelman R. Human population in the biodiversity hotspots. Nature. 2000;404: 990–992. 10.1038/35010105 10801126

[9] Bowman DMJS, Balch J, Artaxo P, Bond WJ, Cochrane MA, D'antonio CM, et al The human dimension of fire regimes on Earth. J Biogeogr. 2011;38: 2223–2236. 10.1111/j.1365-2699.2011.02595.x 22279247 PMC3263421

[10] Hales S, de Wet N, Maindonald J, Woodward A. Potential effect of population and climate changes on global distribution of dengue fever: an empirical model. Lancet. 2002;360: 830–834. 10.1016/S0140-6736(02)09964-6 12243917

[11] McGranahan G, Balk D, Anderson B. The rising tide: assessing the risks of climate change and human settlements in low elevation coastal zones. Environ Urban. 2007;19: 17–37.

[12] Patz JA, Campbell-Lendrum D, Holloway T, Foley JA. Impact of regional climate change on human health. Nature. 2005;438: 310–317. 10.1038/nature04188 16292302

[13] Yntema DB. Measures of the inequality in the personal distribution of wealth or income. J Am Stat Assoc. 1933;28: 423–433.

[14] Duncan OD. The measurement of population distribution. Pop Stud-J Demog. 1957;11: 27–45.

[15] Eisler Z, Bartos I, Kertész J. Fluctuation scaling in complex systems: Taylor's law and beyond. Adv Phys. 2008;57: 89–142.

[16] Taylor RAJ. Taylor's power law: order and pattern in nature. Cambridge: Elsevier Academic Press; 2019.

[17] Bliss CI. Statistical problems in estimating populations of Japanese beetle larvae. J Econ Entomol. 1941;34: 221–232.

[18] Fracker SB, Brischle HA. Measuring the local distribution of Ribes. Ecology. 1944;25: 283–303.

[19] Hayman BI, Lowe AD. The transformation of counts of the cabbage aphid (*Brevicoryne brassicae* (L.)). New Zeal J Sci. 1961;4: 271–278.

[20] Taylor LR. Aggregation, variance and the mean. Nature. 1961;189: 732–735.

[21] Taylor LR, Woiwod IP, Perry JN. The density-dependence of spatial behaviour and the rarity of randomness. J Anim Ecol. 1978;47: 383–406.

[22] Taylor LR, Woiwod IP. Temporal stability as a density-dependent species characteristic. J Anim Ecol. 1980;49: 209–224.

[23] Taylor LR, Woiwod IP, Perry JN. Variance and the large scale spatial stability of aphids, moths and birds. J Anim Ecol. 1980;49: 831–854.

[24] Taylor LR, Woiwod IP. Comparative synoptic dynamics. I. Relationships between inter-and intra-specific spatial and temporal variance/mean population parameters. J Anim Ecol. 1982;51: 879–906.

[25] Anderson RM, Gordon DM, Crawley MJ, Hassell MP. Variability in the abundance of animal and plant species. Nature. 1982;296: 245–248.

[26] Hanley QS, Khatun S, Yosef A, Dyer RM. Fluctuation scaling, Taylor’s law, and crime. PLoS One. 2014;9: e109004 10.1371/journal.pone.0109004 25271781 PMC4182799

[27] Cai Q, Xu HC, Zhou WX. Taylor’s law of temporal fluctuation scaling in stock illiquidity. Fluct Noise Lett. 2016;15: 1650029.

[28] Cohen JE. Statistics of primes (and probably twin primes) satisfy Taylor's law from ecology. Am Stat. 2016;70: 399404.

[29] Tippett MK, Cohen JE. Tornado outbreak variability follows Taylor’s power law of fluctuation scaling and increases dramatically with severity. Nat Commun. 2016;7: 10668 10.1038/ncomms10668 26923210 PMC4773447

[30] Taylor LR, Taylor RAJ. Aggregation, migration and population mechanics. Nature. 1977;265: 415–421. 10.1038/265415a0 834291

[31] Kilpatrick AM, Ives AR. Species interactions can explain Taylor's power law for ecological time series. Nature. 2003;422: 65–68. 10.1038/nature01471 12621433

[32] Cohen JE, Xu M. Random sampling of skewed distributions implies Taylor’s power law of fluctuation scaling. P Natl Acad Sci USA. 2015;112: 7749–7754. 10.1073/pnas.1503824112 PMC4485080 25852144

[33] Benassi F, Naccarato A. Modelling the spatial variation of human population density using Taylor’s power law, Italy, 1971–2011. Reg Stud. 2018;53: 206–216.

[34] Cohen JE, Xu M, Brunborg H. Taylor's law applies to spatial variation in a human population. Genus. 2013;69: 25–60.

[35] Naccarato A, Benassi F. On the relationship between mean and variance of world's human population density: A study using Taylor's power law. LSRS. 2018;11: 307–314.

[36] Xu M, Brunborg H, Cohen JE. Evaluating multi-regional population projections with Taylor’s law of mean–variance scaling and its generalization. J Popul Res. 2017;34: 79–99.

[37] Bliss CI, Owen ARG. Negative binomial distributions with a common k. Biometrika. 1958;45: 37–58.

[38] Manson S, Schroeder J, Riper DV, Ruggles S. PUMS National Historical Geographic Information System: Version 13.0; 2018 [cited 2018 July 19]. Minneapolis: University of Minnesota Available from: 10.18128/D050.V13.0

[39] Champion T. Urbanization, suburbanization, counterurbanization and reurbanization In: Paddison R editor. Handbook of urban studies. SAGE Publications Ltd; 2000 pp. 143–161.

[40] Taylor LR, Perry JN, Woiwod IP, Taylor RAJ. Specificity of the spatial power-law exponent in ecology and agriculture. Nature. 1988;332: 721–722.

[41] R Core Team. R: a language and environment for statistical computing. R Foundation for Statistical Computing, Vienna, Austria 2018 URL https://www.R-project.org/.

[42] Coon RC, Leistritz FL. The state of North Dakota: economic, demographic, public service, and fiscal conditions. Department of Agribusiness and Applied Economics, North Dakota State University, Fargo, ND 1998.

[43] Murphy W. fiftystater: map data to visualize the fifty U.S. states with Alaska and Hawaii insets. R package version 1.0.1. 2016. URL https://CRAN.R-project.org/package=fiftystater.

[44] Schwieder D. History of Iowa. Iowa Official Register. 2015;7:1.

[45] McLeman R. Migration out of 1930s rural eastern Oklahoma: insights for climate change research. Great Plains Quart. 2006;26: 2740.

[46] Bonifazi C, Heins F. Long-term trends of internal migration in Italy. Int J Popul Geogr. 2000;6: 111–131.

[47] Renkow M, Hoover D. Commuting, migration, and rural-urban population dynamics. J Reg Sci. 2000;40: 261–287.

[48] Ambinakudige S, Parisi D. A spatiotemporal analysis of inter-county migration patterns in the United States. Appl Spat Anal Policy. 2017;10: 121–137.

[49] Berry BJ, Dahmann DC. Population redistribution in the United States in the 1970s. Popul Dev Rev. 1977;3: 443–471.

[50] Long L, DeAre D. Repopulating the countryside: a 1980 census trend. Science. 1982;217: 1111–1116. 10.1126/science.217.4565.1111 17740957

[51] Long L, DeAre D. US population redistribution: a perspective on the nonmetropolitan turnaround. Popul Dev Rev. 1988;14: 433–450.

[52] Fuguitt GV. The nonmetropolitan population turnaround. Annu Rev Sociol. 1985;11: 259–280. 10.1146/annurev.so.11.080185.001355 12313950

[53] Johnson KM, Beale CL. The recent revival of widespread population growth in nonmetropolitan areas of the United States. Rural Sociol. 1994;59: 655–667.

[54] Lee S, Seo JG, Webster C. The decentralising metropolis: economic diversity and commuting in the US suburbs. Urban Stud. 2006;43: 2525–2549.

[55] Lang R, Knox PK. The new metropolis: Rethinking megalopolis. Reg Stud. 2009;43: 789–802.

[56] Vining DR Jr, Strauss A. A demonstration that the current deconcentration of population in the United States is a clean break with the past. Environ Plan A. 1977;9: 751–758. 10.1068/a090751 12310795

[57] Gordon P. Deconcentration without a 'clean break'. Environ Plan A. 1979;11, 281–289. 10.1068/a110281 12262970

[58] Johnson KM, Lichter DT. Rural depopulation: growth and decline processes over the past century. Rural Sociol. 2019;84: 3–27.

[59] Jiang J, DeAngelis DL, Zhang B, Cohen JE. Population age and initial density in a patchy environment affect the occurrence of abrupt transitions in a birth-and-death model of Taylor's law. Ecol Modell. 2014;289: 59–65.

[60] Tweedie MCK. An index which distinguishes between some important exponential families. Proceedings of the Indian Statistical Institute Golden Jubilee International Conference on Statistics: Applications and New Directions. 1984;579: 579–604.

[61] Jørgensen B. The theory of dispersion models. London: CRC Press; 1997.

[62] Kendal WS, Jørgensen B. Taylor's power law and fluctuation scaling explained by a central-limit-like convergence. Phys Rev E. 2011a;83: 066115. 10.1103/PhysRevE.83.066115 21797449

[63] Kendal WS, Jørgensen B. Tweedie convergence: A mathematical basis for Taylor's power law, 1/f noise, and multifractality. Phys Rev E. 2011b;84: 066120. 10.1103/PhysRevE.84.066120 22304168

