# Supplementary material for: Analyzing and interpreting spatial and temporal variability of the United States county population distributions using Taylor's law
Source: PLoS One. 2019 Dec 11;14(12):e0226096. doi: 10.1371/journal.pone.0226096 (PMC6905577; doi:10.1371/journal.pone.0226096)
Supplement: S1 File — (DOCX) [file pone.0226096.s001.docx]

# Full title: Analyzing and interpreting spatial and temporal variability of US county population distributions using Taylor's law

# Short title: Fluctuation scaling of US county populations

Meng Xu^1^ and Joel E. Cohen^2, 3, 4, *^

^1^Department of Mathematics, Pace University, 41 Park Row, New York, NY 10038, USA

^2^Laboratory of Populations, The Rockefeller University and Columbia University, 1230 York Avenue, Box 20, New York, NY 10065, USA

^3^Earth Institute and Department of Statistics, Columbia University, New York, NY 10027

^4^Department of Statistics, University of Chicago, Chicago, IL 60637

^*^Corresponding author

E-mail: [cohen@mail.rockefeller.edu](mailto:cohen@mail.rockefeller.edu)

# S1 File. REGRESSION DIAGNOSTICS AND MISCELLANEOUS TESTING OF TAYLOR'S LAW MODELS

**Table of Contents**

[REGRESSION DIAGNOSTICS AND MISCELLANEOUS TESTING OF TAYLOR'S LAW MODELS 1](#_Toc15548444)

[Taylor's law for county population density 3](#_Toc15548445)

[Spatial hierarchical Taylor's law 3](#_Toc15548446)

[Spatial Taylor's law 3](#_Toc15548447)

[Temporal Taylor's law 4](#_Toc15548448)

[Regression diagnostics of Taylor's law models 5](#_Toc15548449)

[Methods 5](#_Toc15548450)

[Spatial hierarchical Taylor's law 7](#_Toc15548451)

[Spatial Taylor's law 8](#_Toc15548452)

[Temporal Taylor's law 9](#_Toc15548453)

[Summary of regression diagnostics of Taylor's law models 10](#_Toc15548454)

[Taylor's law analysis for US county abundance with minimum number of sampling units 12](#_Toc15548455)

[Regression estimates and models of Taylor's law 13](#_Toc15548456)

[Ordinary least-squares regression diagnostics 14](#_Toc15548457)

[Comparison with Taylor's law tested without sampling unit requirement 16](#_Toc15548458)

[Summary of the effects of required minimum sample size on estimates of b 17](#_Toc15548459)

[Supporting references 18](#_Toc15548460)

[Supporting figures and figure legends 20](#_Toc15548461)

[Supporting table captions 77](#_Toc15548462)

# Taylor's law for county population density

The main text tests forms of Taylor's law (TL) for counts (absolute numbers of people) of US counties. Here we test forms of TL for the population densities (people⋅km^-2^) of US counties.

## **Spatial hierarchical Taylor's law**

The slope of spatial hierarchical Taylor's law (TL) showed less variation than that of the spatial and temporal TL (S4 Fig), with values ranging from 1.89 to 2.52 (compared to the range from 0.91 to 5.12 for the spatial TL and from 1.38 to 3.32 for the temporal TL). Without Bonferroni correction, the spatial hierarchical TL and QTL describe the mean-variance relationship well in respectively 20 and three (1820, 1850 and 1860) of the 23 censuses (S5 Fig, S1 and S2 Tables). With Bonferroni correction, the spatial hierarchical TL and QTL describe the relationship well in respectively 22 and one (1860) census. The average adj *R*^2^ across censuses was 0.86 for TL and 0.87 for QTL (S7 Table).

TL slope fluctuated during the 19th century and has been declining since the 1900 census (S6 Fig), similar to the results based on county population count. The quadratic coefficient of QTL has been stable since the 1870 census and stays around zero. According to 1-adj. *R*^2^, the cross-state dissimilarity in the spatial distribution of county population density was greatest in the 1790 census and smallest in the 1880 census, with 1-adj. *R*^2^ equaling 0.36 and 0.10 respectively (S1 and S2 Tables).

## **Spatial Taylor's law**

Mean-variance relationship across censuses varied among states (S4 Fig). Spatial TL reasonably described the relationship in 22 and 18 states with and without Bonferroni correction respectively (S7 Fig, S3 and S4 Tables). Spatial QTL described the relationship well in 28 and 36 states respectively with and without Bonferroni correction. Neither TL nor QTL was a good model in five (AKT, NMT, ND, SD, UTT) and one (ND) state with and without Bonferroni correction. The average adj. *R*^2^ across states was 0.91 for the spatial TL and 0.94 for the spatial QTL (S7 Table).

Geographically, slope of the spatial TL was highest in the middle of the country (S8a Fig). The quadratic coefficient of the spatial QTL was negative in the states on or near the Northeast and West coast (S8c Fig), indicating that the spatial heterogeneity of county population density was diminishing over time in these developed states. Based on 1-adj. *R*^2^ of the best model, the temporal dissimilarity of spatial distribution of county population density was greatest in MT, AKT and SD (with 1-adj. *R*^2^ equaling 0.35, 0.24 and 0.24 respectively), and smallest in AZT, CT and NV (with 1-adj. *R*^2^ equaling 0) (S3 and S4 Tables).

## **Temporal Taylor's law**

Temporal TL described well the mean-variance relationship across counties for majority of states (respectively 47 and 34 states with and without Bonferroni correction) (S9 Fig, S5 and S6 Tables). Temporal QTL described the relationship well in eight and 29 states respectively with and without Bonferroni correction. Neither model described the relationship well in nine (AKT, ART, DC, FLT, HIT IDT, MTT, RI, WYT) and one (AKT) state respectively with and without Bonferroni correction. The average adj. *R*^2^ across states was 0.84 for the temporal TL and QTL (S7 Table).

Slope of the temporal TL was highest in states located in the Midwest and South (S8b Fig), while the quadratic coefficient of the temporal QTL was not significantly different from zero in most states after Bonferroni correction (S8d Fig). 1-adj. *R*^2^ showed that the cross-county dissimilarity of temporal distribution of county population density was greatest in AKT, FLT and NMT (with 1-adj. *R*^2^ equaling 0.81, 0.55 and 0.54 respectively), and smallest in WYT, NH and MA (with 1-adj. *R*^2^ equaling 0.00, 0.02 and 0.02 respectively) (S5 and S6 Tables).

The summary statistics (i.e. mean, variance, skewness, kurtosis, covariance and correlation) of county area, county population count and density in each state and census are available in S8 Table.

# Regression diagnostics of Taylor's law models

We describe our methods of analysis and the detailed results of applying these methods to the spatial hierarchical, spatial and temporal forms of TL. At the end of this section, we summarize its principal findings.

## **Methods**

We use various statistical graphs and significance tests to evaluate the assumptions of the linear (Eq 1) and quadratic (Eq 2) models. First, we check if any outliers exist in each model fitting. Second, we evaluate the basic assumptions of ordinary least-squares (ols) regressions including normality and homoscedasticity of model residuals, and linearity between log(variance) and log(mean) for the linear model. Then, we test the spatial or temporal correlation in the model residuals.

For each combination of TL type (spatial hierarchical, spatial or temporal), abundance measure (count or density), and model (linear or quadratic), we use two sets of graphs to examine the linearity between log(variance) and log(mean) and the normality of regression residuals. First, we plot residuals against fitted values from each testing of TL to evaluate the homoscedasticity of model residuals and the linearity between log(variance) and log(mean). If the range of points in the vertical direction is varies little at different fitted values, then it indicates that the model residuals have constant variance and their homoscedasticity is not rejected. If the smoothed fitting line of residuals against fitted values does not show clear trend, then it means that the linearity between log(variance) and log(mean) is not rejected. Second, we use a Q-Q plot to examine the normality of model residuals by comparing the scatterplot of standardized residuals against theoretical normal quantiles with a straight line of slope one. Systematic deviation of the points from the straight line suggests non-normality. All plots are made using the autoplot function in R 3.4.4 (R Core Team 2018). Results based on these diagnostic graphs are subjective and may be inconclusive.

We also test quantitatively the regression model assumptions for each combination of TL type, abundance measure and model. First, we use the Bonferroni outlier test to identify any outliers in the data (Cook and Weisberg 1982). We use the Shapiro-Wilk test (Royston 1982), skewness test and kurtosis tests from the gvlma package (Peña and Slate 2006) in R (R Core Team 2018) to test the normality of regression residuals. We check residual homoscedasticity using the Breusch-Pagan test (Breusch and Pagan 1979) and the heteroscedasticity test from the gvlma package in R. We apply the global test from the gvlma package to evaluate the overall appropriateness of the regression model assumptions.

The spatial TL specifies linear dependence of log(spatial variance) on log(spatial mean) across censuses within a state. To test whether temporal correlation is present among residuals of a regression model, we plot the temporal empirical autocorrelation function of the residuals and use the Durbin-Watson test (Durbin and Watson 1950) to check their temporal autocorrelation at lag 1.

We use Moran's I (Cliff and Ord 1981) to determine whether the spatial correlation is significant. The spatial hierarchical TL specifies linear dependence of log(spatial variance of counties) on log(spatial mean of counties) across states at a fixed census. Spatial correlation may be present among states within a census. For the spatial hierarchical TL, Moran's I is calculated separately at two spatial distances: 750 km and 1000 km. The temporal TL specifies linear dependence of log(temporal variance of counties) on log(temporal mean of counties) across counties in a given state. Spatial correlation may be present among counties within a state. For the temporal TL, Moran's I is calculated separately at two spatial distances: 250 km and 500 km. We use different spatial distances in Moran's I to accommodate the typical size differences of states versus counties and to check the sensitivity of Moran's I to spatial scales.

## **Spatial hierarchical Taylor's law**

Visually, using count and density, residual plots supported the linearity between log(mean) and log(variance) in the later censuses (with no clear pattern) (S10 and S11 Figs). The quadratic model (Eq 2) reduced slightly any systematic trend in the residual plots (S12 and S13 Figs). The vertical spread of residual points was roughly similar across different fitted values. Q-Q plots showed better agreement of residuals to a normal distribution in the later censuses than in the earlier censuses, regardless of measure or model (S14-S17 Figs). Significance tests of Moran's I for the ols linear regressions of the spatial hierarchical TL revealed strong spatial correlation in recent censuses, for both measures (count and density) and models (linear and quadratic) (S9 and S10 Tables). Using count, the global test rejected the overall appropriateness of the assumptions of the linear model (Eq 1) for the 1820 census and 1900 census. Specifically, in 1820, the Shapiro-Wilk test and kurtosis test rejected the normality of residuals. In 1900, the Shapiro-Wilk test, skewness test and kurtosis test rejected the normality of residuals. In addition, both censuses showed outliers (DE in 1820, 1830 and AKT in 1900). The homoscedasticity test from the gvlma function in R rejected the homoscedasticity of residuals in 1900. The Breusch-Pagan test rejected the homoscedasticity of residuals in 1800 (S9 Table). Except for spatial correlation, no assumption was violated for the linear model and no outliers were found using density. The same results held for the quadratic model (Eq 1) using count and density, except that in 1800 no assumption was significantly violated and Massachusetts was an outlier when using count (S10 Table).

## **Spatial Taylor's law**

Residual plots for the linear model showed different patterns among states, using count and density (e.g. quadratic trends in CT and NJ; cubic trends in OH and TX) (S18 and S19 Figs). We cannot evaluate the homoscedasticity of residuals due to the lack of repeated values at large fitted log(variance) from the residual plots. Q-Q plots showed substantial deviation from normality in MS, NJ and RI for count, and in AR, CT and MN for density (S20 and S21 Figs). Based on the Durbin-Watson test, the ols linear regression showed significant first-order temporal autocorrelation in 39 states using count and 42 states using density (S11 Table, S22 and S23 Figs). The Bonferroni outlier test revealed outliers in 12 states using count and 12 states using density. Using count, hypothesis tests rejected normality or homoscedasticity in four of the 55 states. Specifically, homoscedasticity of residuals was rejected for MA and VA. Normality was rejected for NH and VA. Using count, the global test rejected the appropriateness of the linear model assumptions in eight states: CO, CT, DE, ID, MA, MD, PA, RI and VA. Using density, significance tests did not reject normality or homoscedasticity in any state. Outliers existed in CO (1850), MA (1790) and NE (1870). The global test rejected the overall appropriateness of the linear model assumptions for density in CT, DE, LA, MA, NJ, NY and VA (S11 Table).

For the ols quadratic model, the residual plots showed cubic polynomial trend in most states and no clear trend in other states (e.g. LA and VA for count; DE and NV for density) (S24 and S25 Figs). Again, we cannot determine the presence of heteroscedasticity from the residual plots due to the uneven distribution of residuals across fitted values. Q-Q plots supported the normality of residuals in all states except ID and IL for count, and except MI and MS for density (S26 and S27 Figs). The Durbin-Watson test showed significant first-order temporal correlation in 27 states using count and 35 states using density (S12 Table, S28 and S29 Figs). The Bonferroni outlier test identified outliers in 13 states using count and 14 states using density. Using count, significance tests show that six states violate normality or homoscedasticity. Normality is rejected in DE, MA, ME, NH and VA. Homoscedasticity is rejected in CO, DE, MA, ME, NH and VA. The overall appropriateness of the quadratic model is rejected in CT, DE, IL, MA, ME, MT, NH, NY, OR, PA and VA. Using density, significance tests show that DE is the only state that violates normality or homoscedasticity. The overall appropriateness of the quadratic model assumptions is rejected in DE, MN, NJ and OR (S12 Table).

## **Temporal Taylor's law**

Using count, residuals of both the linear and quadratic models were randomly scattered in most states except a few states with small number of counties. For both models, the vertical spread of residuals seemed to be wider at the small or intermediate fitted values for some states (e.g. GA and TX) and cannot be compared for other states due to small number of counties (e.g. AKT and RI) (S30 and S31 Figs). Q-Q plots showed good agreement to normality in all states except AK, CA, NJ, NV and UT for the linear model, and except AK, CA, IDT, and MTT for the quadratic model (S32 and S33 Figs). Using significance tests, outliers were present in 18 states. Normality of the ols linear regression residual was rejected in CA, FL, NE, OH, SD, UTT and VA. Homoscedasticity of the ols linear regression residual was rejected in FL only. The global test rejected the appropriateness of the linear model assumptions in 13 states. Moran's I of the ols linear model was significant in six states (for distance = 500 km) and in 12 states (for distance = 250 km) (S13 Table). For the ols quadratic model, outliers were present in 18 states. Normality was rejected in nine states. In no state was the homoscedasticity assumption violated. The global test rejected the appropriateness of the quadratic model assumptions in 12 states. Moran's I of the ols quadratic model was significant in seven states (for distance = 500 km) and in 11 states (for distance = 250 km) (S14 Table). Four states contained at least one outlier: FL (Miami-Dade county), SD (Pine Ridge Indian Reservation), UTT (Morgan County) and VA (Barbour County), for both the linear and quadratic models.

Using density, residual plots did not show clear patterns that rejected the linear or quadratic models in most states, except in a few states with few counties (S34 and S35 Figs). Q-Q plots indicated no systematic deviation from normality in most states for both models (S36 and S37 Figs). Significance tests identified outliers in 21 states. Normality of the ols linear model was rejected in 12 states. Homoscedasticity was rejected in six states. The global test rejected the appropriateness of the linear model assumptions in 17 states. Moran's I of the linear model was significant in KS and TX (for distance = 500 km) and in seven states (for distance = 250 km) (S13 Table). For the quadratic model, outliers were identified in 16 states. Normality was rejected in 13 states and homoscedasticity was rejected in CA only. The global test rejected the appropriateness of the quadratic model assumptions in 12 states. Moran's I of the quadratic model was significant in KS, MN and TX (for distance = 500 km) and in seven states (for distance = 250 km) (S14 Table).

## **Summary of regression diagnostics of Taylor's law models**

Overall, the spatial hierarchical TL is a more suitable model than the spatial TL and temporal TL in quantifying the mean-variance relationships of county population distribution. Based on the global test, with Bonferroni correction, the assumptions of spatial hierarchical TL are not rejected in 21 and 23 of the 23 censuses (≈ 91% and 100%) respectively using count and density. In comparison, the assumptions of spatial TL are not rejected in 44 and 48 of the 55 states (≈ 80% and 87%) respectively using count and density. The assumptions of temporal TL are not rejected in 51 and 47 of the 64 states (≈ 80% and 73%) respectively using count and density.

Within each type of TL, the quadratic extension does not improve substantially the quality of the linear model in terms of assumption validity, for count and density separately. Specifically, with Bonferroni correction, the global test does not reject the assumptions of spatial hierarchical QTL in 21 and 23 of the 23 censuses (≈ 91% and 100%) respectively using count and density. The assumptions of spatial QTL are not rejected in 44 and 51 of the 55 states (≈ 80% and 93%) respectively using count and density. The assumptions of temporal TL are not rejected in 52 and 52 of the 64 states (≈ 81% and 81%) respectively using count and density.

The spatial and temporal autocorrelation of model residuals reveals distinct features among three types of TL. For the spatial hierarchical TL and QTL, regardless of count and density, the spatial autocorrelation is significant in most censuses in the 20th century, probably because the counterurbanization of the US populations enhances the spatial connectivity of county populations. For the spatial TL, for both count and density, the temporal autocorrelation within residuals is significant in most states. The spatial QTL accommodates temporal structure and reduced the temporal autocorrelation within residuals. The spatial autocorrelation within residuals of the temporal TL and QTL occurred in a minority of states, regardless of abundance measure. The presence of spatial and temporal autocorrelation in most TL testing suggests that new models for TL (e.g. generalized least-squares) should be invoked to analyze the mean-variance relationship. This point is a topic for future research.

# Taylor's law analysis for US county abundance with minimum number of sampling units

To analyze the effect of sample size on the three types of TL, we adopted the recommendations by Taylor et al. (1988) and excluded any mean-variance pairs that were calculated from fewer than 15 sampling units. Here we defined a sampling unit as a unique combination of county and census. Specifically, in the spatial hierarchical and spatial TL, we excluded any state in a given census having fewer than 15 counties with finite non-zero counts or densities. In the temporal TL, we excluded any county in a state having fewer than 15 censuses with finite non-zero counts or densities. In addition, we excluded any census (for the spatial hierarchical TL) or state (for the spatial and temporal TL) that contained fewer than five finite and positive log(mean)-log(variance) pairs. We repeated the ols regression fitting and diagnostic checking for these new sets of means and variances. The requirement of at least a minimum number of sampling units reduced the number of states used in testing the spatial TL and temporal TL (compared to the main text).

The results summarized below show clear effects of having at least a minimum number of observations. For the spatial hierarchical TL, using count or density, none of the ols quadratic models (Eq 2) yielded a significantly nonzero quadratic coefficient, i.e., the success of TL in describing the data improved. For both count and density, the linear model (or TL) provided a reasonable description of the relationship between spatial mean and spatial variance across states in a given census. Similarly, county abundance obeyed the spatial TL (with no significant evidence for nonlinearity) in a large majority of 42 states, and for the temporal TL, among the ols quadratic regressions, there was no significant evidence against linearity in 32 of the 36 states. Overall, the imposition of a minimum data requirement improved the success of the linear forms of TL, for both measures of abundance and all three types of TL.

## **Regression estimates and models of Taylor's law**

For the spatial hierarchical TL, using count, the ols linear model (Eq 1) showed significantly positive *b* in all 23 censuses except 1790 (p-value = 0.0359 > 0.05/23 = 0.0022) (S38 Fig, S15 Table). Excluding the 1790 census, the point estimate of *b* ranged from 1.85 (1860) to 2.81 (1800) and adj. *R*^2^ ranged from 0.74 to 0.93. None of the ols quadratic models (Eq 2) yielded significantly nonzero quadratic coefficient *e* (S16 Table). Using density, ols linear model yielded significantly positive *b* in all censuses (p-value ≤ 0.0001) and its estimates ranged from 2.01 (2010) to 3.52 (1800) (S39 Fig, S16 Table). Adj. *R*^2^ of the ols linear regression ranged from 0.83 to 0.96. None of the quadratic models yielded significantly nonzero *e* (S16 Table). For both count and density, the linear model (or TL) provided a reasonable description of the relationship between spatial mean and spatial variance across states in a given census.

County abundance was tested against the spatial TL for each of 42 states, since the minimum requirements on the number of sampling unit (*n* ≥ 15) and mean-variance pairs (*N* ≥ 5) excluded some states. Using count, all but two states (ND and SD) were associated with significantly positive *b* with point estimates ranging from 2.04 (LA) to 5.54 (OK) (S40 Fig, S17 Table). Adj. *R*^2^ of the ols linear model ranged from 0.74 to 0.99. Among the ols quadratic models (ones with the smallest aicc), *e* (Eq 2) was not significantly different from zero in 29 of the 42 states, wasvsignificantly positive in four states (KS, NE, SC and WV), and wasvsignificantly negative in nine states (CO, LA, MD, ME, NJ, NM, UT, WA and WY) (S18 Table). Using density, *b* was significantly positive in all 42 states except ND and SD (S41 Fig, S17 Table). The point estimate of significant *b* ranged from 1.60 (MT) to 5.12 (OK) and ols linear regression adj. *R*^2^ ranged from 0.66 to 1.00. Among the ols quadratic models, *e* (Eq 2) was not significantly different from zero in 30 of the 42 states, significantly positive in two states (ID and NE), and significantly negative in ten states (CA, LA, MD, NV, NJ, NM, OR, UT, VA and WA) (S18 Table). Geographic distributions of the point estimate of *b* and the sign of *e* were similar to those when no minimum number of sampling units was applied (3a,c and S42 Figs).

County abundance was tested against the temporal TL for each of 36 states. Using count, all but one (RI) of the 36 states had significantly positive *b* in ols linear regressions, with values ranging from 2.04 (NJ) to 3.26 (SC) (S43 Fig, S19 Table). Adj. *R*^2^ of the ols linear regression ranged from 0.74 to 0.98. Among the ols quadratic regressions, *e* was not significantly different from zero in 32 of the 36 states, was significantly positive in one state (KS) and was significantly negative in three states (MD, MN and OH), indicating convex and concave log(mean)-log(variance) relationships (S20 Table). Using density, *b* from the ols linear regression was significantly positive in all 36 states except Rhode Island, with values ranging from 1.91 (NJ) to 3.25 (SC) (S44 Fig, S19 Table). Adj. *R*^2^ of the ols linear regression was between 0.76 and 0.98. Among the ols quadratic regressions, *e* was significantly positive in one state (KS) and significantly negative in five states (CA, IN, MD, OH and VA) (S20 Table). Geographic distributions of the point estimate of *b* and the sign of *e* with and without minimum number of sampling units requirement did not show substantial differences, except that a few states were omitted due to the lack of counties with at least 15 census records (3b,d and S45 Figs).

## **Ordinary least-squares regression diagnostics**

We implemented the same methods to test the appropriateness of ols regression assumptions. To summarize the following results, for all three types of TL, we did not find outliers and did not reject the normality and homoscedasticity of regression residuals in most censuses and states. For the spatial hierarchical TL and the temporal TL, we did not test for the spatial autocorrelation because we omitted states or counties when there were fewer than the minimum number of sampling units. For the spatial TL, we observed temporal autocorrelation in most states.

For the spatial hierarchical TL, using count and density, no test showed small p-value (< 0.05/23 = 0.0022) to reject the absence of outliers, normality (by the Shapiro-Wilk test, skewness test, and kurtosis test in the gvlma package in *R*) and homoscedasticity (by Breusch-Pagan test and homoscedasticity test in the gvlma package in R) of the ols linear regression residuals, with one exception (Orleans Territory was identified as an outlier in the 1810 census using count by the Bonferroni outlier test). The global test of the gvlma package did not show significant violation of the ols assumptions, regardless of count or density (S21 Table). The same results held for the ols quadratic regression residuals regardless of count or density, except for the existence of outliers in 1820 and 1840 for count (LA in both censuses), and 1840 for density (LA) (S22 Table). We did not test or measure the spatial autocorrelation among states in a given census using Moran's I, since the set of mean-variance pairs with the minimum sampling unit requirement did not include all states in the US. Having empty neighbors forbids Moran's I test.

For the spatial TL, the Durbin-Watson test showed significant first-order temporal autocorrelation in the ols linear regression residuals in all but ten and six of the 42 states, for count and density. Using count, outliers were present in nine states for count and seven states for density (S23 Table). Normality was not rejected except in VA for count. Homoscedasticity was rejected in VA (by the gvlma package in *R*) only. The global test rejected the appropriateness of the linear model assumptions in CO, ID, MD, NJ, NY, PA and VA for count, and in KY, LA, NJ, NY, NC and VA for density. For the ols quadratic regression, first order temporal autocorrelation was significant in 24 of the 42 states for count, and 32 of the 42 states for density (S24 Table). Outliers were present in ten states for count and ten states for density. Normality of the quadratic regression residuals was rejected in MT by the Shapiro-Wilk test and in VA by all three normality tests for count, and in none of the states for density. Homoscedasticity was rejected in CO by both homoscedasticity tests and in VA by the gvlma function only for count, and in none of the states for density. Global test did not indicate significant violation of the quadratic model assumptions except in IL, MT, NY, OR, PA and VA for count, and MN, NC, and OR for density.

For the temporal TL, outliers were present in the ols linear regression in seven and ten of the 36 states, for count and density (S25 Table). For the ols linear regression, normality was rejected in CA, IA, OH and PA for count, and in CA, IN, IA, OH and PA for density. Homoscedasticity was rejected in CA for density only. The global test rejected the appropriateness of the linear model assumptions in CA, IA and OH for count, and in CA, IN, IA, MD, OH and VA for density. For the ols quadratic regression, outliers were present in 13 states for count and seven states for density (S26 Table). Normality was rejected in CA and PA for count, and in IN, OH and PA for density. Homoscedasticity was rejected in NC and PA for count, and in KS and NE for density. The global test rejected the appropriateness of the quadratic model assumptions in CA, IL, IA, NE, NY and PA for count, and IN, IA, NE, NY, OH and PA for density. As for the spatial hierarchical TL, we did not use Moran's I to test the presence of the spatial autocorrelation in the ols regressions due to empty neighbor as a consequence of exclusion of counties that did not satisfy the minimum number of sampling units requirement.

## **Comparison with Taylor's law tested without sampling unit requirement**

For the spatial hierarchical TL, when the minimum number of sampling units to calculate a single mean-variance pair was set at 15 (*n* ≥ 15), ols linear regression yielded *b* systematically larger than the corresponding value without such restriction, regardless of count or density (S46 and S47 Figs), with very few exceptions. In a given census, states with fewer than 15 counties were mostly less populous than those with more counties. The phenomenon that *b* is smaller at lower mean values has been documented for moth data (Taylor and Woiwod 1982). A possible explanation of such a phenomenon for the US Census data is that populations in those states with small number of counties tend to be less aggregated in space than randomly distributed, which can lead to a Poisson distribution. The Poisson distribution has mean equal to variance, leading to *b* = 1 at small mean values and consequently dragging down the *b* value across the entire range of mean values.

On the other hand, for the spatial TL, we did not observe any systematic difference in *b* for the same state with or without the requirement of a minimum number of sampling units, regardless of count or density (S48 and S49 Figs). This is because within a state, the number of counties in a census was relatively stable over time and mostly above 15 (see *N* in Table 2 and S41 Table). Similarly, for the temporal TL, estimates of *b* showed similar values with or without the requirement of a minimum number of sampling units in most states, regardless of count and density (S50 and S51 Figs). The discrepancy in *b* was more substantial than that of the spatial TL and is not one-directional: *b* estimated with *n* ≥ 15 was greater or smaller than *b* estimated without the constraint on *n*, depending on the state.

## **Summary of the effects of required minimum sample size on estimates of b**

The effects of requiring at least 15 sampling units and at least five (mean, variance) pairs on point estimates of the TL slope *b* and the quadratic coefficient *e* of QTL differed little between count and density but differed substantially among spatial hierarchical, spatial, and temporal mean-variance relations. For the spatial hierarchical TL, imposing minimum data requirements systematically increased estimates of slope *b* (with rare exceptions) and likewise for the quadratic coefficient *e* of QTL. For the spatial TL, imposing minimum data requirements had almost negligible effect on estimates of slope *b* (again with rare exceptions) and likewise for the quadratic coefficient *e* of QTL. For the temporal TL, imposing minimum data requirements had considerable but no systematic effect on estimates of slope *b* (again with rare exceptions) and likewise for the quadratic coefficient *e* of QTL. To our knowledge, such a systematic analysis of the effect of minimum data requirements on estimates of *b* and *e* has not been done before.

# Supporting references

Breusch, T. S. and Pagan, A. R. (1979). A simple test for heteroscedasticity and random coefficient variation. *Econometrica* 47(5): 1287–1294.

Cliff, A. D. and Ord, J. K. (1981). Spatial processes: models and applications. London: Pion Ltd.

Cook, R. D. and Weisberg, S. (1982). Residuals and influence in regression. New York: Chapman and Hall.

Durbin, J. and Watson, G. S. (1950). Testing for serial correlation in least squares regression: I. *Biometrika* 37(3/4): 409–428.

Peña, E. A. and Slate, E. H. (2006). Global validation of linear model assumptions. *Journal of the American Statistical Association* 101(473): 341–354.

R Core Team (2018). R: A language and environment for statistical computing. R Foundation for Statistical Computing, Vienna, Austria. URL <https://www.R-project.org/>*.*

Royston, J. P. (1982). Algorithm AS 181: the W test for normality. *Journal of the Royal Statistical Society. Series C (Applied Statistics)* 31(2): 176–180.

Taylor, L. R. and Woiwod, I. P. (1982). Comparative synoptic dynamics. I. Relationships between inter-and intra-specific spatial and temporal variance/mean population parameters. *Journal of Animal Ecology* 51(3): 879-906.

Taylor, L. R., Perry, J. N., Woiwod, I. P., and Taylor, R. A. J. (1988). Specificity of the spatial power-law exponent in ecology and agriculture. *Nature* 332(6166): 721-722.

# Supporting figures and figure legends

S1 Fig

S1 Fig. Spatial variance of county population count against spatial mean of county population count on log-log scale across states within each census. Each circle represents one spatial mean and one spatial variance of county population count across all counties within a state in a given census. The solid and dashed lines are fitted linear and quadratic regression lines respectively.

S2 Fig S2 Fig. Spatial variance of county population count against spatial mean of county population count on log-log scale across censuses within each state. Circles and lines are defined in the legend of S1 Fig.

S3 Fig

 S3 Fig. Temporal variance of county population count against temporal mean of county population count on log-log scale across counties within each state. Each circle represents a temporal mean and a temporal variance of county population count across all available censuses for a given county. Lines are defined in the legend of S1 Fig.

S4 Fig

** S4 Fig. Regression coefficient estimates for TL and QTL using county population density. Panel titles are defined in the legend of Fig 1.

S5 Fig

 S5 Fig. Spatial variance of county population density against spatial mean of county population density on log-log scale across states within each census. Each circle represents one spatial mean and one spatial variance of county population density across all counties within a state in a given census. Lines are defined in the legend of S1 Fig.

S6 Fig

 S6 Fig. Time series of (a) the slope estimate of spatial hierarchical TL and (b) quadratic coefficient of spatial hierarchical QTL using county population density. Solid circles are point estimate and vertical lines denote the corresponding 95% confidence intervals.

S7 Fig

 S7 Fig. Spatial variance of county population density against spatial mean of county population density on log-log scale across censuses within each state. Circles and lines are defined in the legend of S5 Fig.

S8 Fig

 S8 Fig. Point estimates of the slope of (a) spatial TL and (b) temporal TL, and the sign of the quadratic coefficients of (c) spatial QTL and (d) temporal QTL by US states, using county population density. In (c) and (d), the sign of the quadratic coefficient was determined as in Fig 3. This figure is made with the free package fiftystater in R under the terms of the GNU General Public License as published by the Free Software Foundation, version 3.

S9 Fig

 S9 Fig. Temporal variance of county population density against temporal mean of county population density on log-log scale across counties within each state. Each circle represents a temporal mean and a temporal variance of county population density across all available censuses for a given county. Lines are defined in the legend of S1 Fig.

S10 Fig


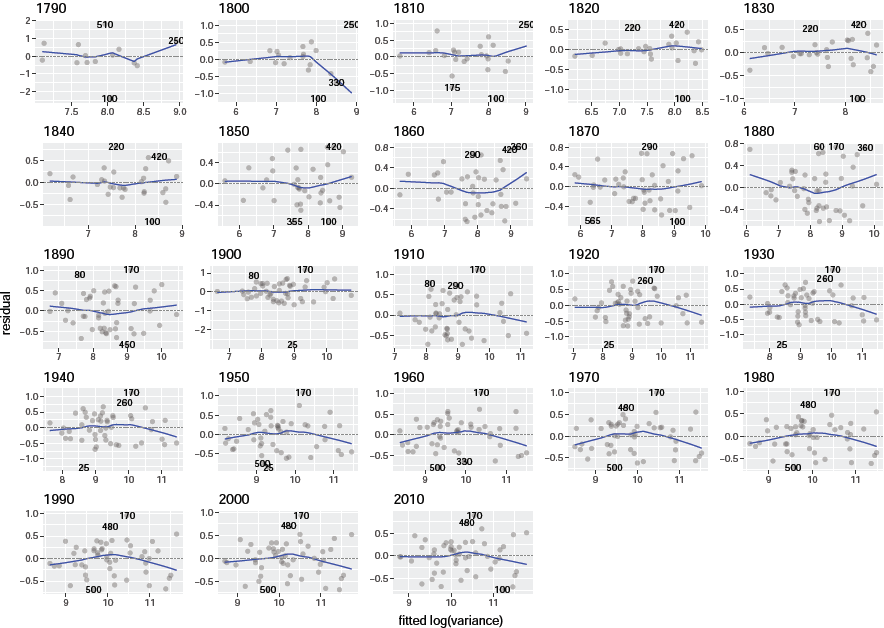


S10 Fig. Residuals against fitted values of the linear regression for log(spatial variance of county count) as a function of log(spatial mean of county count) across states in a given census. Dashed line is the reference line with a y-intercept of zero. The blue curve is the fitted loess smoothed curve showing the trend of the residuals.

S11 Fig


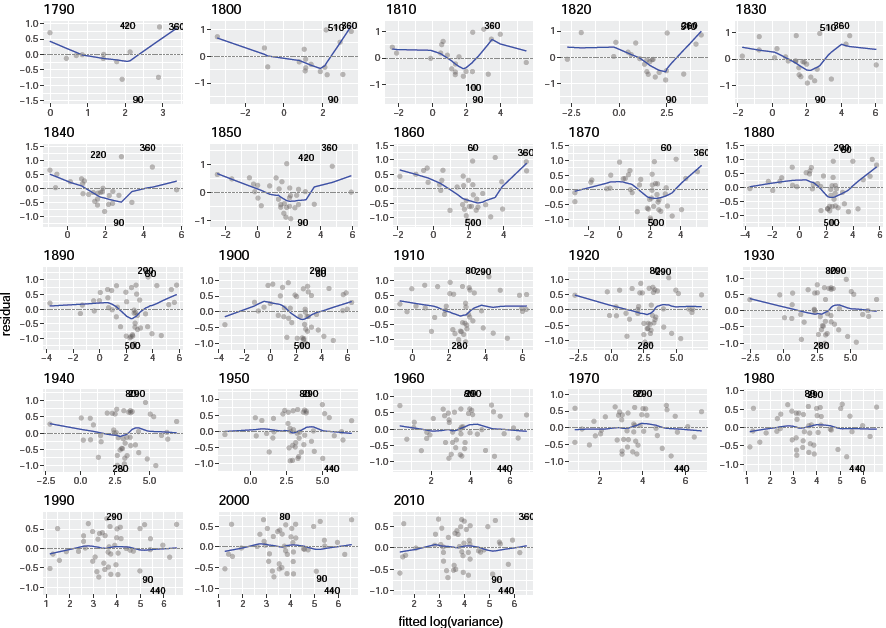


S11 Fig. Residuals against fitted values of the linear regression for log(spatial variance of county density) as a function of log(spatial mean of county density) across states in a given census. Dashed line and blue curve are defined in S10 Fig.

S12 Fig


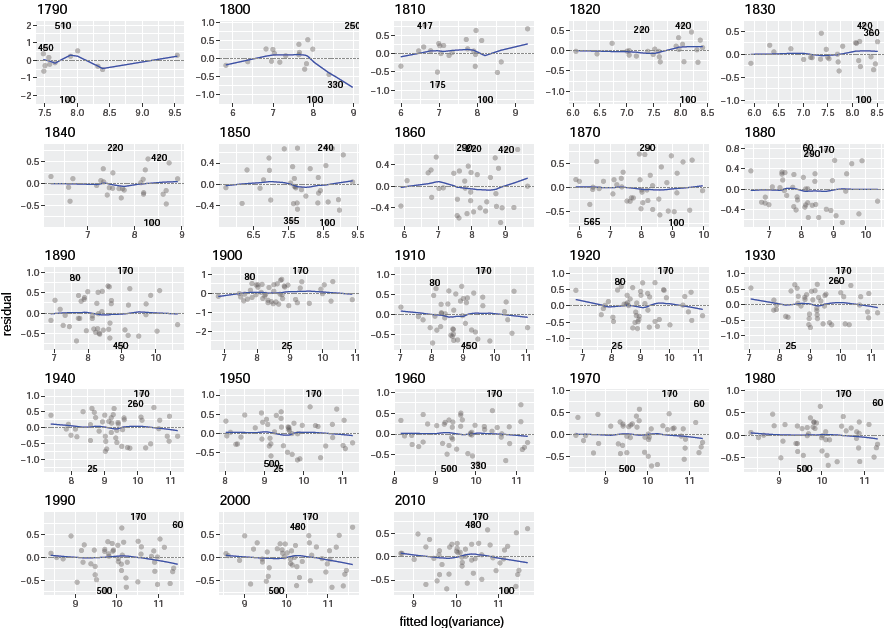


S12 Fig. Residuals against fitted values of the quadratic regression for log(spatial variance of county count) as a function of log(spatial mean of county count) across states in a given census. Dashed line and blue curve are defined in S10 Fig.

S13 Fig


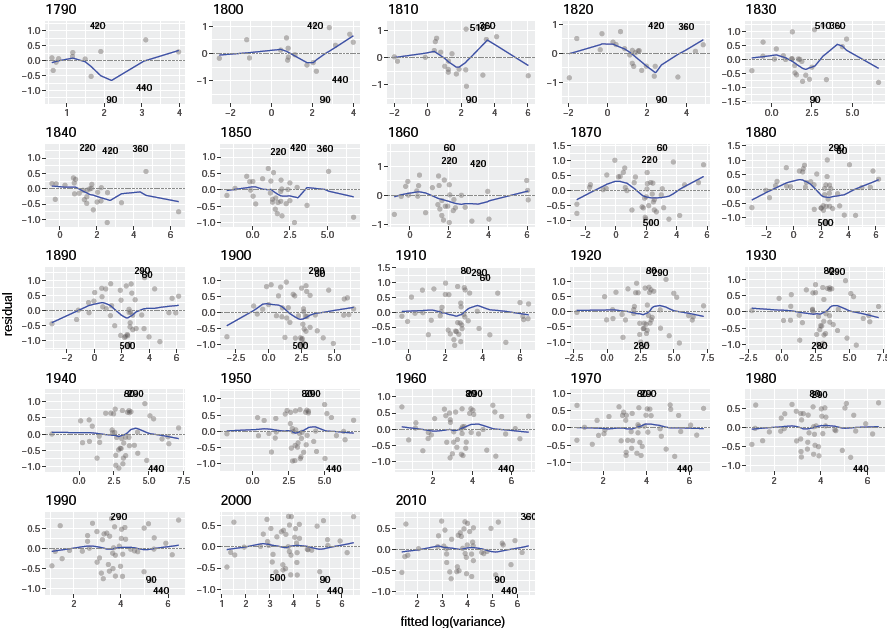


S13 Fig. Residuals against fitted values of the quadratic regression for log(spatial variance of county density) as a function of log(spatial mean of county density) across states in a given census. Dashed line and blue curve are defined in S10 Fig.

S14 Fig


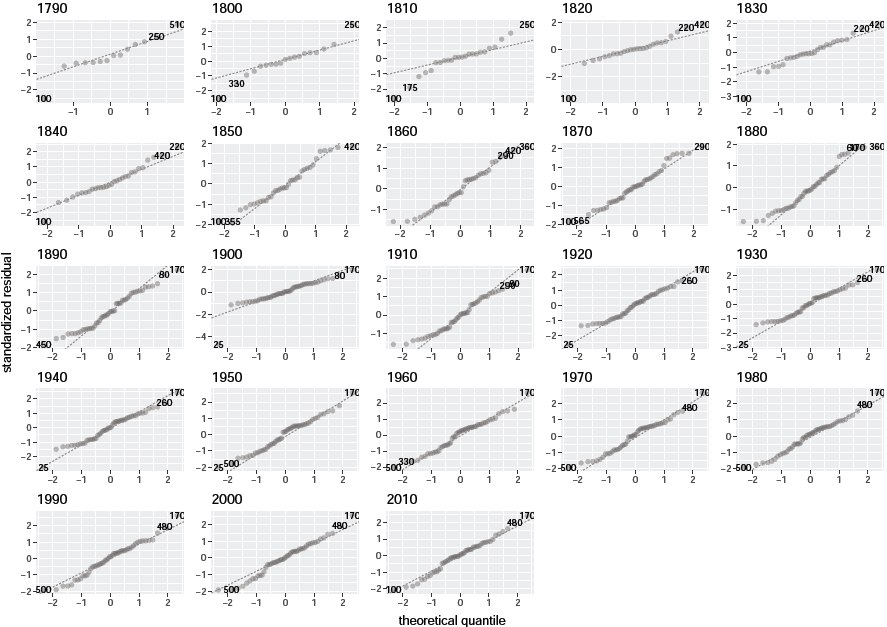


S14 Fig. Standardized residuals against their normal quantiles for the ols linear regression fitted to log(spatial variance of county count) as a function of log(spatial mean of county count) across states in a given census. Dashed line is the 45-degree line indicating agreement with the normal distribution.

S15 Fig


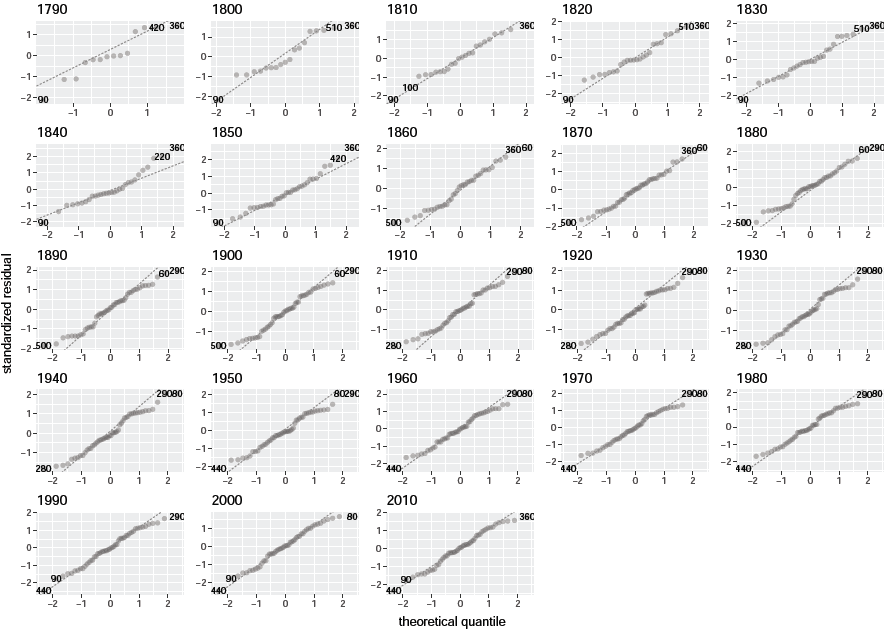


S15 Fig. Standardized residuals against their normal quantiles for the ols linear regression fitted to log(spatial variance of county density) as a function of log(spatial mean of county density) across states in a given census. Dashed line is defined in S14 Fig.

S16 Fig


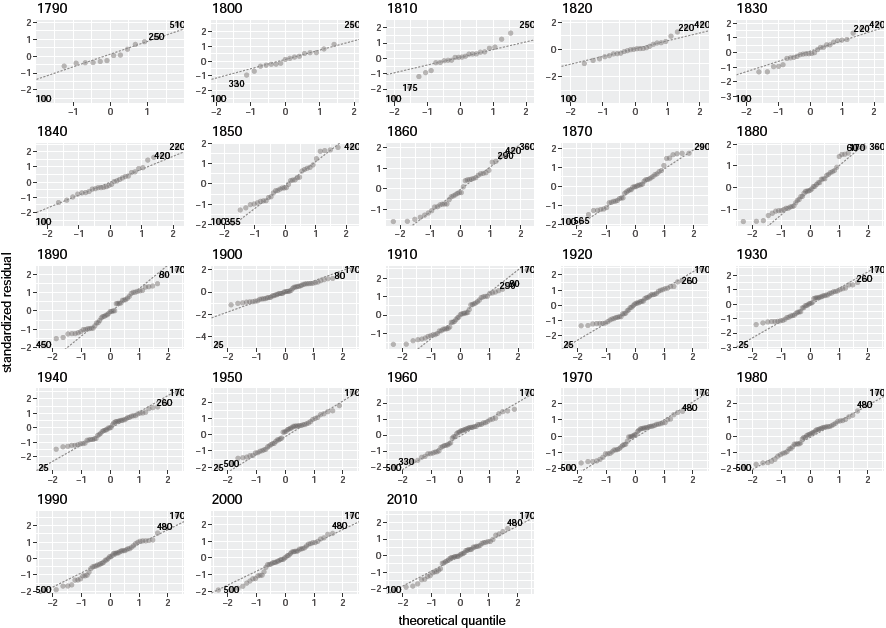


S16 Fig. Standardized residuals against their normal quantiles for the ols quadratic regression fitted to log(spatial variance of county count) as a function of log(spatial mean of county count) across states in a given census. Dashed line is defined in S14 Fig.

S17 Fig


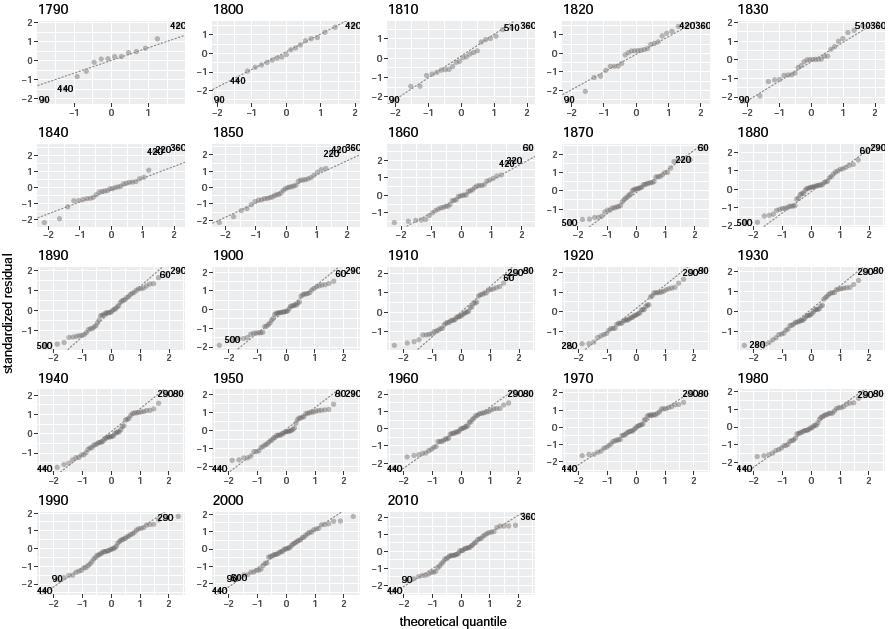


S17 Fig. Standardized residuals against their normal quantiles for the ols quadratic regression fitted to log(spatial variance of county density) as a function of log(spatial mean of county density) across states in a given census. Dashed line is defined in S14 Fig.

S18 Fig


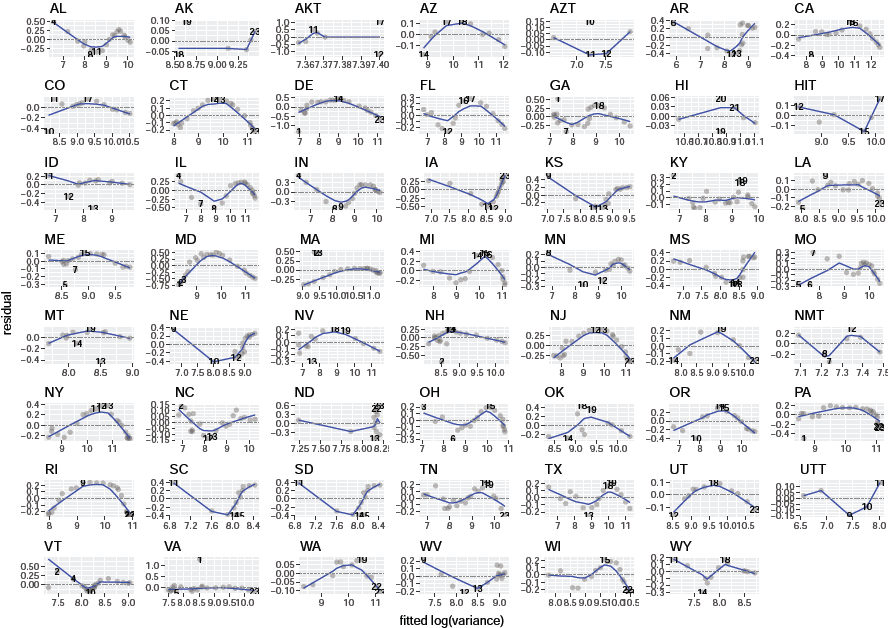


S18 Fig. Residuals against fitted value of the ols linear regression for log(spatial variance of county count) as a function of log(spatial mean of county count) across censuses for a given state. Dashed line and blue curve are defined in S10 Fig.

S19 Fig
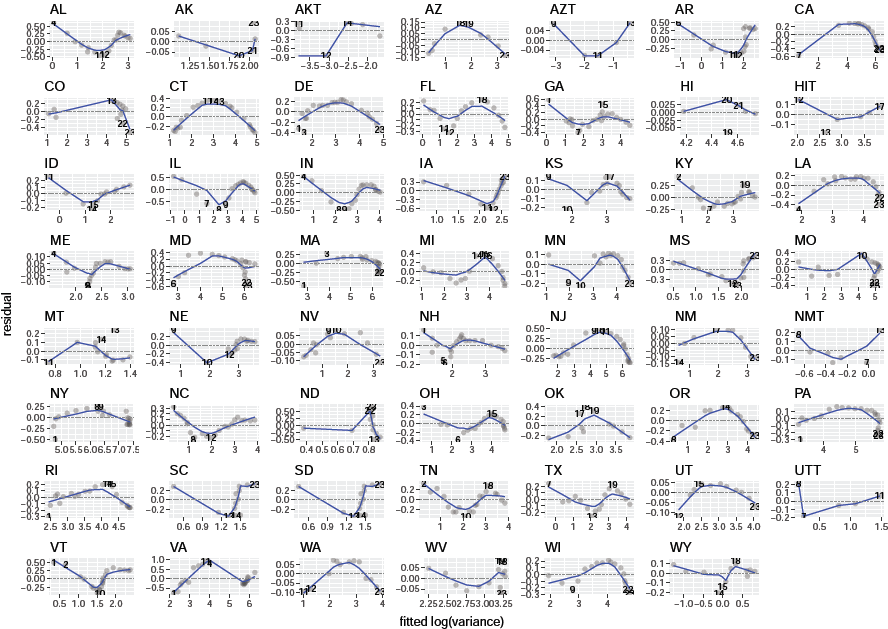


S19 Fig. Residuals against fitted value of the ols linear regression for log(spatial variance of county density) as a function of log(spatial mean of county density) across censuses for a given state. Dashed line and blue curve are defined in S10 Fig.

S20 Fig


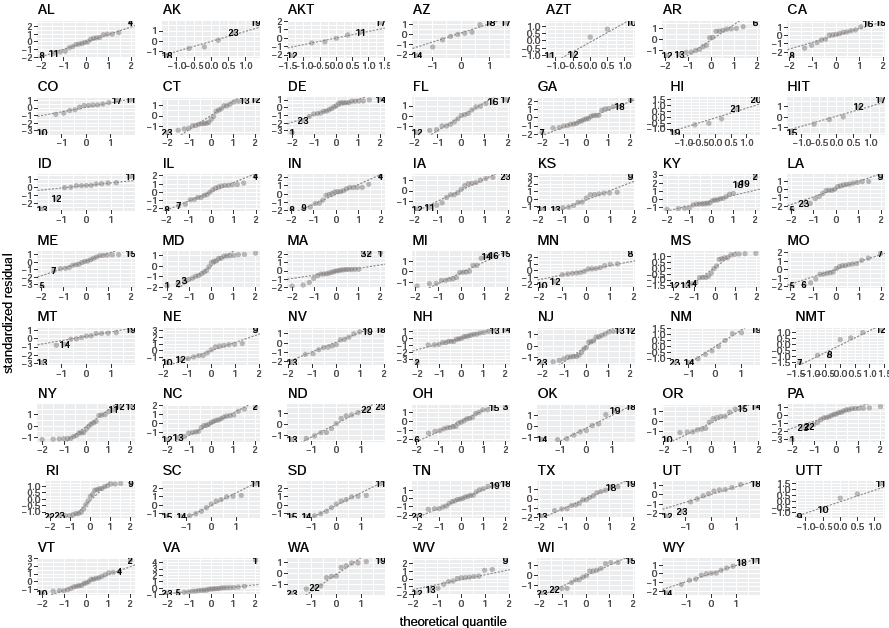


S20 Fig. Standardized residuals against their normal quantiles for the ols linear regression fitted to log(spatial variance of county count) as a function of log(spatial mean of county count) across censuses for a given state. Dashed line is defined in S14 Fig.

S21 Fig


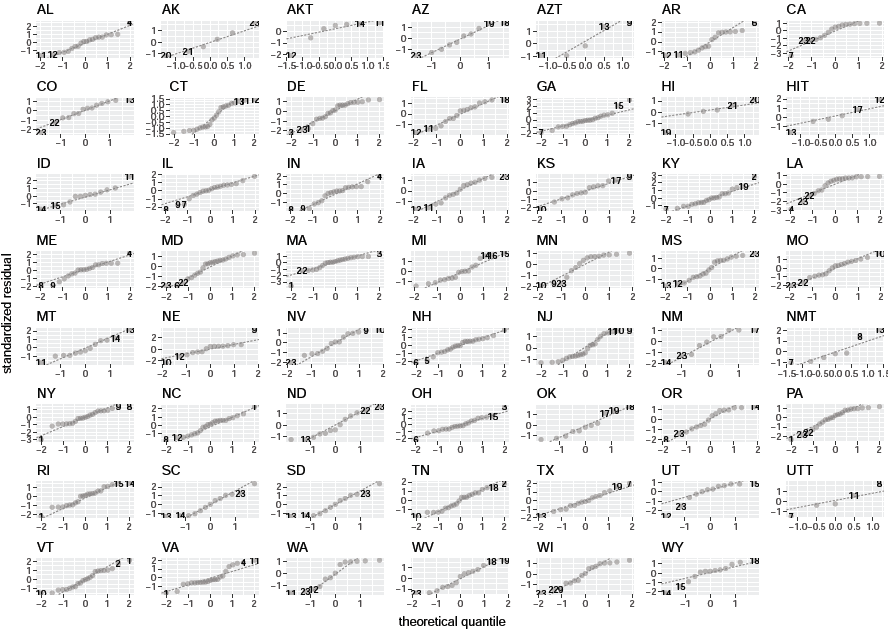


S21 Fig. Standardized residuals against their normal quantiles for the ols linear regression fitted to log(spatial variance of county density) as a function of log(spatial mean of county density) across censuses for a given state. Dashed line is defined in S14 Fig.

S22 Fig


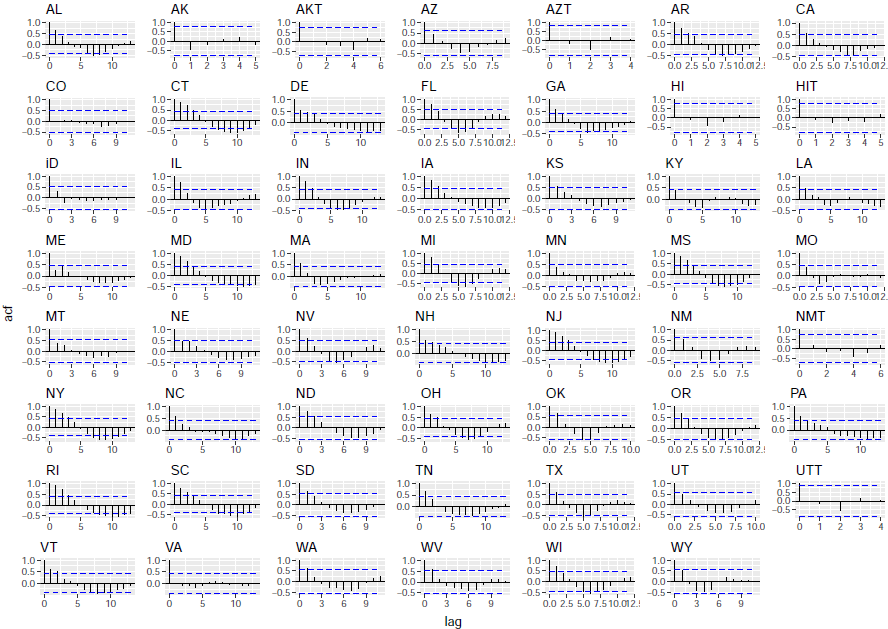


S22 Fig. Autocorrelation function against lag for the residuals of ols linear regression (up to lag 10) fitted to log(spatial variance of county count) as a function of log(spatial mean of county count) across censuses in a given state. Dashed horizontal lines show the critical correlation values. If any vertical line intersects with the dashed line then the temporal correlation at the corresponding lag is deemed significantly different from zero.

S23 Fig


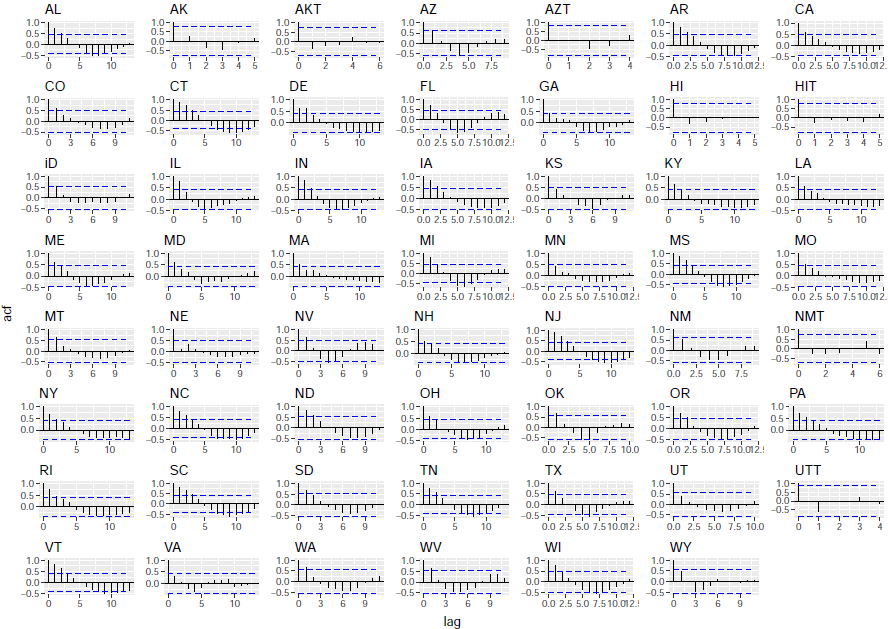


S23 Fig. Autocorrelation function against lag for the residuals of ols linear regression (up to lag 10) fitted to log(spatial variance of county density) as a function of log(spatial mean of county density) across censuses in a given state. Dashed horizontal lines are defined in S22 Fig.

S24 Fig
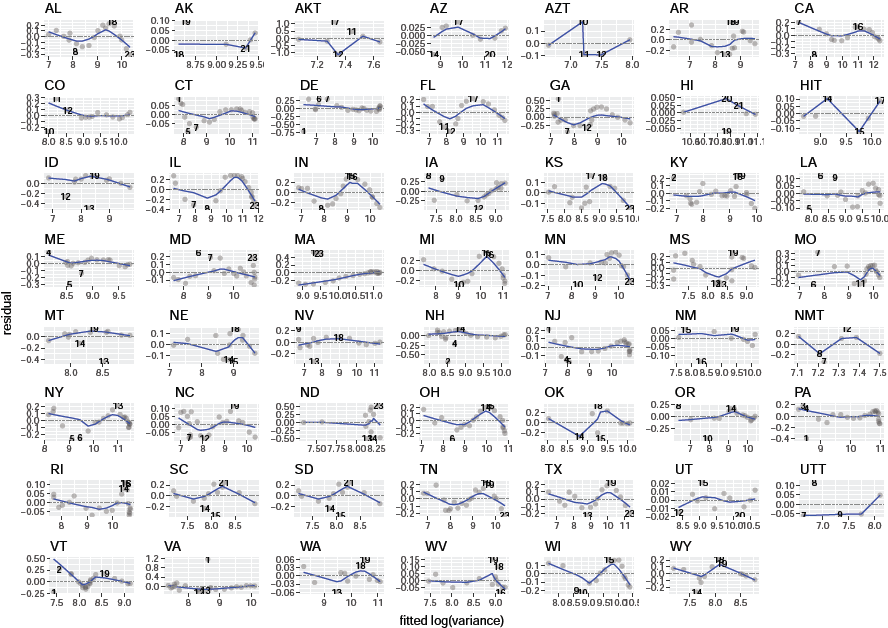


S24 Fig. Residuals against fitted value of the ols quadratic regression for log(spatial variance of county count) as a function of log(spatial mean of county count) across censuses for a given state. Dashed line and blue curve are defined in S10 Fig.

S25 Fig
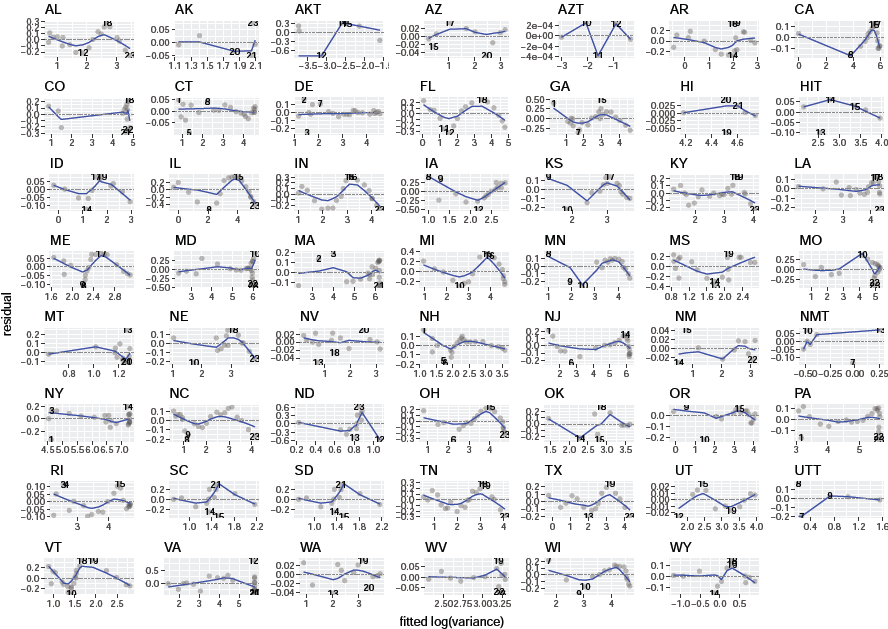


S25 Fig. Residuals against fitted value of the ols quadratic regression for log(spatial variance of county density) as a function of log(spatial mean of county density) across censuses for a given state. Dashed line and blue curve are defined in S10 Fig.

S26 Fig
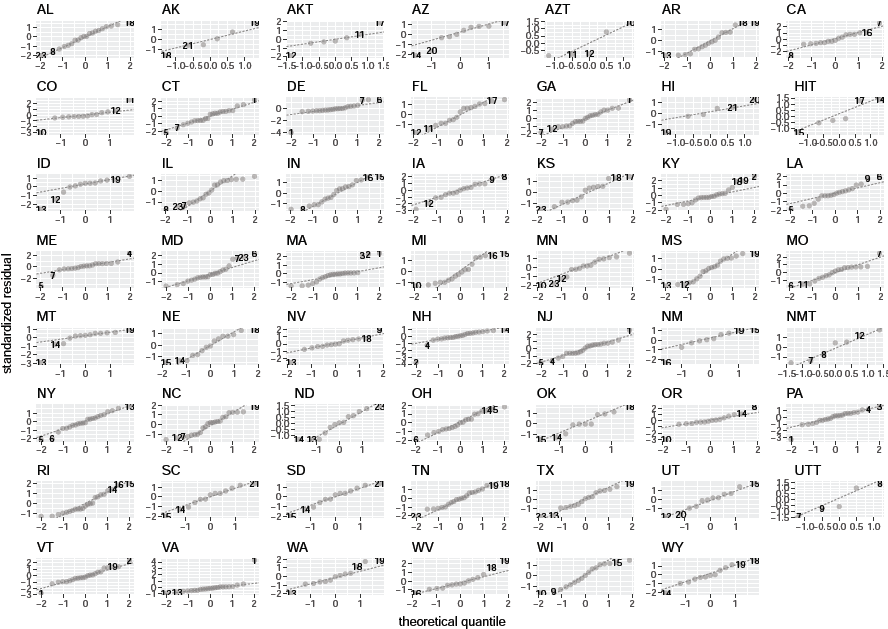


S26 Fig. Standardized residuals against their normal quantiles for the ols quadratic regression fitted to log(spatial variance of county count) as a function of log(spatial mean of county count) across censuses for a given state. Dashed line is defined in S14 Fig.

S27 Fig
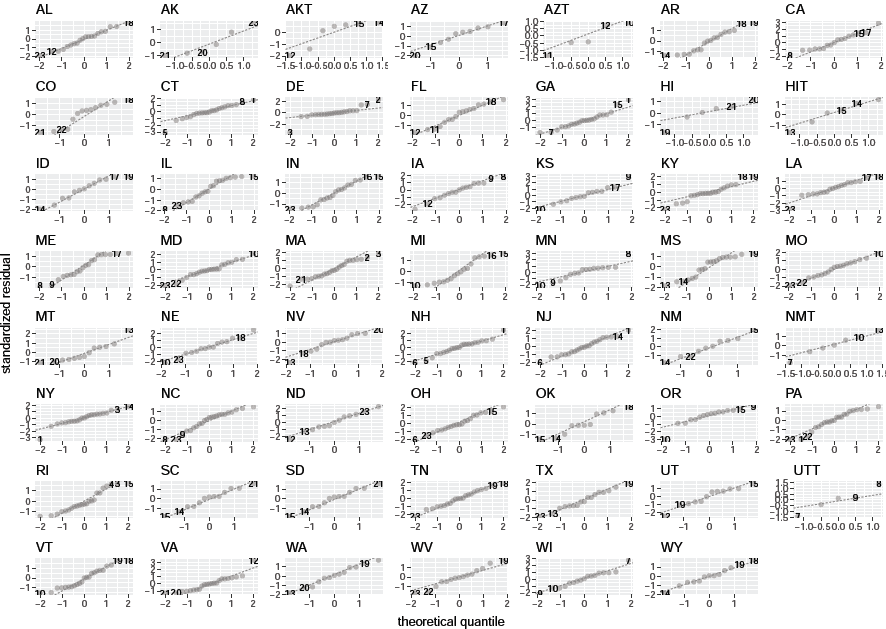


S27 Fig. Standardized residuals against their normal quantiles for the ols quadratic regression fitted to log(spatial variance of county density) as a function of log(spatial mean of county density) across censuses for a given state. Dashed line is defined in S14 Fig.

S28 Fig
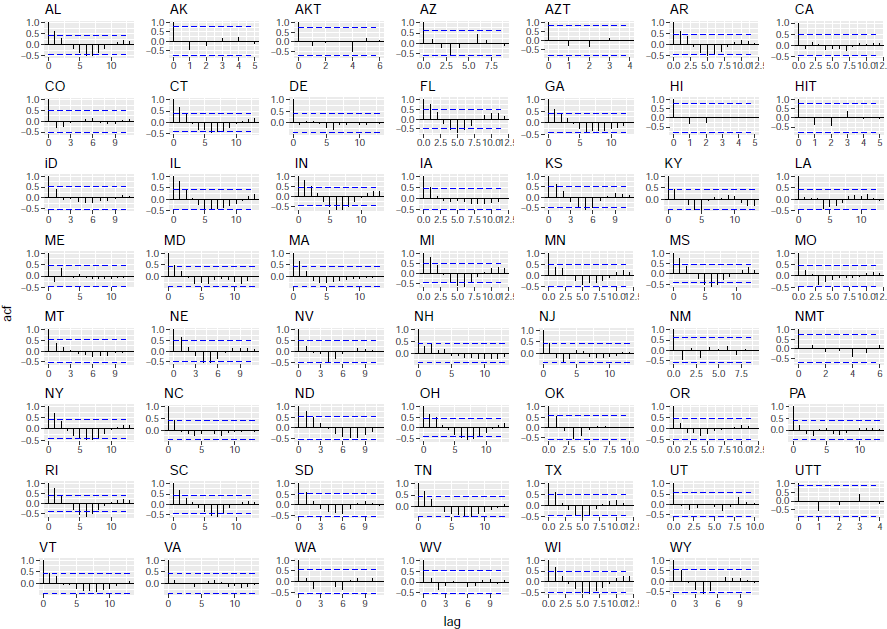
 S28 Fig. Autocorrelation function against lag for the residuals of ols quadratic regression (up to lag 10) fitted to log(spatial variance of county count) as a function of log(spatial mean of county count) across censuses in a given state. Dashed horizontal lines are defined in S22 Fig.

S29 Fig
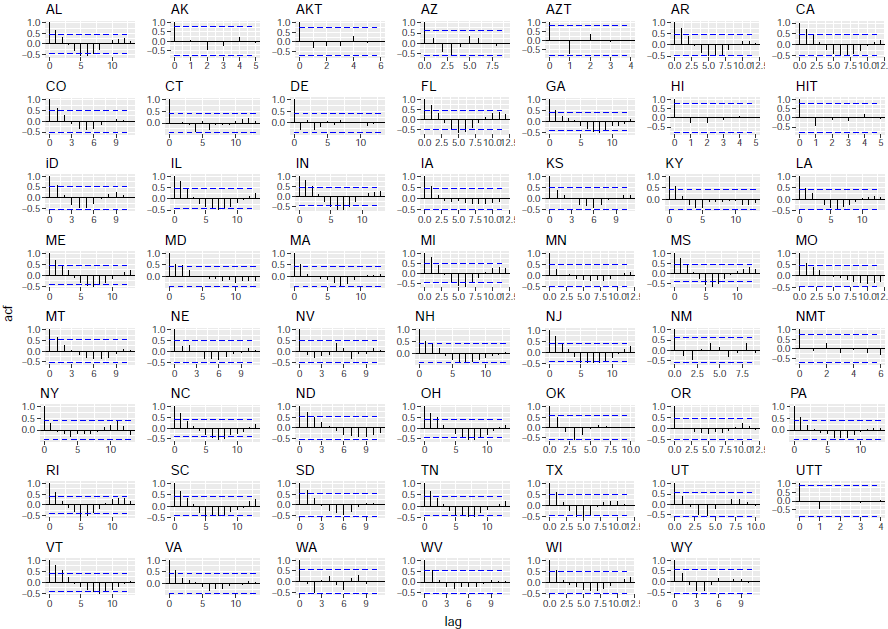
 S29 Fig. Autocorrelation function against lag for the residuals of ols quadratic regression (up to lag 10) fitted to log(spatial variance of county density) as a function of log(spatial mean of county density) across censuses in a given state. Dashed horizontal lines are defined in S22 Fig.

S30 Fig


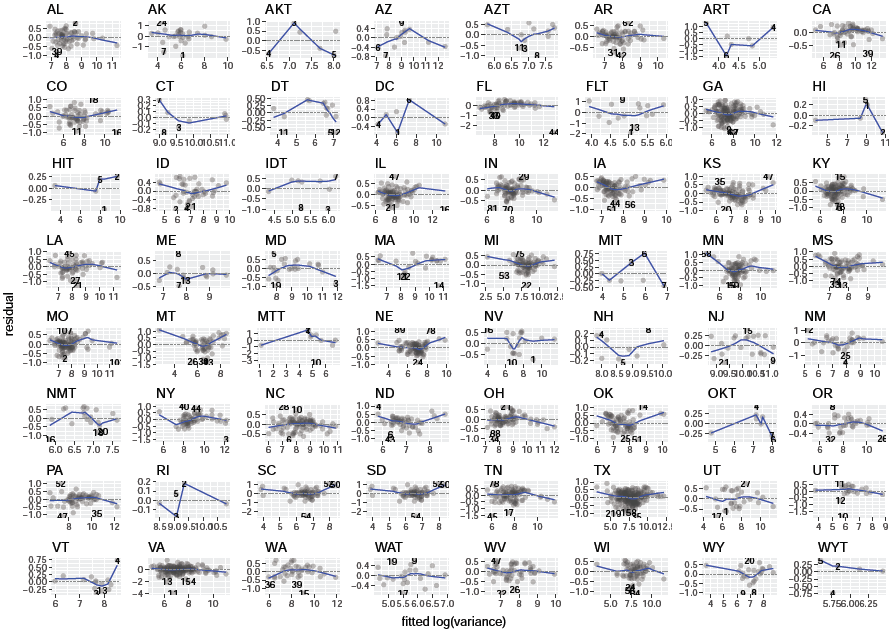


S30 Fig. Residuals against fitted value of the ols linear regression for log(temporal variance of county count) as a function of log(temporal mean of county count) across counties for a given state. Dashed line and blue curve are defined in S10 Fig.

S31 Fig
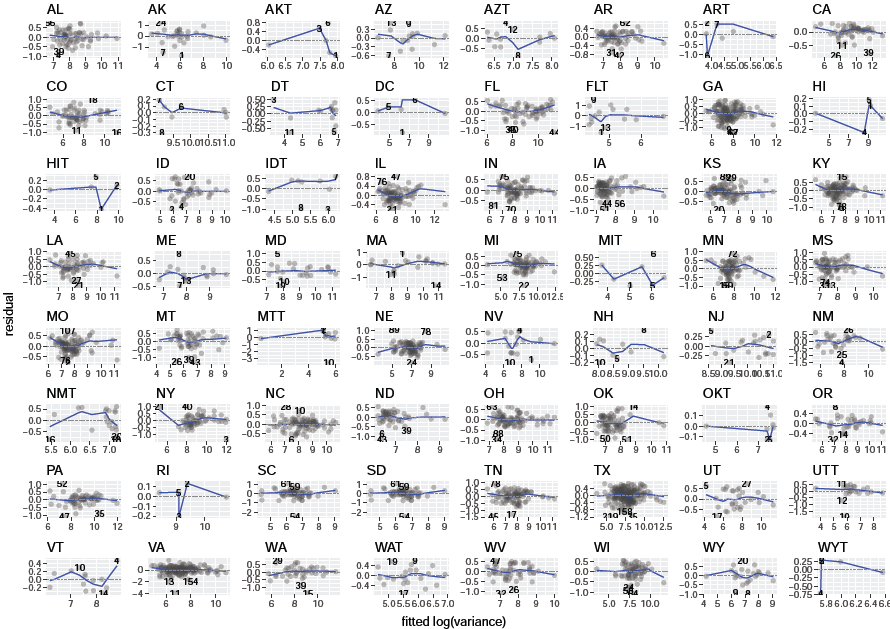


S31 Fig. Residuals against fitted value of the ols quadratic regression for log(temporal variance of county count) as a function of log(temporal mean of county count) across counties for a given state. Dashed line and blue curve are defined in S10 Fig.

S32 Fig
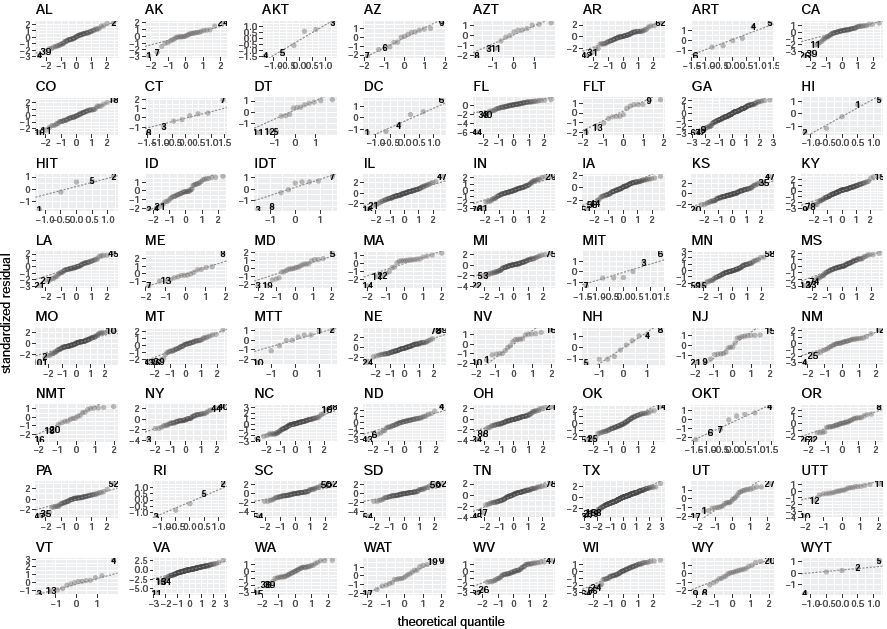
 S32 Fig. Standardized residuals against their normal quantiles for the ols linear regression fitted to log(temporal variance of county count) as a function of log(temporal mean of county count) across counties for a given state. Dashed line is defined in S14 Fig.

S33 Fig
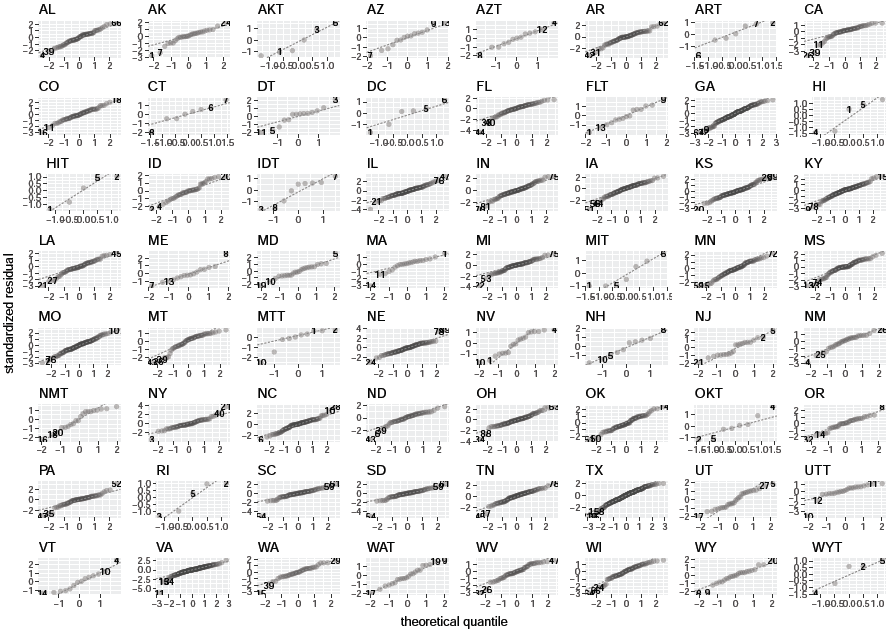
 S33 Fig. Standardized residuals against their normal quantiles for the ols quadratic regression fitted to log(temporal variance of county count) as a function of log(temporal mean of county count) across counties for a given state. Dashed line is defined in S14 Fig.

S34 Fig
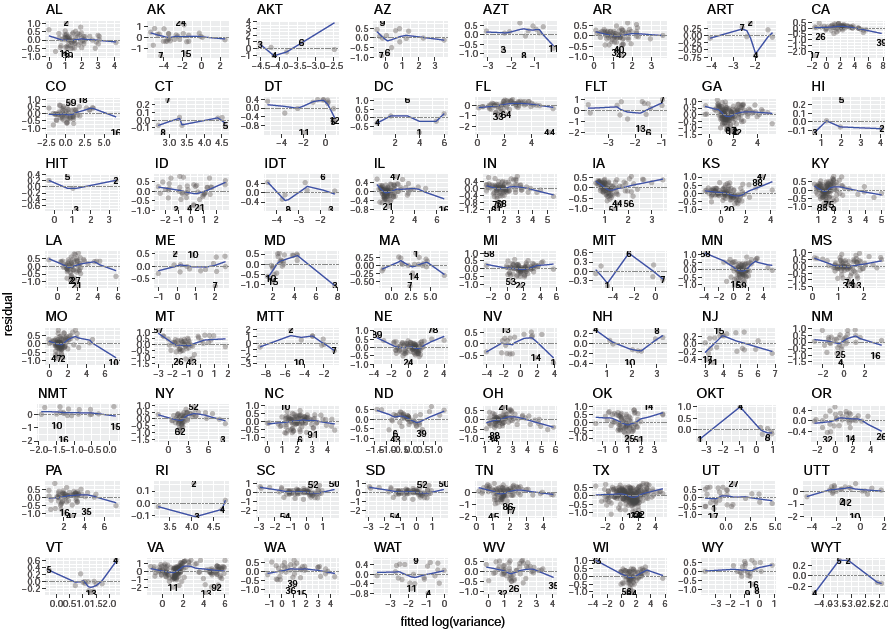
 S34 Fig. Residuals against fitted value of the ols linear regression for log(temporal variance of county density) as a function of log(temporal mean of county density) across counties for a given state. Dashed line and blue curve are defined in S10 Fig.

S35 Fig
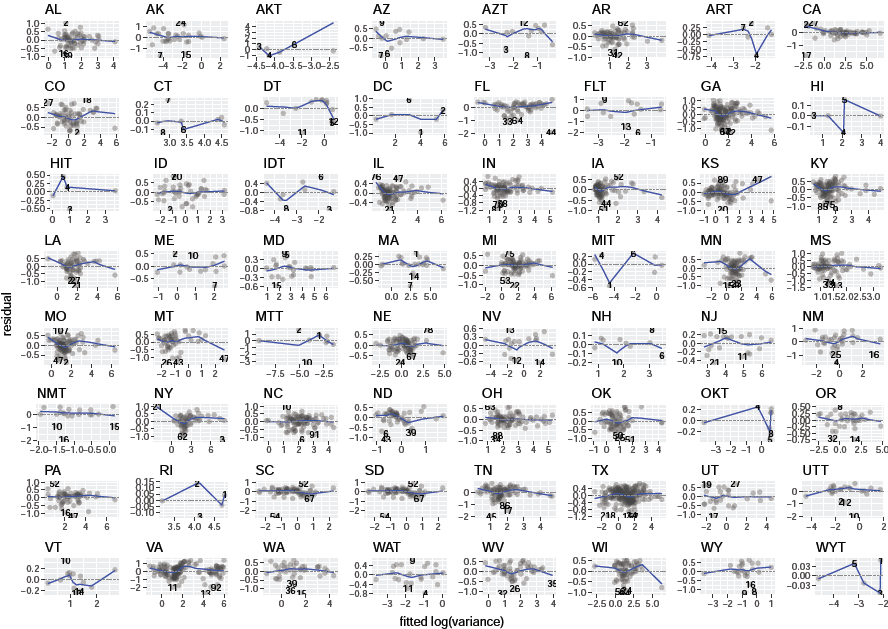
 S35 Fig. Residuals against fitted value of the ols quadratic regression for log(temporal variance of county density) as a function of log(temporal mean of county density) across counties for a given state. Dashed line and blue curve are defined in S10 Fig.

S36 Fig
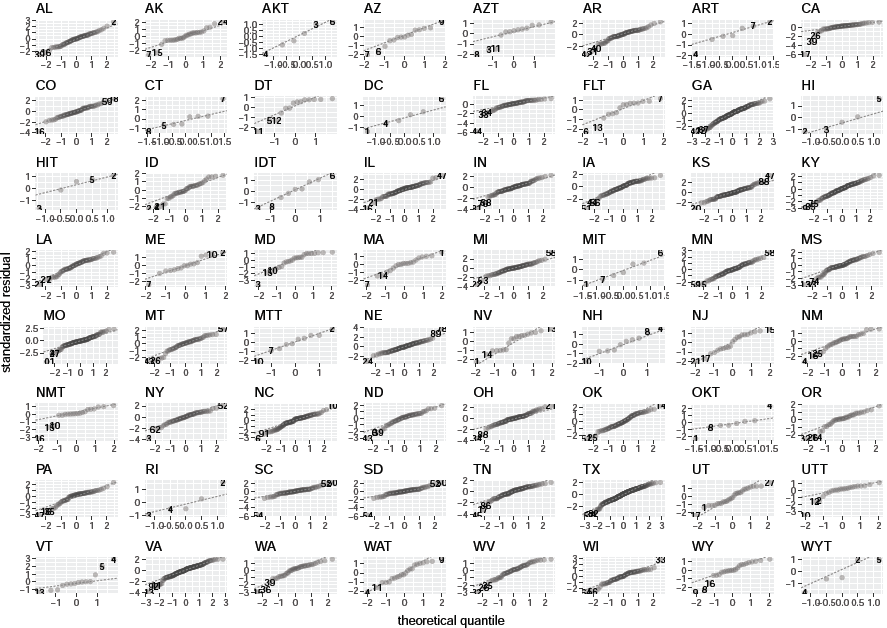
 S36 Fig. Standardized residuals against their normal quantiles for the ols linear regression fitted to log(temporal variance of county density) as a function of log(temporal mean of county density) across counties for a given state. Dashed line is defined in S14 Fig.

S37 Fig
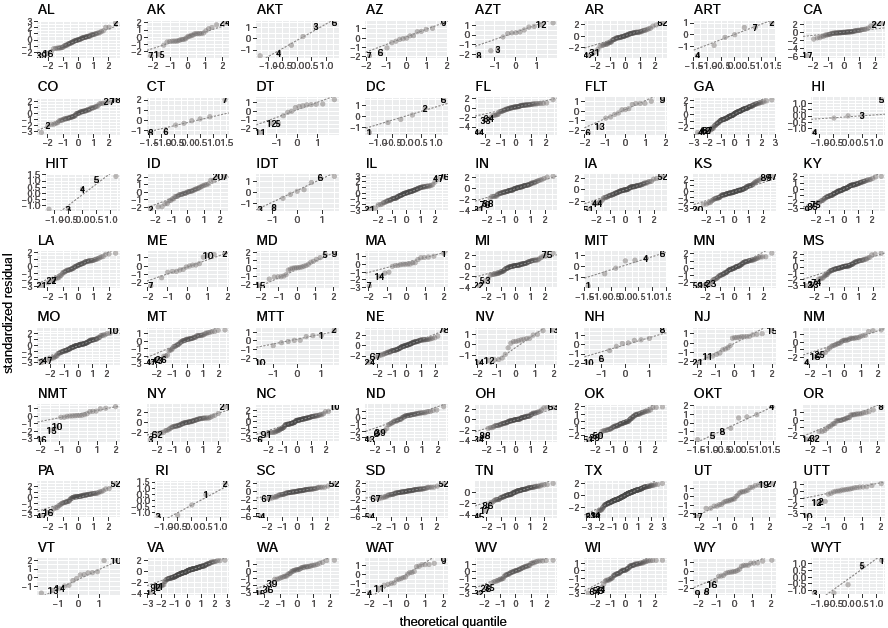
 S37 Fig. Standardized residuals against their normal quantiles for the ols quadratic regression fitted to log(temporal variance of county density) as a function of log(temporal mean of county density) across counties for a given state. Dashed line is defined in S14 Fig.

S38 Fig


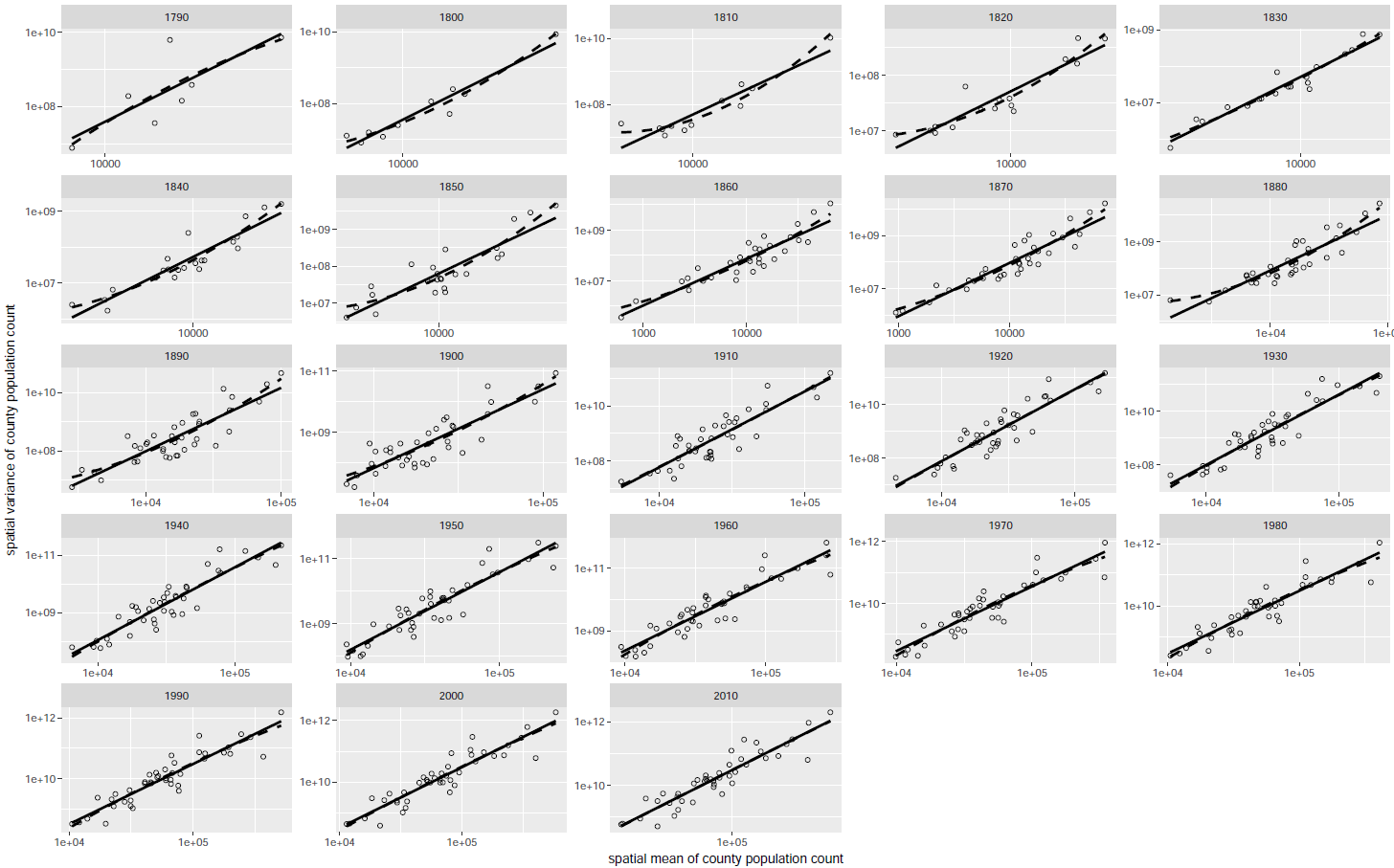


S38 Fig. Spatial variance of county count against spatial mean of county count across states in each census. Each mean-variance pair (a single circle) is calculated from at least 15 counties with finite non-zero population count in a given census and state. Solid and dashed lines are fitted ols linear and ols quadratic regression lines.

S39 Fig
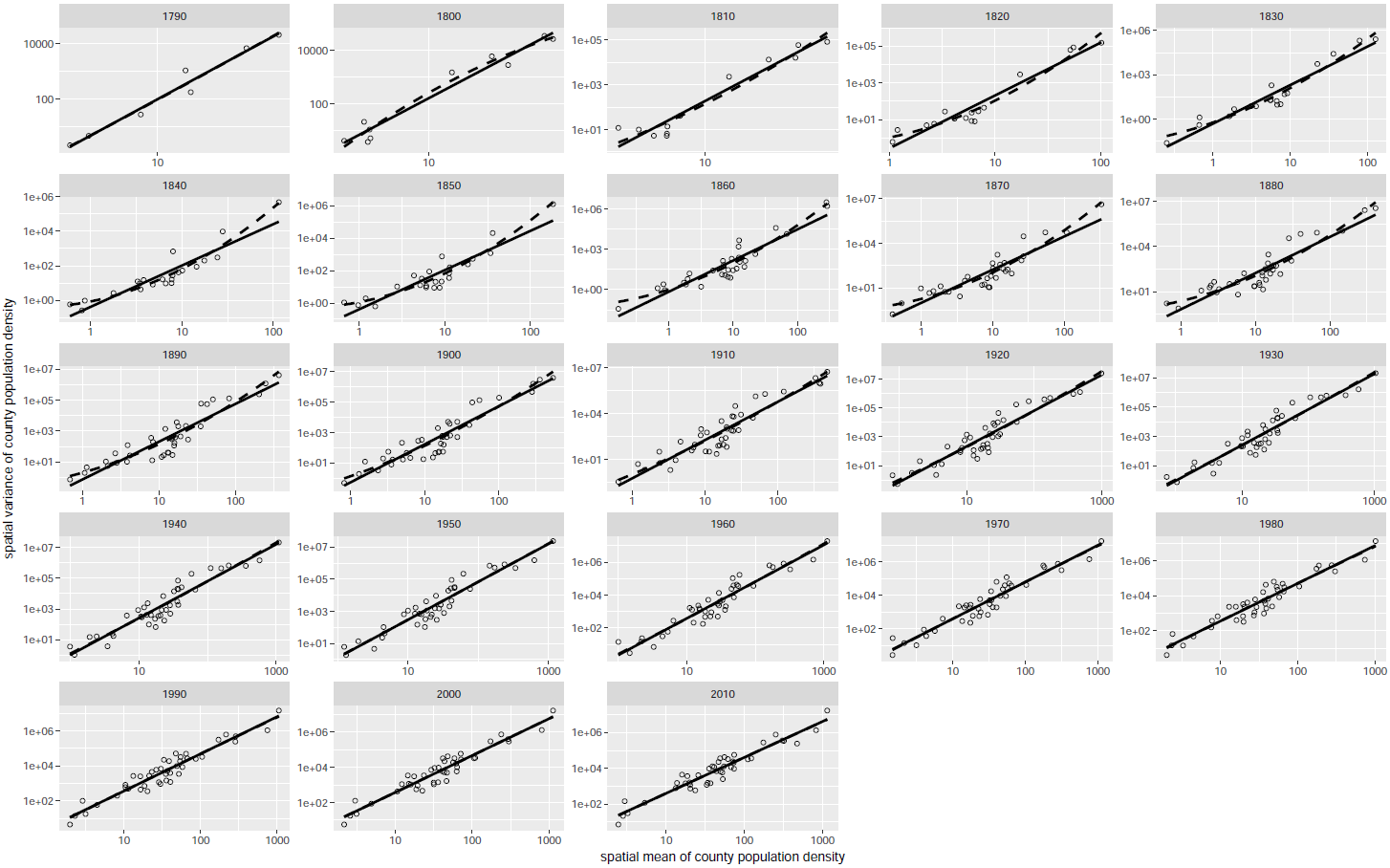
 S39 Fig. Spatial variance of county density against spatial mean of county density across states in each census. Each mean-variance pair (a single circle) is calculated from at least 15 counties with finite population density in a given census and state. Solid and dashed lines are fitted ols linear and ols quadratic regression lines.

S40 Fig
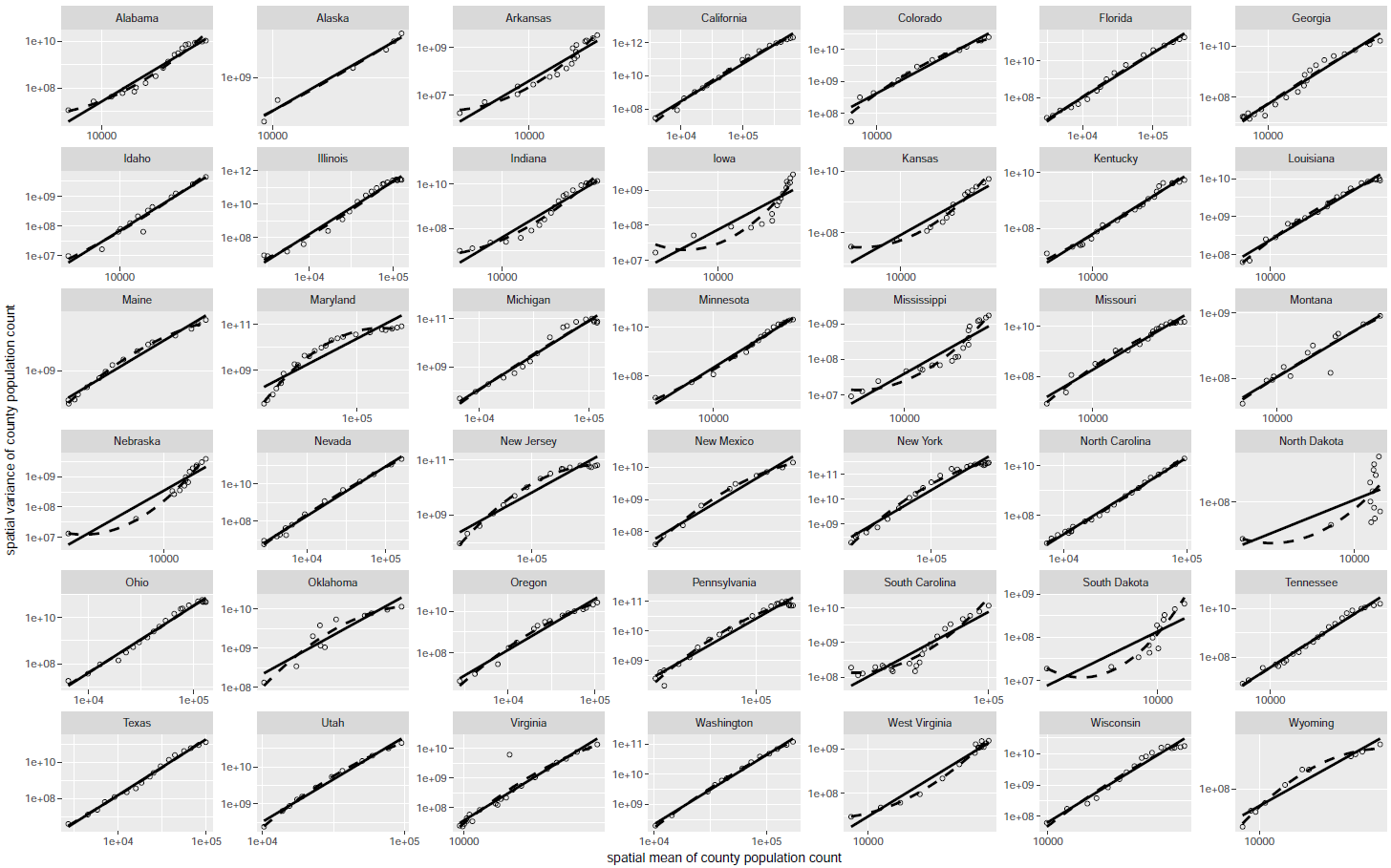
 S40 Fig. Spatial variance of county count against spatial mean of county count across censuses in each state that occurs in at least 5 censuses. Each mean-variance pair (a single circle) is calculated from at least 15 counties with finite non-zero population count in a given state and census. Solid and dashed lines are fitted ols linear and ols quadratic regression lines.

S41 Fig
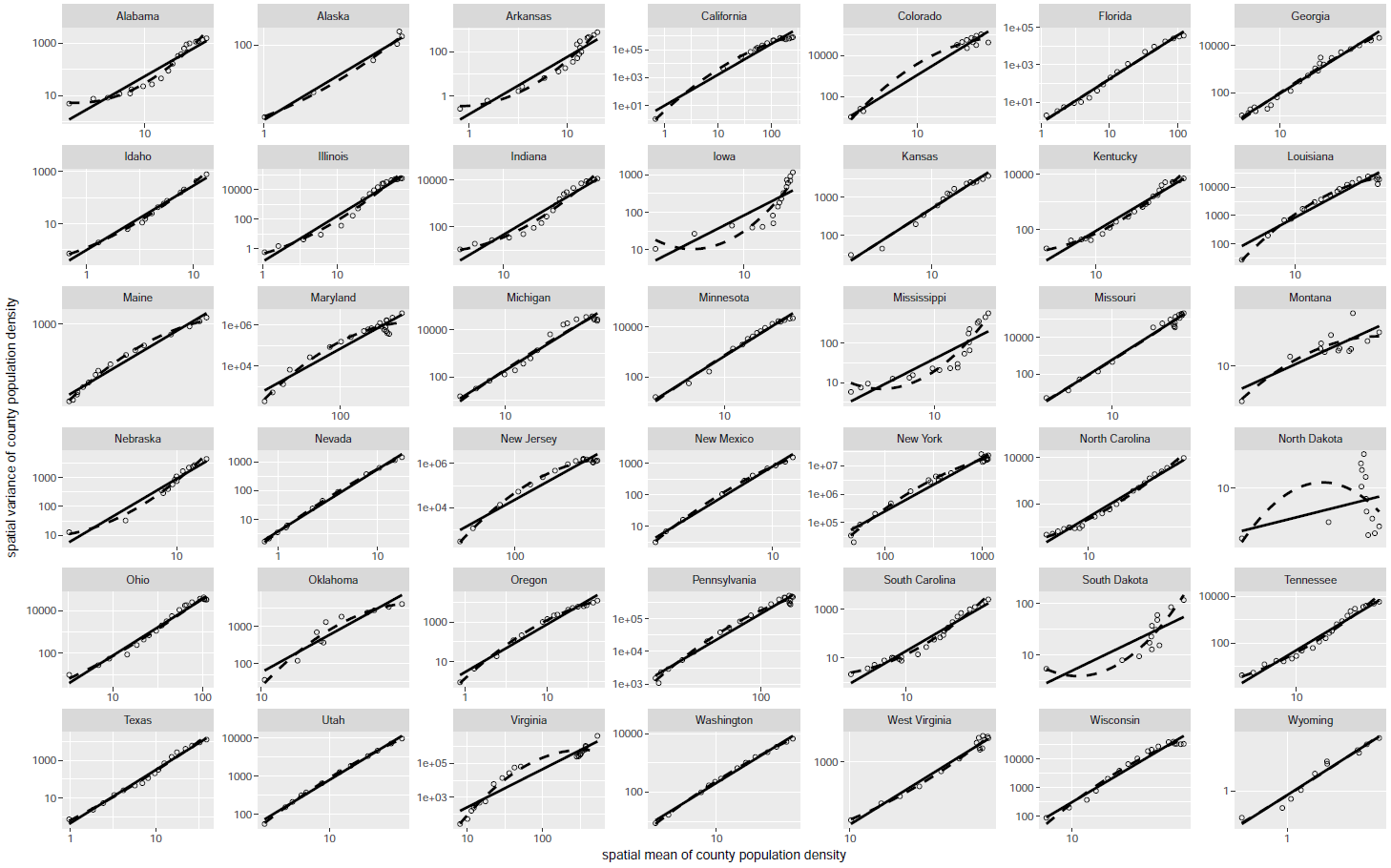


S41 Fig. Spatial variance of county density against spatial mean of county density across censuses in each state that occurs in at least 5 censuses. Each mean-variance pair (a single circle) is calculated from at least 15 counties with finite non-zero population count in a given state and census. Solid and dashed lines are fitted ols linear and ols quadratic regression lines.

S42 Fig
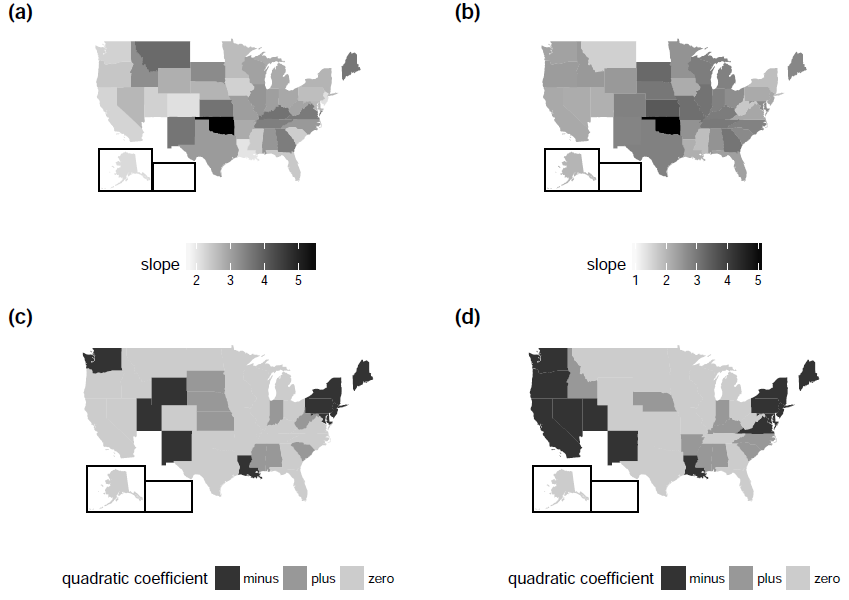
 S42 Fig. Point estimates of the slope ((a) and (b)) of ols linear regressions and the sign of the quadratic coefficient ((c) and (d)) of ols quadratic regressions for spatial TL for each state that occurs in at least 5 censuses, using count ((a) and (c)) and density ((b) and (d)). In (c) and (d), the sign of the quadratic coefficient is determined by the corresponding point estimate (minus or plus) and the p-value (significant difference from zero if p-value < 0.0012 = 0.05/42). The number of counties (with finite abundance) for each combination of state and census is at least 15. White regions mean the states therein are omitted due to the lack of counties satisfying the minimum number of sampling units requirement. This figure is made with the free package fiftystater in R under the terms of the GNU General Public License as published by the Free Software Foundation, version 3.

S43 Fig
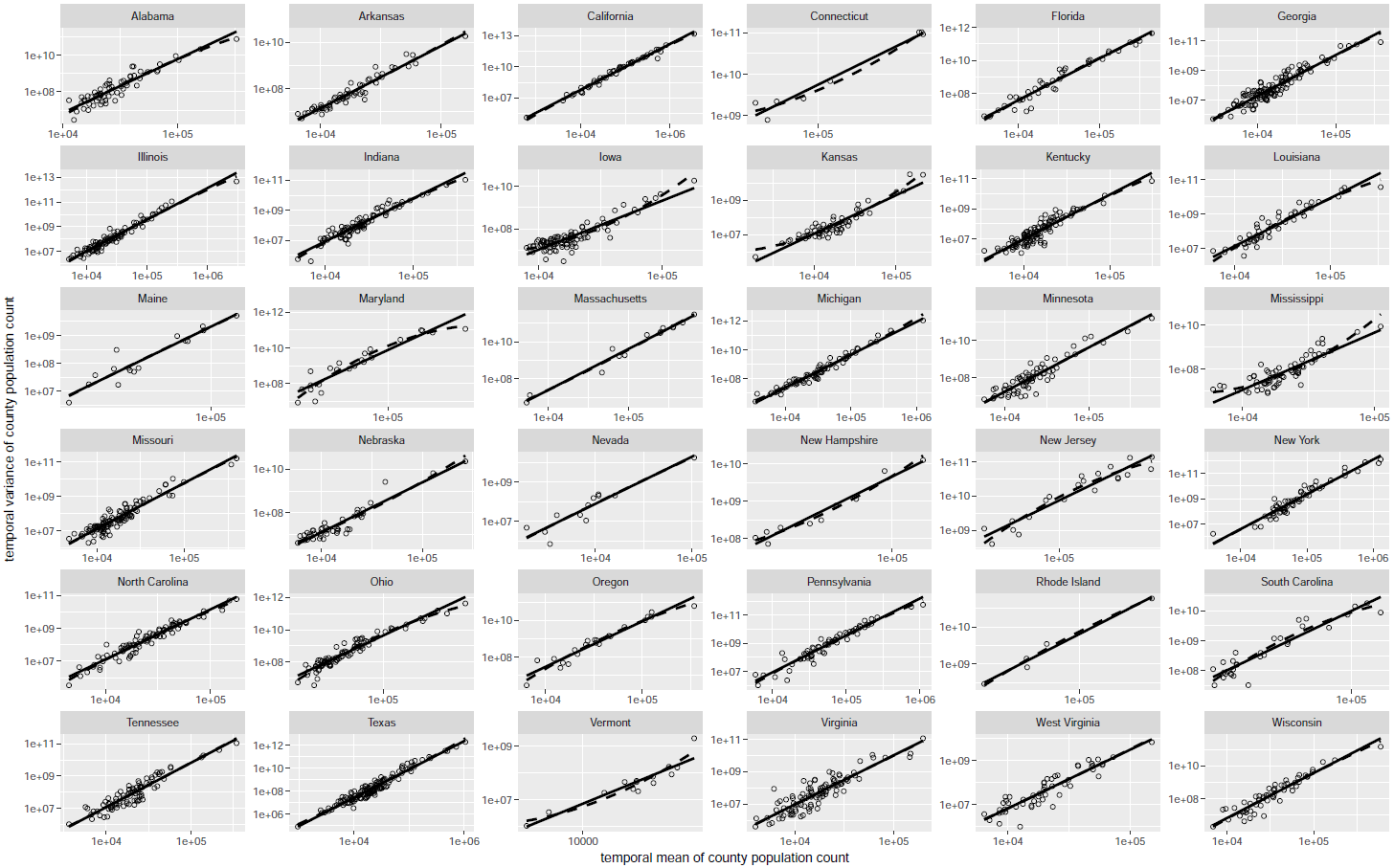
 S43 Fig. Temporal variance of county count against temporal mean of county count across counties in each of 36 states. Each mean-variance pair (a single circle) is calculated from at least 15 censuses with finite non-zero population count in a given state and county. Solid and dashed lines are fitted ols linear and ols quadratic regression lines.

S44 Fig


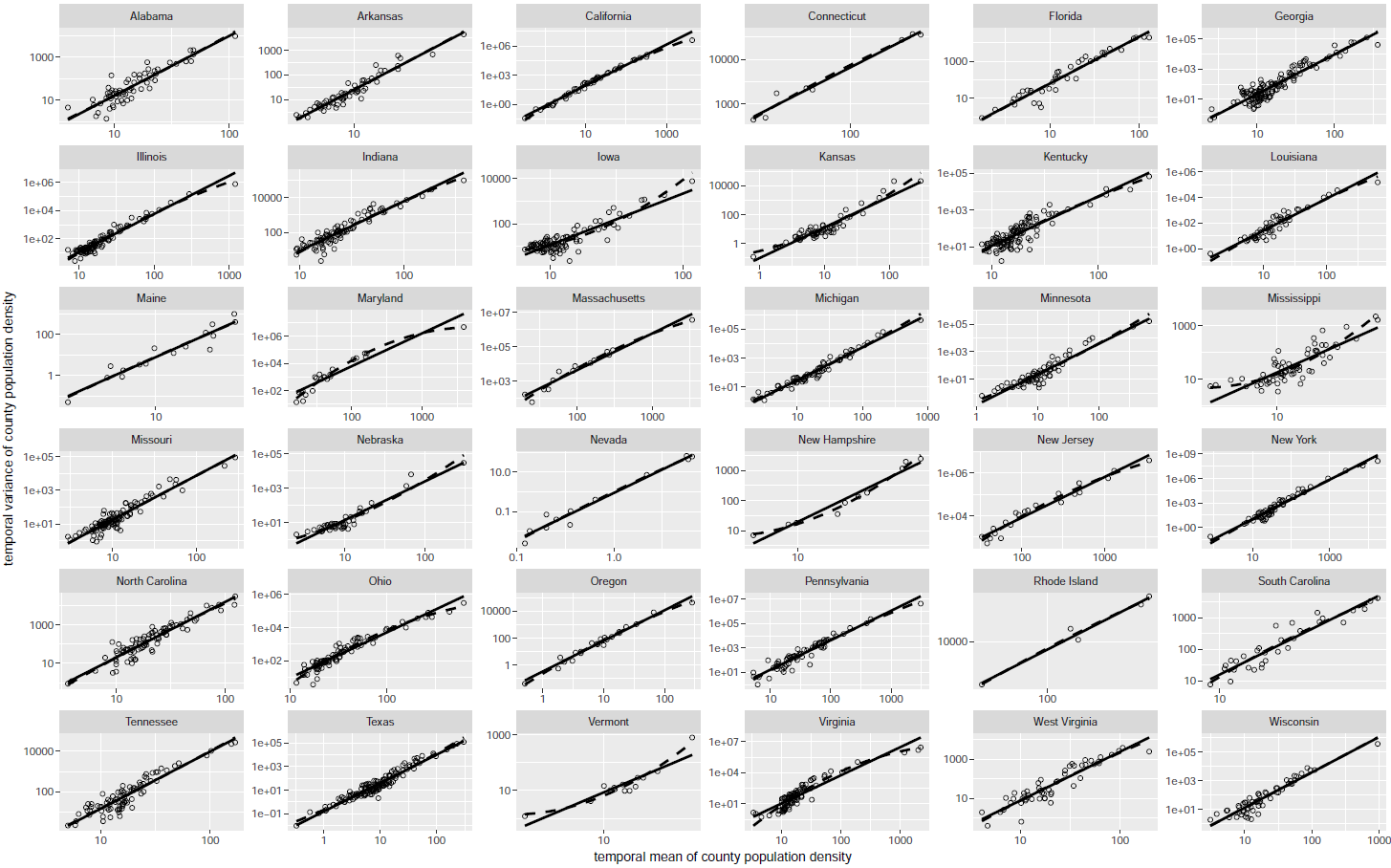
 S44 Fig. Temporal variance of county density against temporal mean of county density across counties in each of 36 states. Each mean-variance pair (a single circle) is calculated from at least 15 censuses with finite population density in a given state and county Solid and dashed lines are fitted ols linear and ols quadratic regression lines.

S45 Fig
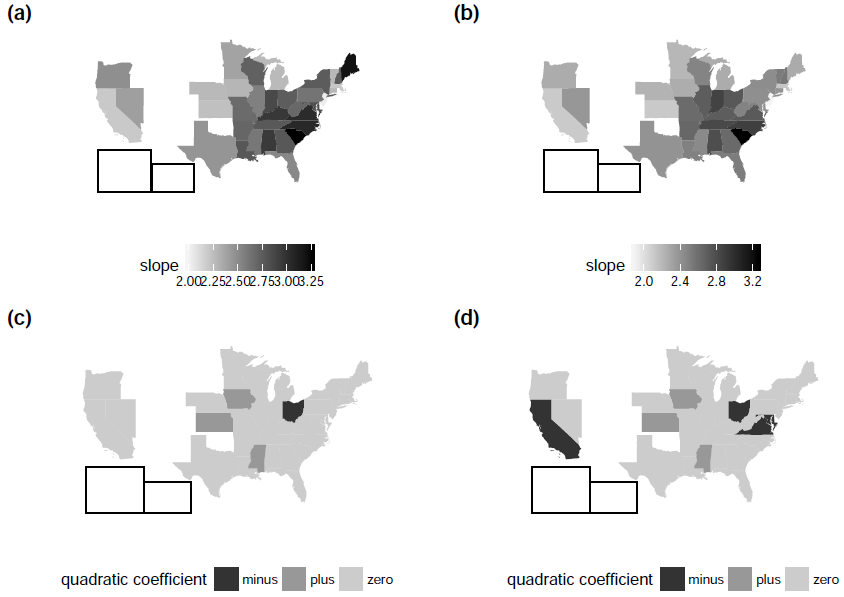
 S45 Fig. Point estimates of the slope ((a) and (b)) of ols linear regressions and the sign of the quadratic coefficient ((c) and (d)) of ols quadratic regressions for spatial TL for each state, using count ((a) and (c)) and density ((b) and (d)). In (c) and (d), the sign of the quadratic coefficient is determined by the corresponding point estimate (minus or plus) and the p-value (significant difference from zero if p-value < 0.0014 = 0.05/36). The number of censuses (with finite abundance) for each combination of state and county is at least 15. White regions mean the states therein are omitted due to the lack of counties satisfying the minimum number of sampling units requirement. This figure is made with the free package fiftystater in R under the terms of the GNU General Public License as published by the Free Software Foundation, version 3.

S46 Fig


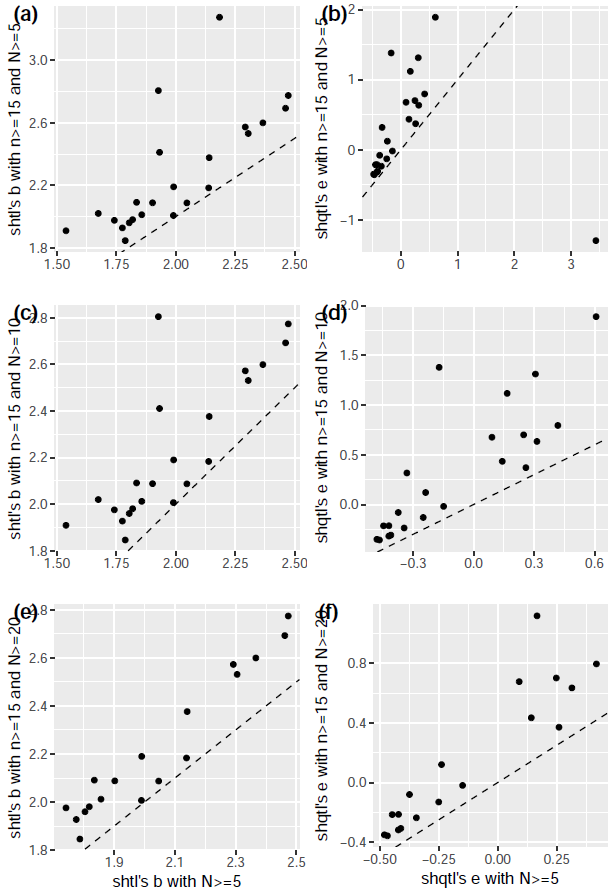


S46 Fig. Scatterplots of the point estimate of *b* of the spatial hierarchical TL (shtl) and *e* of the spatial hierarchical quadratic TL (shqtl) using count, with (vertical axis) and without (horizontal axis) the minimum number of sampling units requirement (*n* ≥ 15). When the requirement is applied, the number of mean-variance pairs (*N*) used in each shtl or shqtl testing is at least five ((a) and (b)), ten ((c) and (d)), and 20 ((e) and (f)). The horizontal axis in each column shows a point estimate of *b* of shtl ((a), (c) and (e)) and *e* of shqtl ((b), (d) and (f)) fitted by ols across at least five mean-variance pairs with no restriction on *n* for each pair. Dashed lines are 1:1 reference lines.

S47 Fig


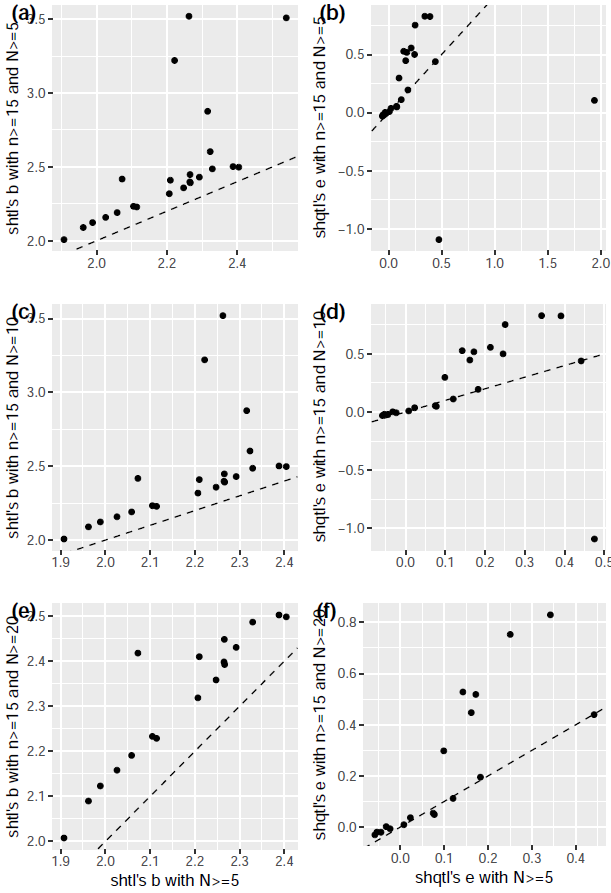


S47 Fig. Scatterplots of the point estimate of *b* of the spatial hierarchical TL (shtl) and *e* of the spatial hierarchical quadratic TL (shqtl) using density, with (vertical axis) and without (horizontal axis) the minimum number of sampling units requirement (*n* ≥ 15). The horizontal axes, vertical axes and dashed lines are defined in S46 Fig.

S48 Fig


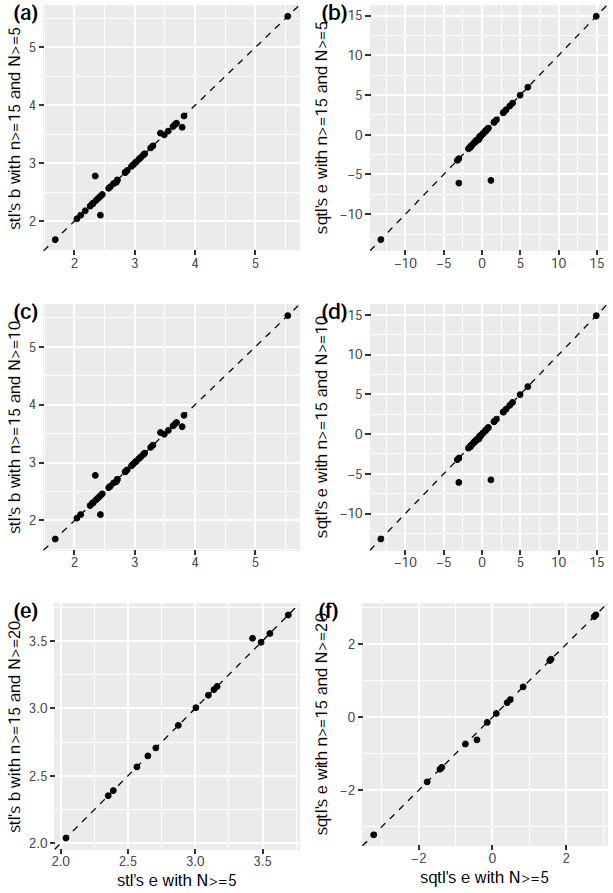


S48 Fig. Scatterplots of the point estimate of *b* of the spatial TL (stl) and *e* of the spatial quadratic TL (sqtl) using count, with (vertical axis) and without (horizontal axis) the minimum number of sampling units requirement (*n* ≥ 15). The horizontal axes, vertical axes and dashed lines are defined in S46 Fig.

S49 Fig


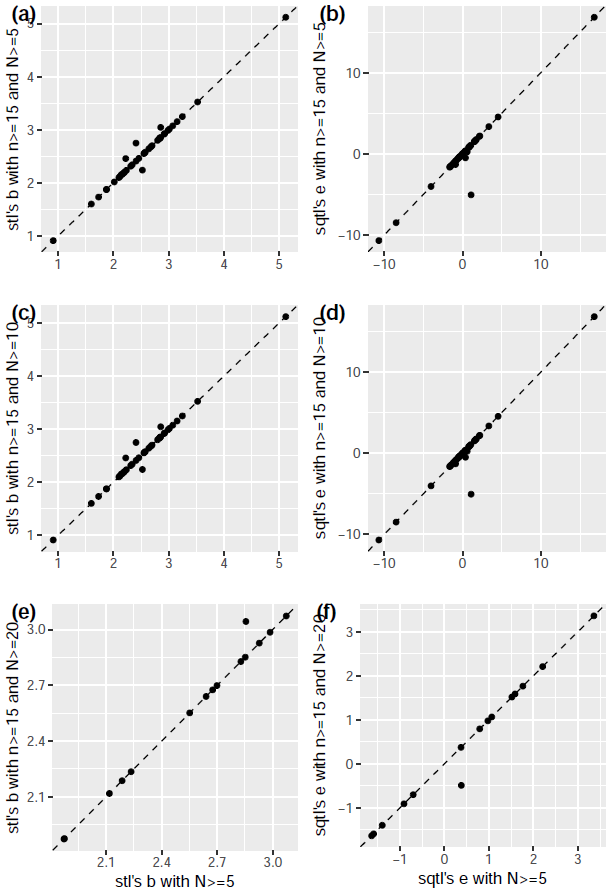


S49 Fig. Scatterplots of the point estimate of *b* of the spatial TL (stl) and *e* of the spatial quadratic TL (sqtl) using density, with (vertical axis) and without (horizontal axis) the minimum number of sampling units requirement (*n* ≥ 15). The horizontal axes, vertical axes and dashed lines are defined in S46 Fig.

S50 Fig


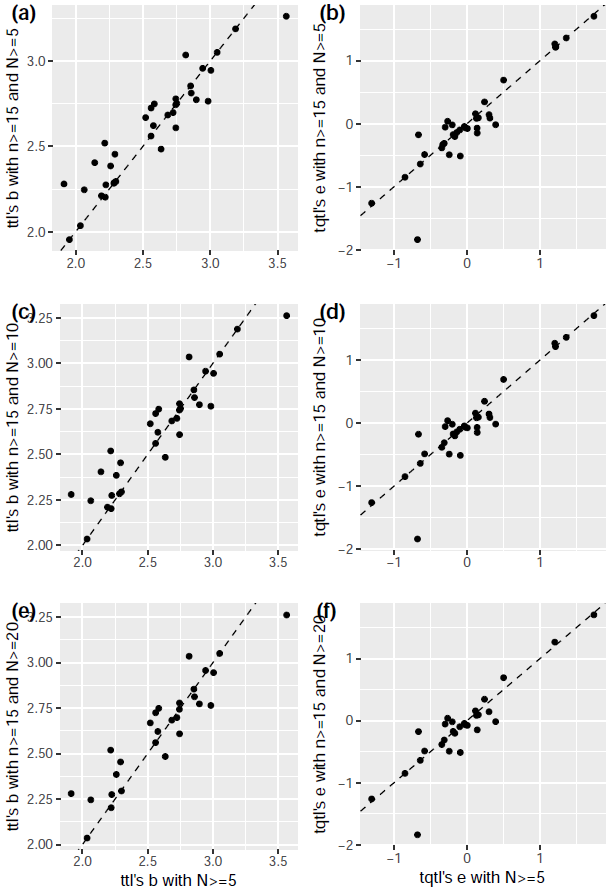


S50 Fig. Scatterplots of the point estimate of *b* of the temporal TL (ttl) and *e* of the temporal quadratic TL (tqtl) using count, with (vertical axis) and without (horizontal axis) the minimum number of sampling units requirement (*n* ≥ 15). The horizontal axes, vertical axes and dashed lines are defined in S46 Fig.

S51 Fig


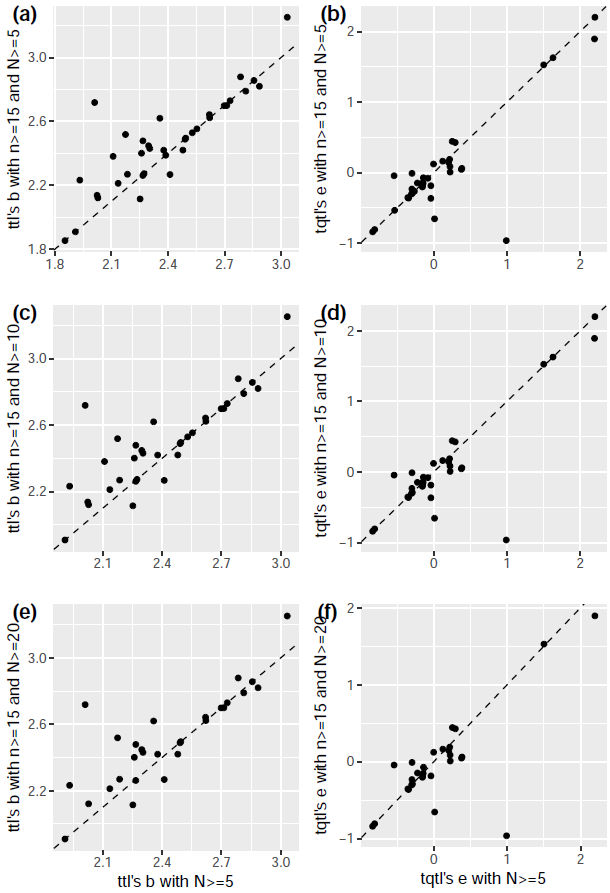


S51 Fig. Scatterplots of the point estimate of *b* of the temporal TL (ttl) and *e* of the temporal quadratic TL (tqtl) using density, with (vertical axis) and without (horizontal axis) the minimum number of sampling units requirement (*n* ≥ 15). The horizontal axes, vertical axes and dashed lines are defined in S46 Fig.

# Supporting table captions

S1 Table. Ordinary least-squares (ols) linear regression statistics of spatial hierarchical Taylor's law, using count and density separately. N is the number of mean-variance pairs (states) in each census. est is the point estimate of slope or intercept. std err is the standard error. p is the p-value of a test of the null hypothesis that the slope or intercept is zero, or of the null hypothesis that the overall quadratic regression is not substantially better than the intercept-only model. Bold font indicates that p-value < 0.05. * indicates that p-value < 0.0022 (= 0.05/23 by Bonferroni correction). adj.R2 is the adjusted coefficient of determination.

S2 Table. Ordinary least-squares (ols) quadratic regression statistics of spatial hierarchical quadratic Taylor's law, using count and density separately. Notations are defined in the caption of S1 Table.

S3 Table. Ordinary least-squares (ols) linear regression statistics of spatial Taylor's law, using count and density separately. N is the number of mean-variance pairs (censuses) in each state. Other notations are defined in the caption of S1 Table.

S4 Table. Ordinary least-squares (ols) quadratic regression statistics of spatial quadratic Taylor's law, using count and density separately. Notations are defined in the caption of S1 Table.

S5 Table. Ordinary least-squares (ols) linear regression statistics of temporal Taylor's law, using count and density separately. N is the number of mean-variance pairs (counties) within each state. Other notations are defined in the caption of S1 Table.

S6 Table. Ordinary least-squares (ols) quadratic regression statistics of temporal quadratic Taylor's law, using count and density separately. N is the number of mean-variance pairs (counties) within each state. Other notations are defined in the caption of S1 Table.

S7 Table. Summary of the regression statistics of the Taylor's law (TL) (eqn 1) and quadratic Taylor's law (QTL) (eqn 2) models using county population density. Notations are defined in the caption of Table 1.

S8 Table. Summary statistics of county area, county count and county density in each state and census. Spreadsheet "Definitions" lists the definitions of all 15 statistics.

S9 Table. Diagnostic statistics of the ols linear regression for spatial hierarchical Taylor's law, using count and density separately. Except columns "measure," "year," and "outlier," all columns show the p-value of the corresponding hypothesis test and census. "outlier" column shows the name of the outlier(s), if any.

S10 Table. Diagnostic statistics of the ols quadratic regression for spatial hierarchical quadratic Taylor's law, using count and density separately. Column definitions and notations follow S9 Table.

S11 Table. Diagnostic statistics of the ols linear regression for spatial Taylor's law, using count and density separately. Column definitions and notations follow S9 Table.

S12 Table. Diagnostic statistics of the ols quadratic regression for spatial quadratic Taylor's law, using count and density separately. Column definitions and notations follow S9 Table.

S13 Table. Diagnostic statistics of the ols linear regression for temporal Taylor's law, using count and density separately. Column definitions and notations follow S9 Table.

S14 Table. Diagnostic statistics of the ols quadratic regression for temporal quadratic Taylor's law, using count and density separately. Column definitions and notations follow S9 Table.

S15 Table. Ordinary least-squares (ols) linear regression statistics of the spatial hierarchical TL with the minimum number of sampling units requirement (n ≥ 15), using count and density separately. "est_X_ols," "err_X_ols" and "p_X_ols" denote the point estimate, standard error and p-value of the corresponding regression coefficient ("int" = intercept, "slp" = slope) from ols. "adjr2" denotes the adj. R2 of the ols linear regression. N is the number of mean-variance pairs used in testing each TL.

S16 Table. Ordinary least-squares quadratic regression statistics of the spatial hierarchical quadratic Taylor's law (QTL) with the minimum number of sampling units requirement (n ≥ 15), using count and density separately. Columns are defined in Table S15. "qua" = quadratic coefficient.

S17 Table. Ordinary least-squares linear regression statistics of the spatial TL with the minimum number of sampling units requirement (n ≥ 15), using count and density separately. Columns are defined in S15 Table.

S18 Table. Ordinary least-squares quadratic regression statistics of the spatial QTL with the minimum number of sampling units requirement (n ≥ 15), using count and density separately. Columns are defined in S15 Table.

S19. Ordinary least-squares linear regression statistics of the temporal TL with the minimum number of sampling units requirement (n ≥ 15), using count and density separately. Columns are defined in S15 Table.

S20 Table. Ordinary least-squares quadratic regression statistics of the temporal QTL with the minimum number of sampling units requirement (n ≥ 15), using count and density separately. Columns are defined in S15 Table.

S21 Table. Diagnostic statistics of the ols linear regression for the spatial hierarchical TL with the minimum number of sampling units requirement (n ≥ 15), using count and density separately. Columns are defined in S9 Table.

S22 Table. Diagnostic statistics of the ols quadratic regression for the spatial hierarchical QTL with the minimum number of sampling units requirement (n ≥ 15), using count and density separately. Columns are defined in S9 Table.

S23 Table. Diagnostic statistics of the ols linear regression for the spatial TL with the minimum number of sampling units requirement (n ≥ 15), using count and density separately. Columns are defined in S9 Table.

S24 Table. Diagnostic statistics of the ols quadratic regression for the spatial QTL with the minimum number of sampling units requirement (n ≥ 15), using count and density separately. Columns are defined in S9 Table.

S25 Table. Diagnostic statistics of the ols linear regression for the temporal TL with the minimum number of sampling units requirement (n ≥ 15), using count and density separately. Columns are defined in S9 Table.

S26 Table. Diagnostic statistics of the ols quadratic regression for the temporal QTL with the minimum number of sampling units requirement (n ≥ 15), using count and density separately. Columns are defined in S9 Table.
