# Supplementary figures and images for: Analyzing and interpreting spatial and temporal variability of the United States county population distributions using Taylor's law
Source: PLoS One. 2019 Dec 11;14(12):e0226096. doi: 10.1371/journal.pone.0226096 (PMC6905577; doi:10.1371/journal.pone.0226096)

spatial variance of county population count

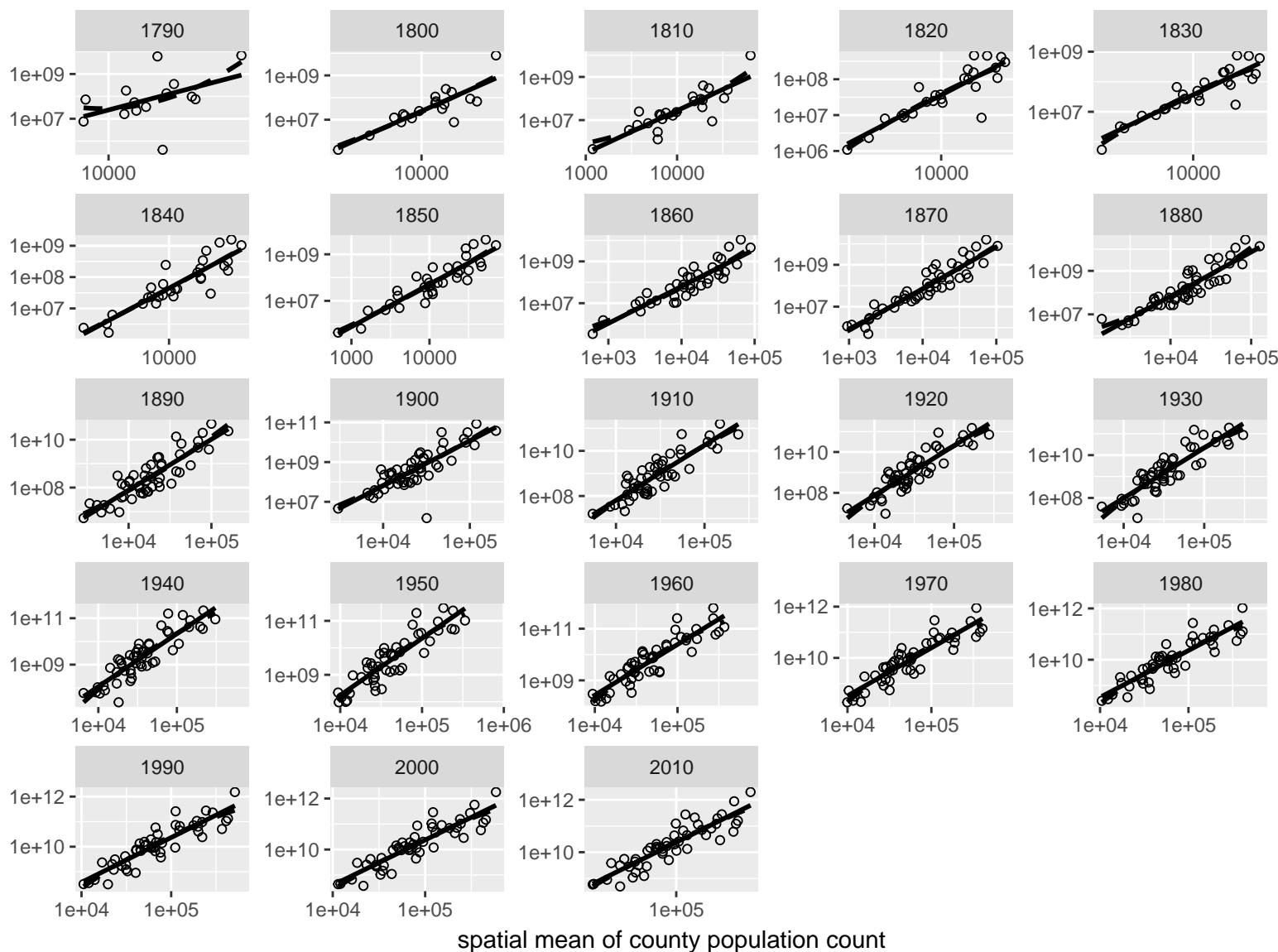

Supplement: S1 Fig — (PDF) [file pone.0226096.s002.pdf]

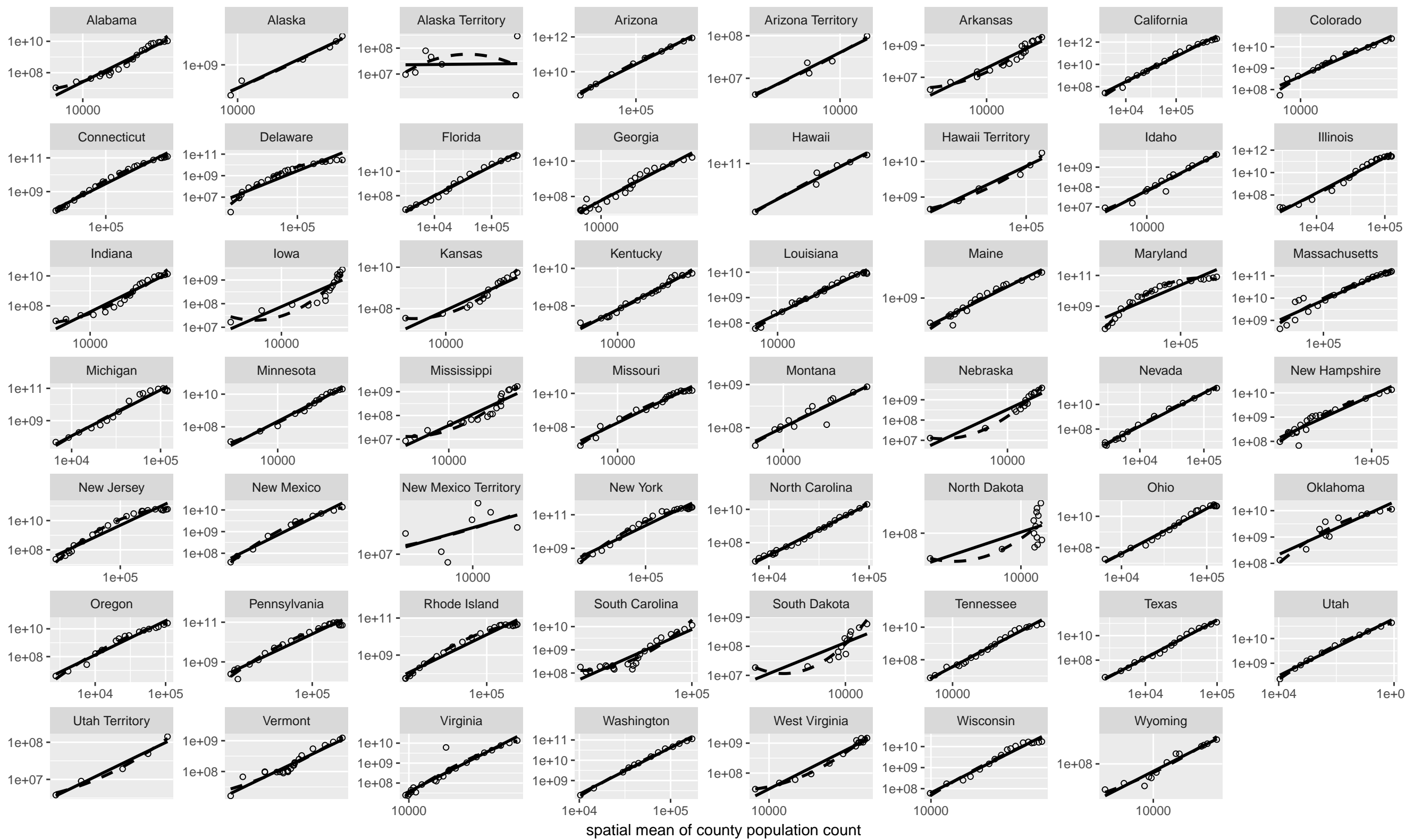

Supplement: S2 Fig — (PDF) [file pone.0226096.s003.pdf]

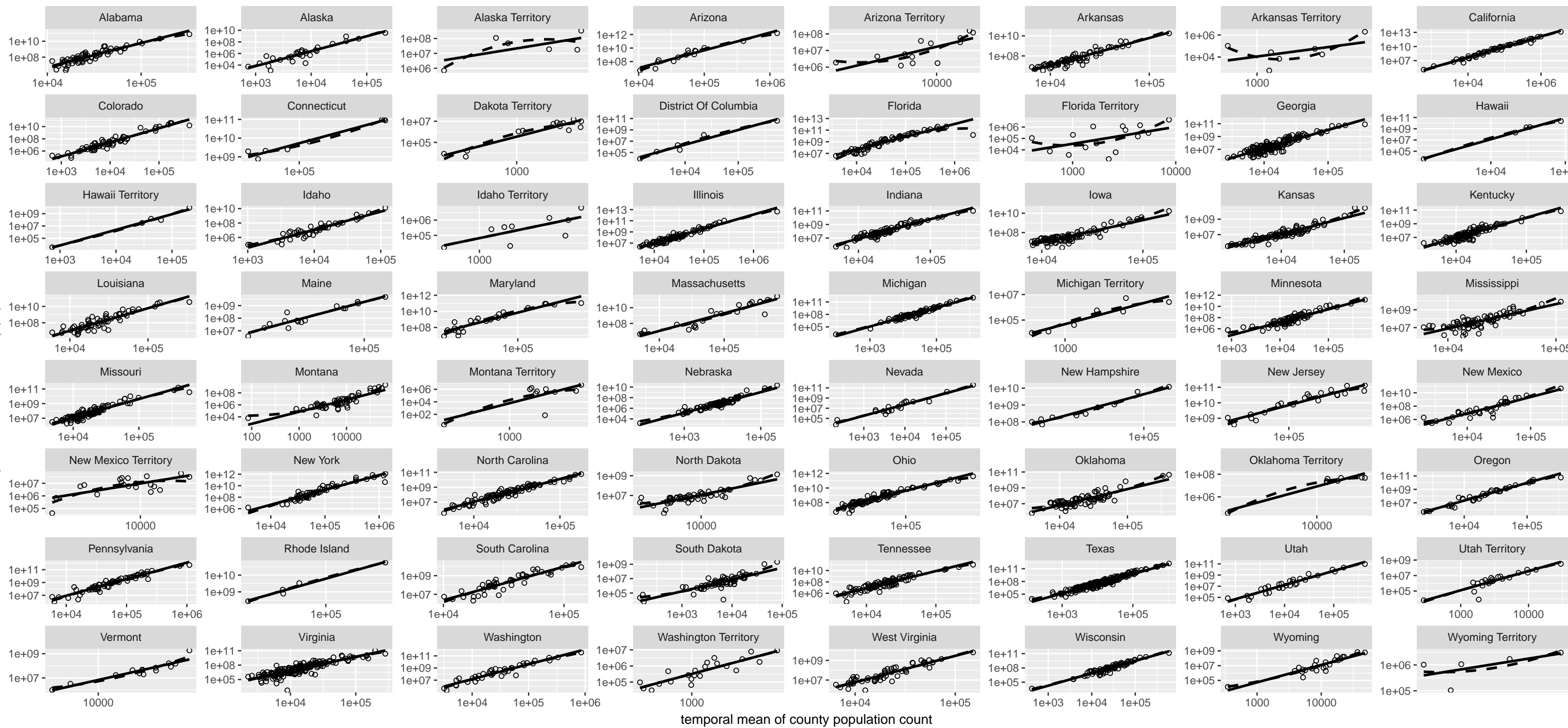

Supplement: S3 Fig — (PDF) [file pone.0226096.s004.pdf]

frequency

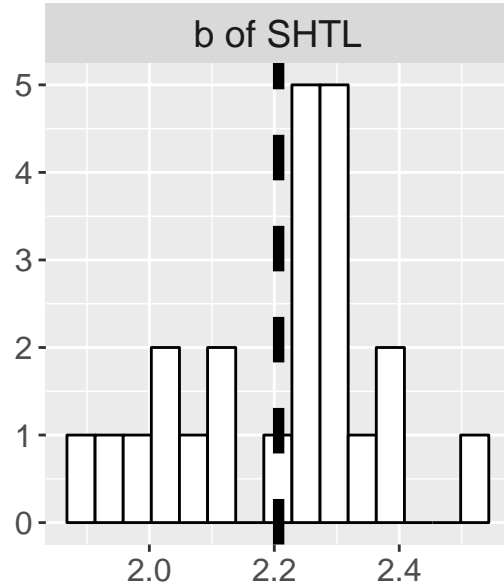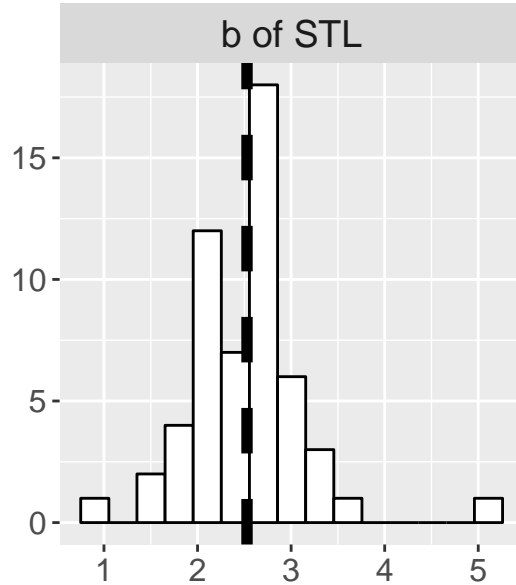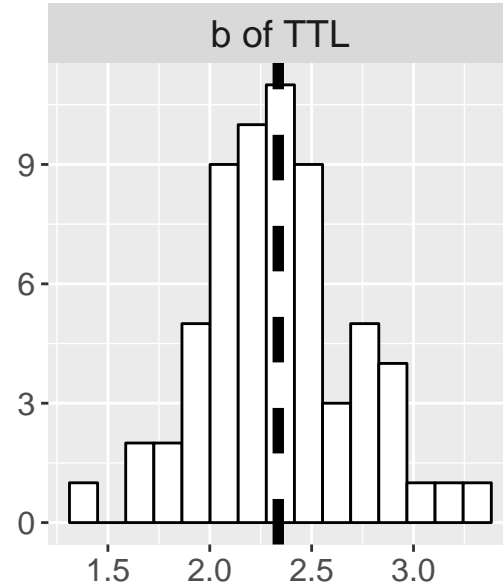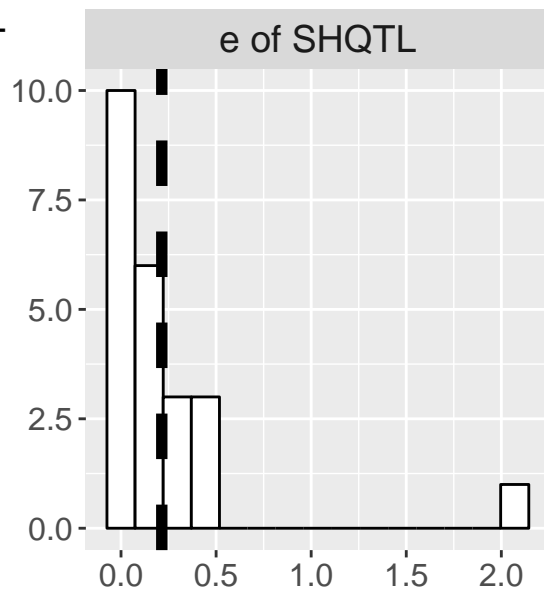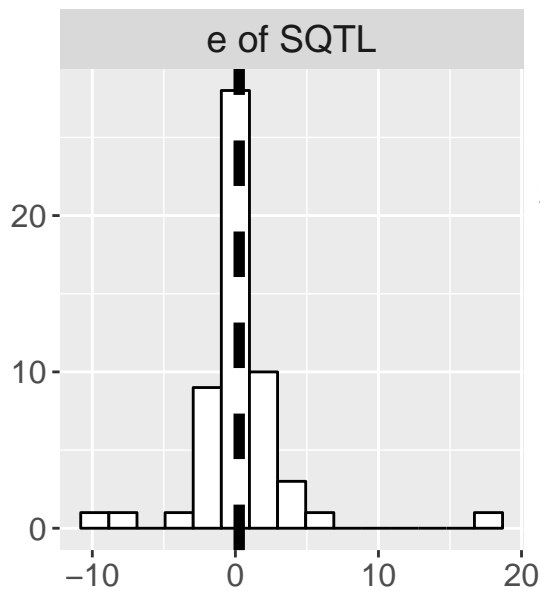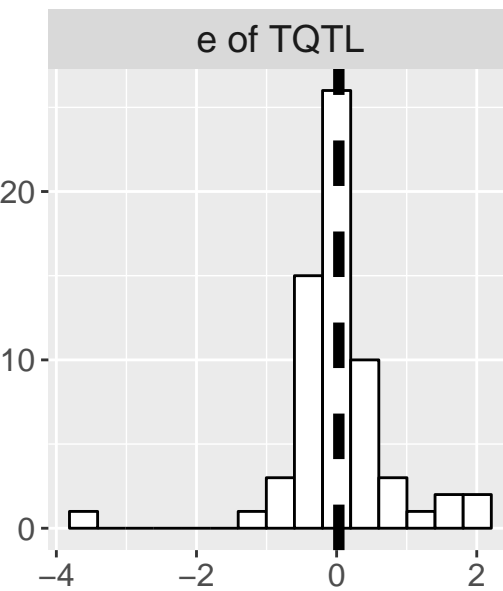

Supplement: S4 Fig — (PDF) [file pone.0226096.s005.pdf]

spatial variance of county population density

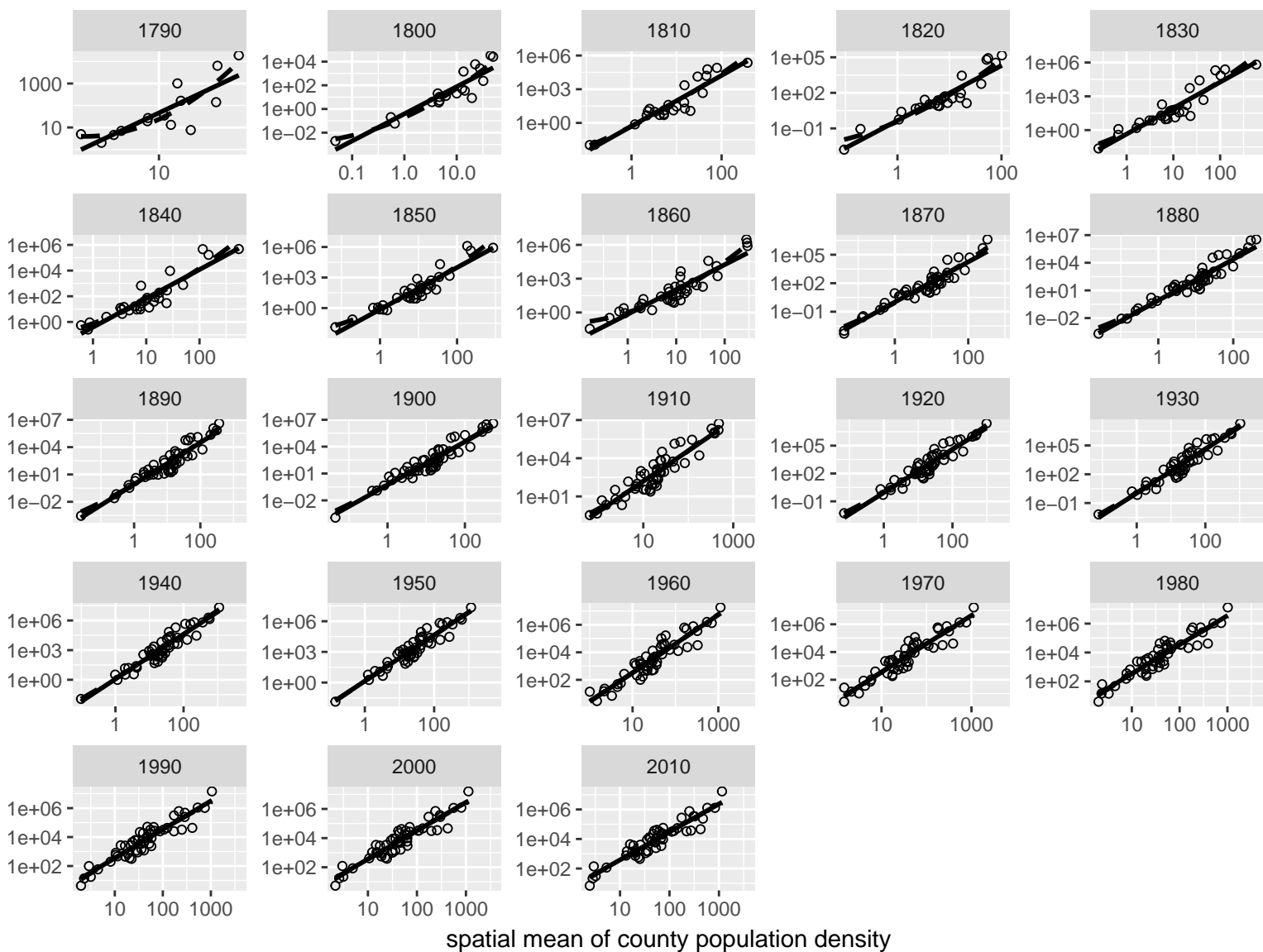

Supplement: S5 Fig — (PDF) [file pone.0226096.s006.pdf]

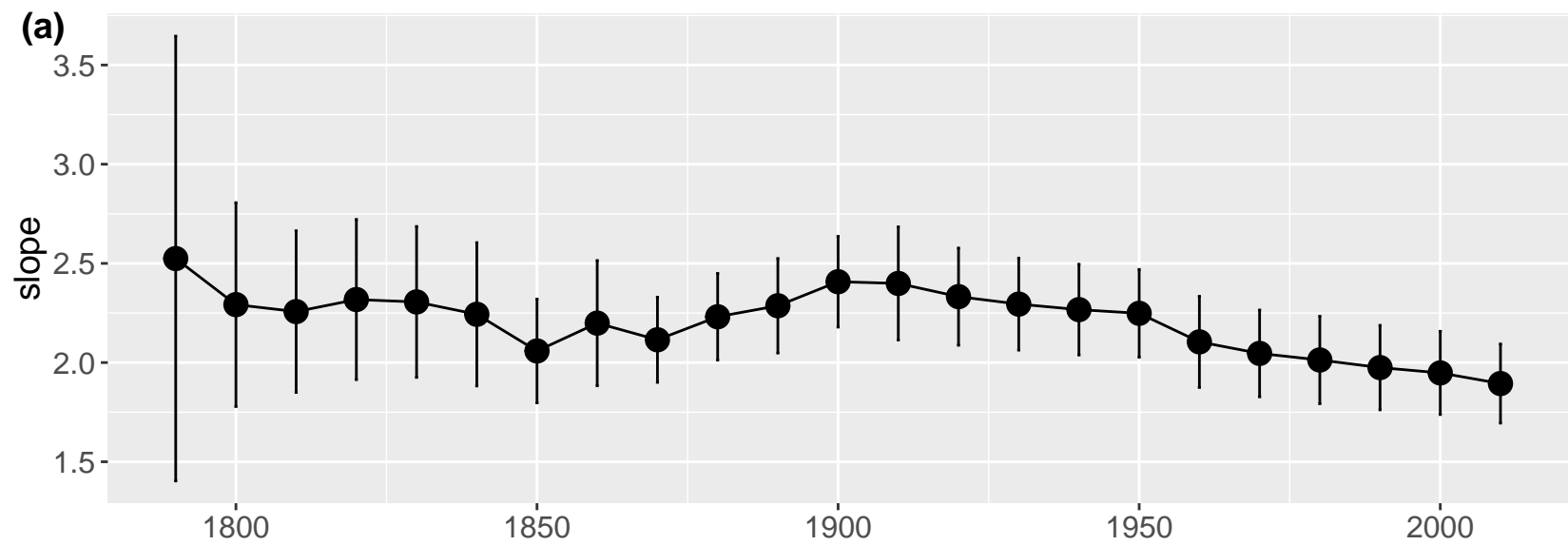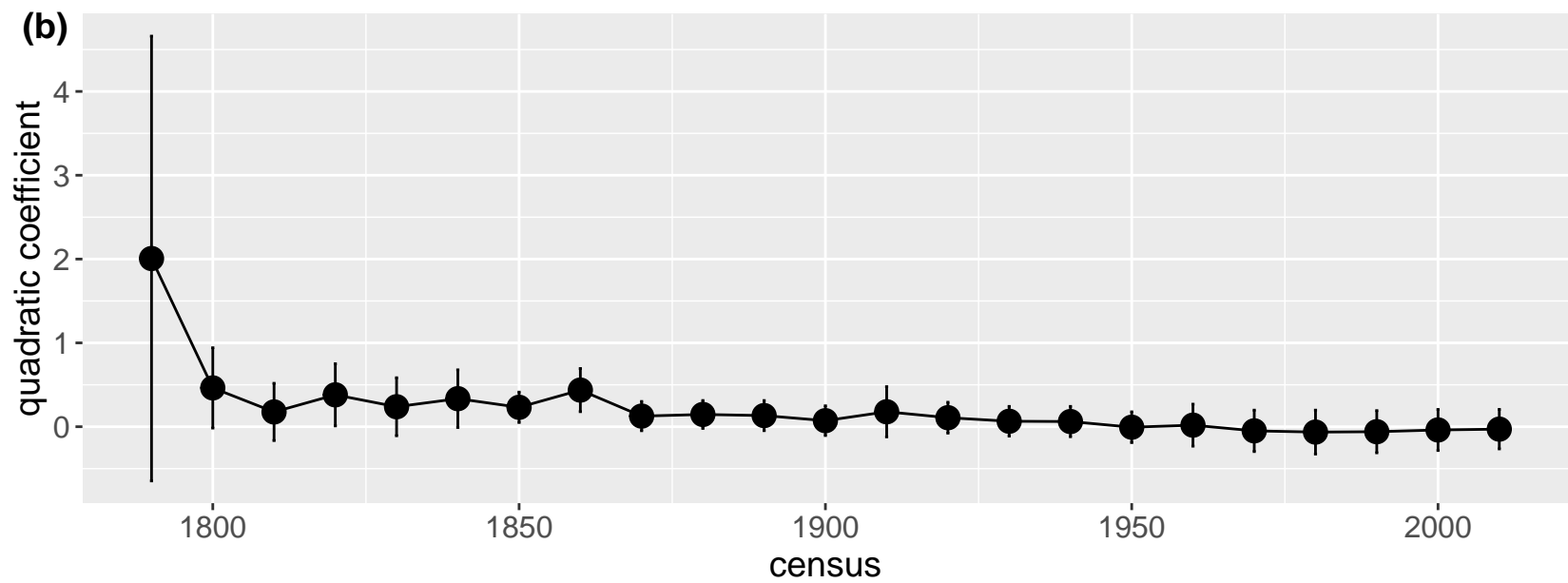

Supplement: S6 Fig — Time series of (a) the slope estimate of spatial hierarchical TL and (b) quadratic coefficient of spatial hierarchical QTL using county population density. (PDF) [file pone.0226096.s007.pdf]

spatial variance of county population density

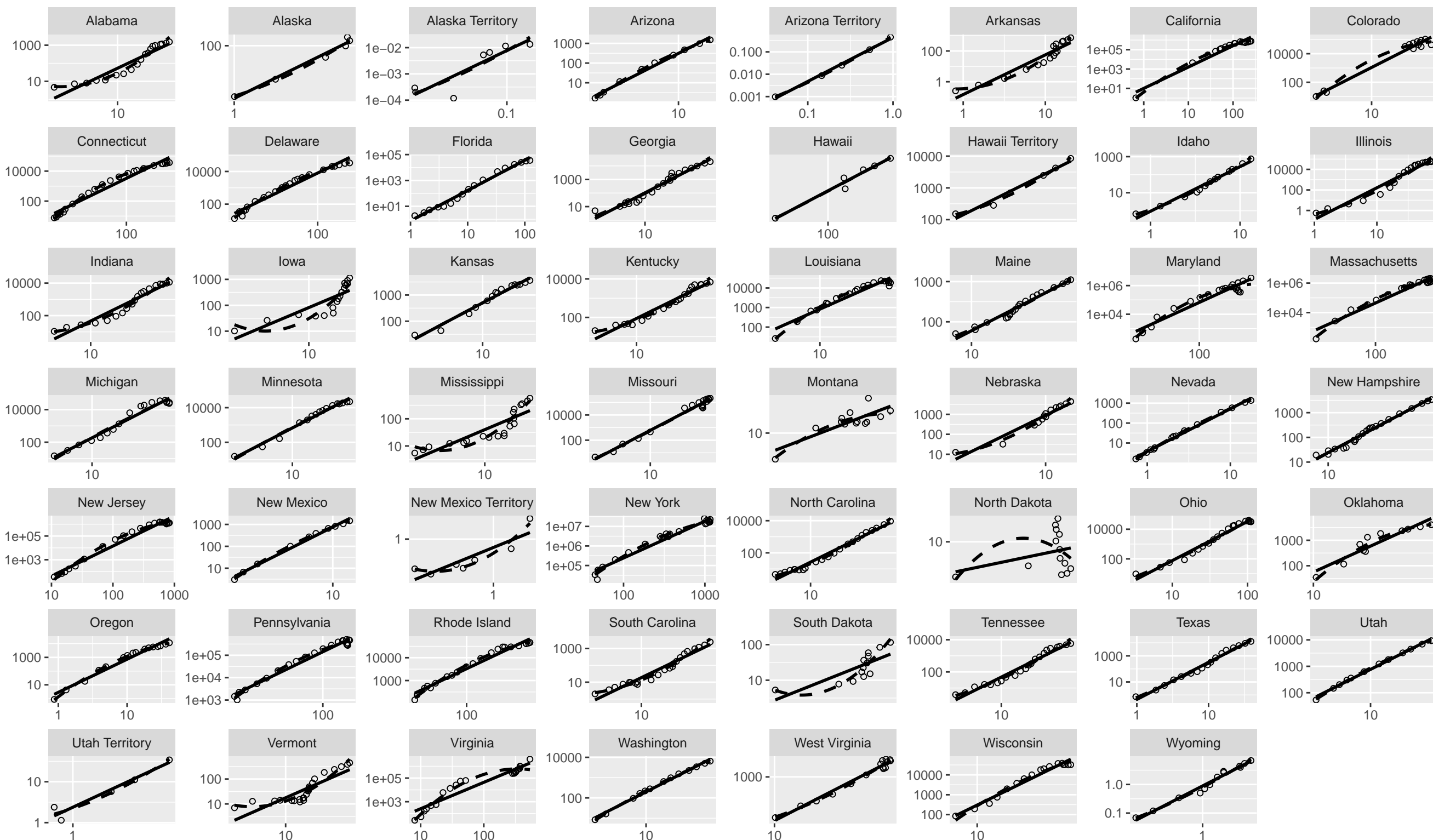

spatial mean of county population density

Supplement: S7 Fig — (PDF) [file pone.0226096.s008.pdf]

(a)

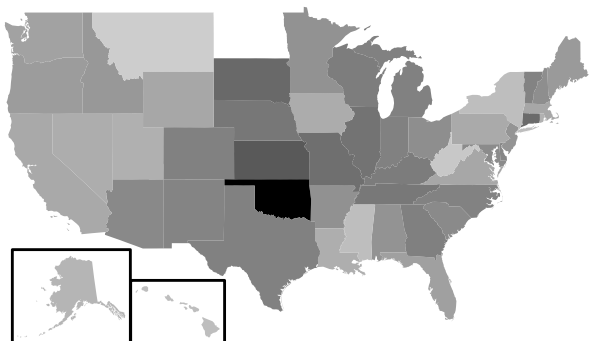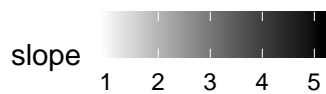

(b)

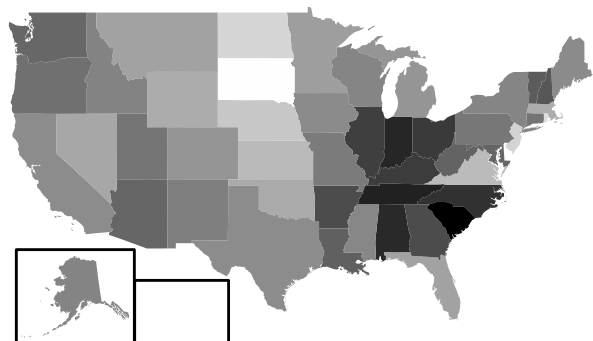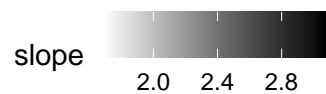

(c)

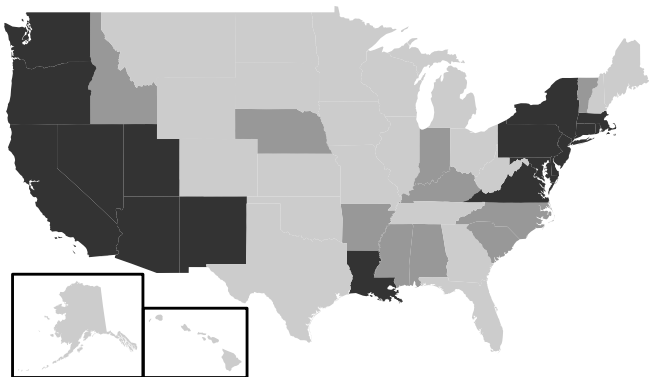

quadratic coefficient

minus plus zero

(d)

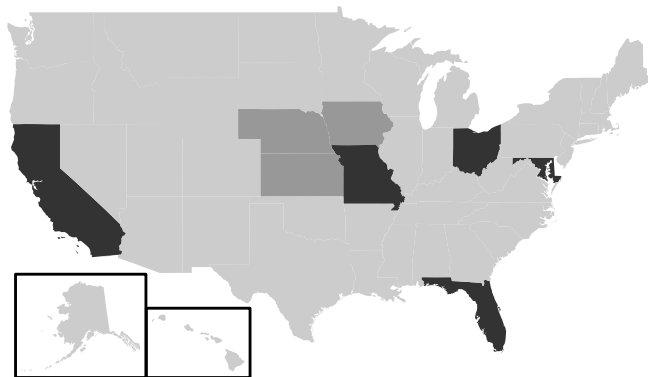

quadratic coefficient

minus plus zero

Supplement: S8 Fig — Point estimates of the slope of (a) spatial TL and (b) temporal TL, and the sign of the quadratic coefficients of (c) spatial QTL and (d) temporal QTL by US states, using county population density. (PDF) [file pone.0226096.s009.pdf]

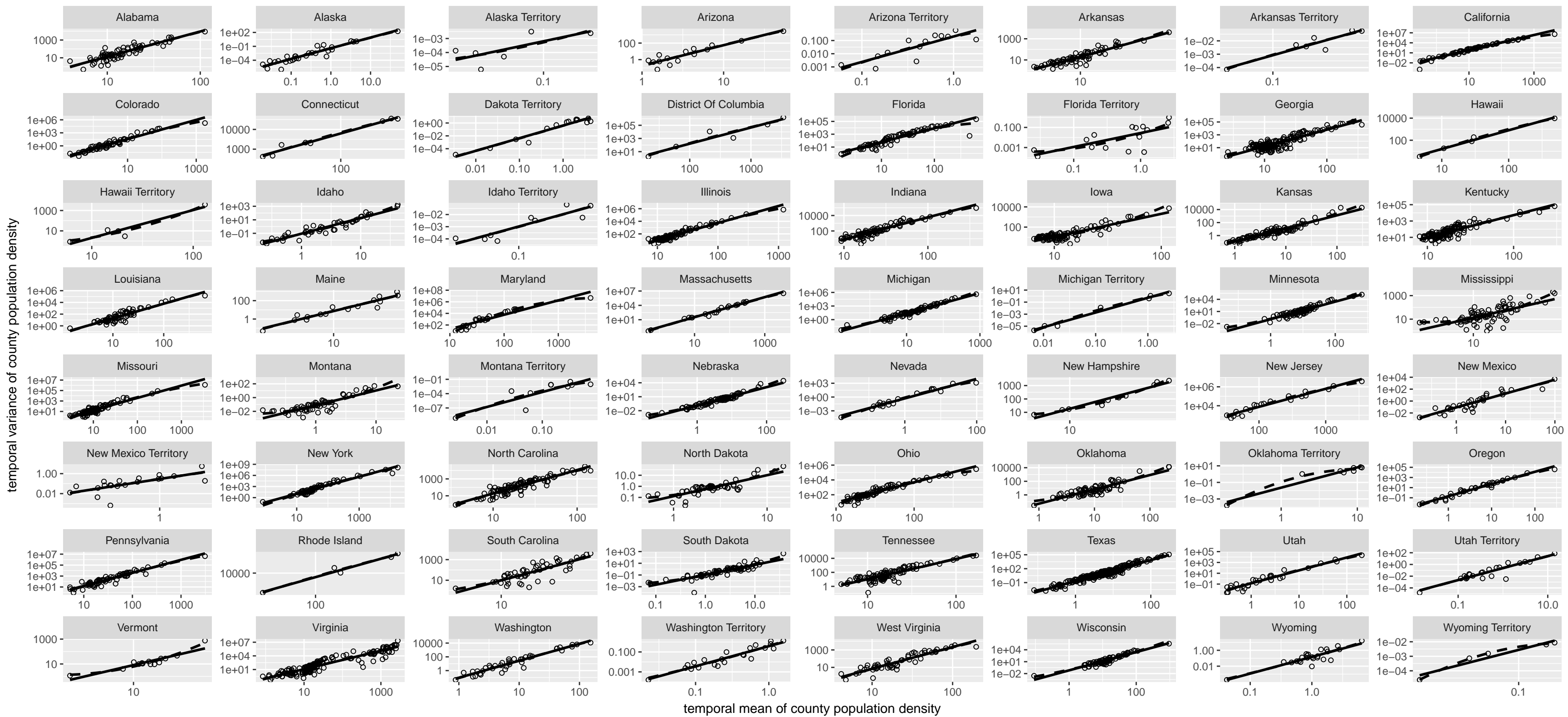

Supplement: S9 Fig — (PDF) [file pone.0226096.s010.pdf]

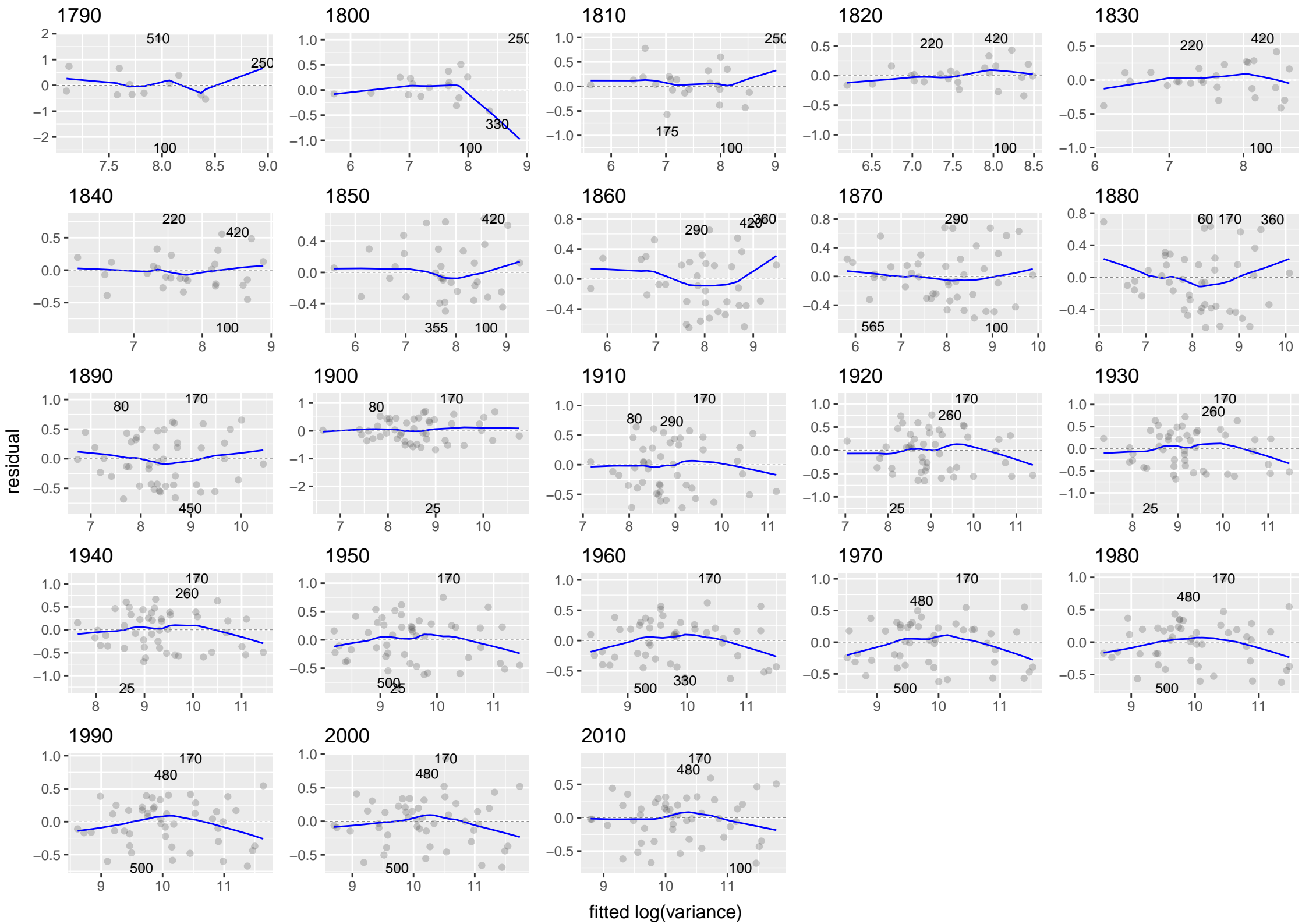

Supplement: S10 Fig — (PDF) [file pone.0226096.s011.pdf]

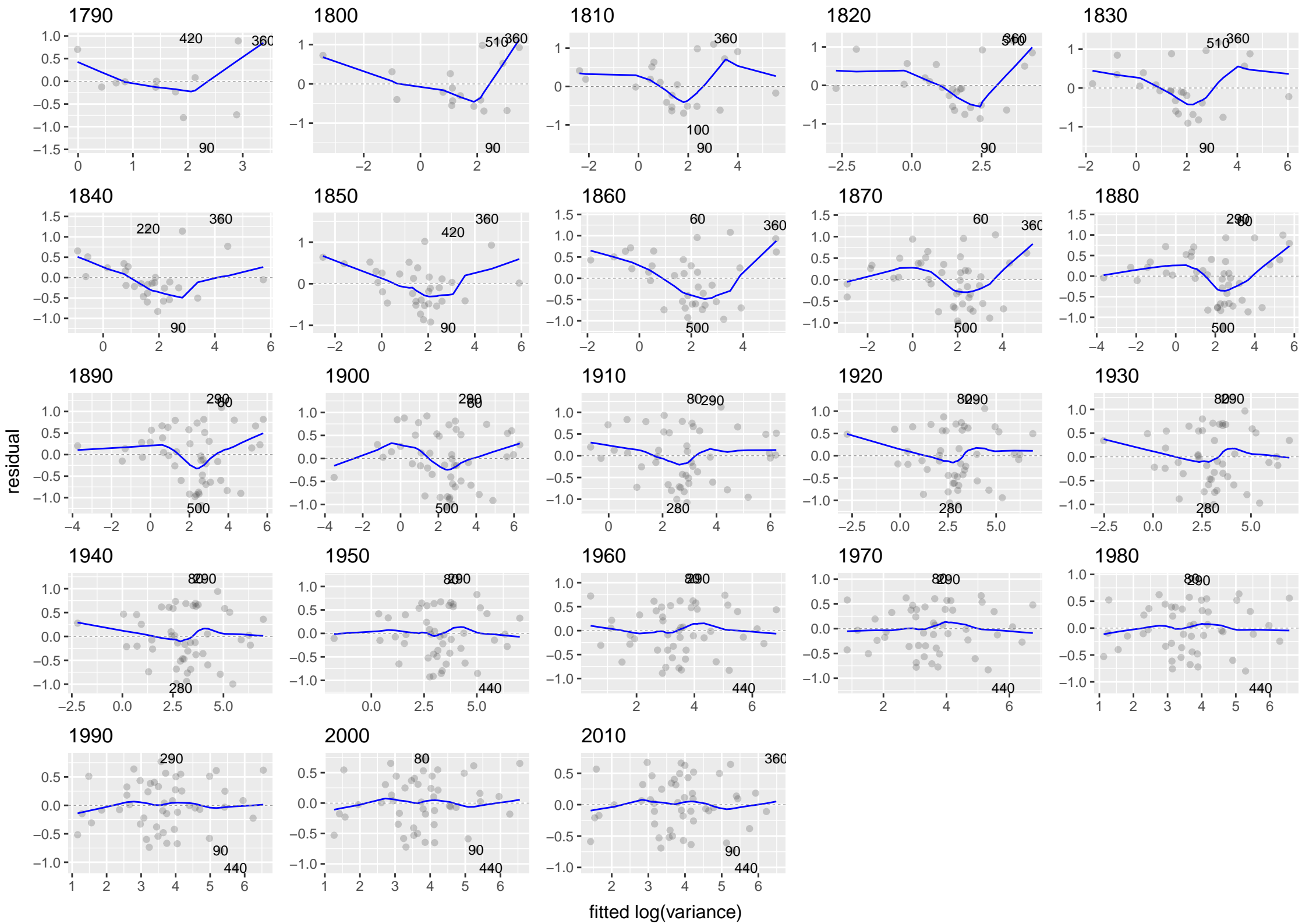

Supplement: S11 Fig — (PDF) [file pone.0226096.s012.pdf]

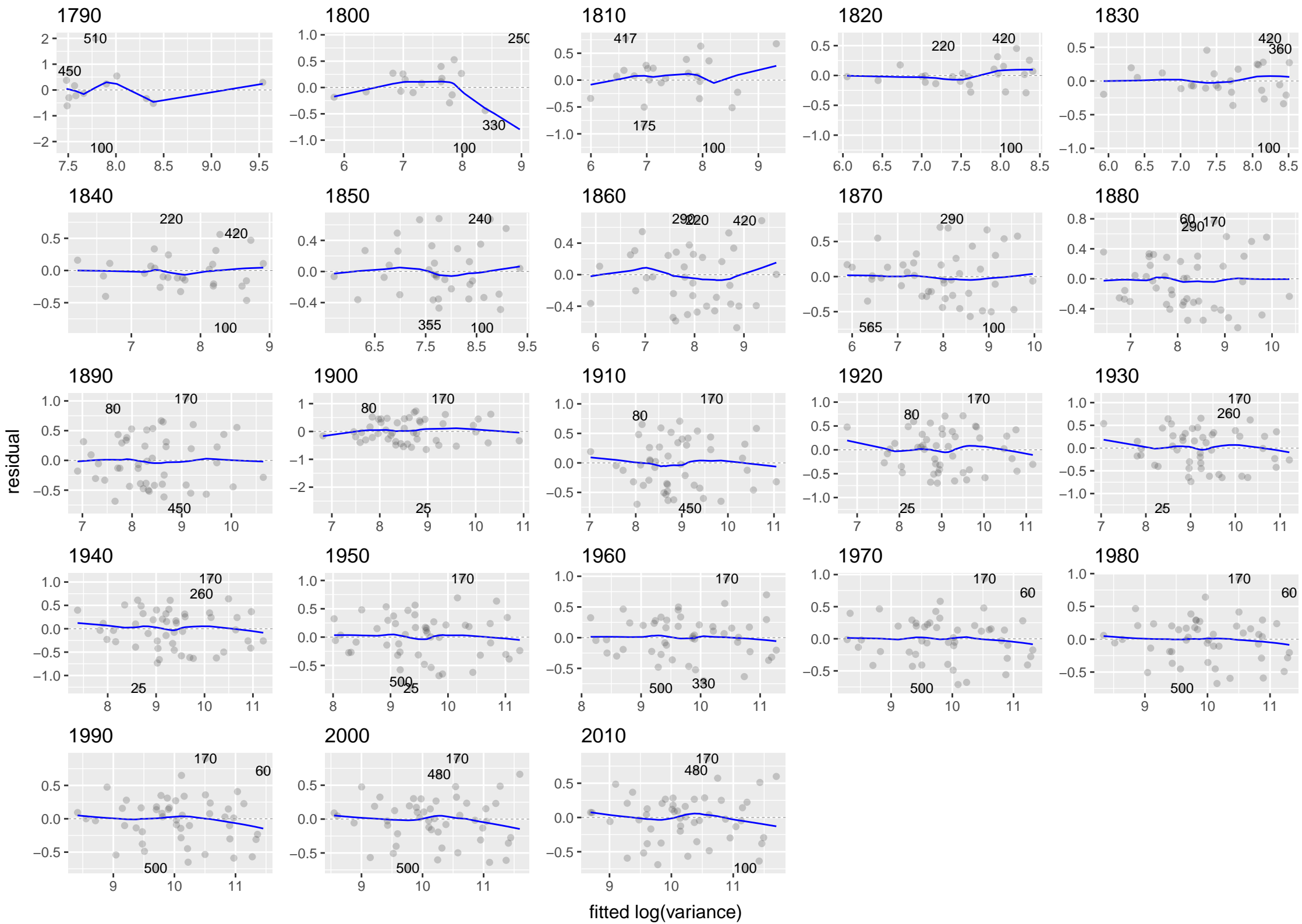

Supplement: S12 Fig — (PDF) [file pone.0226096.s013.pdf]

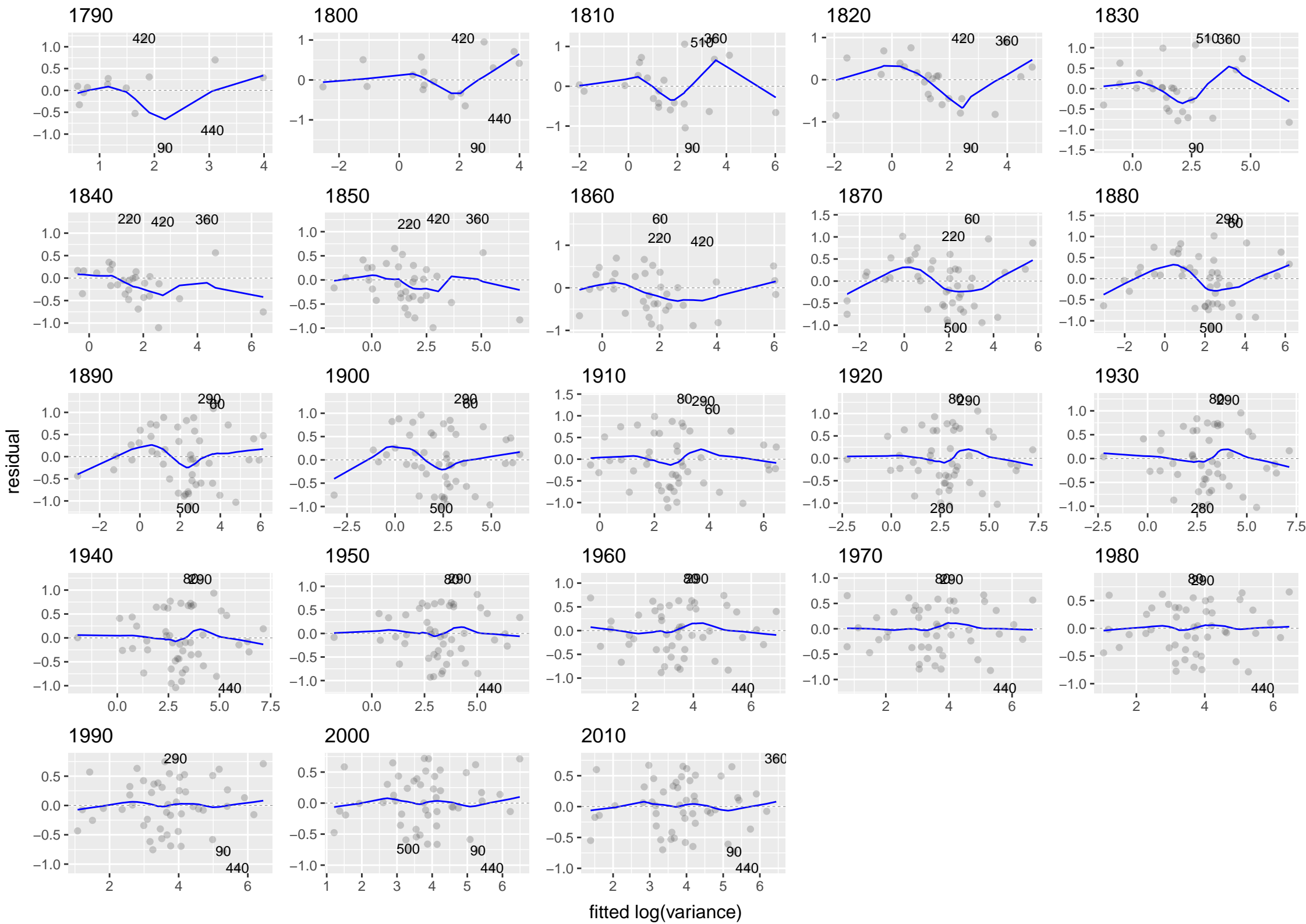

Supplement: S13 Fig — (PDF) [file pone.0226096.s014.pdf]

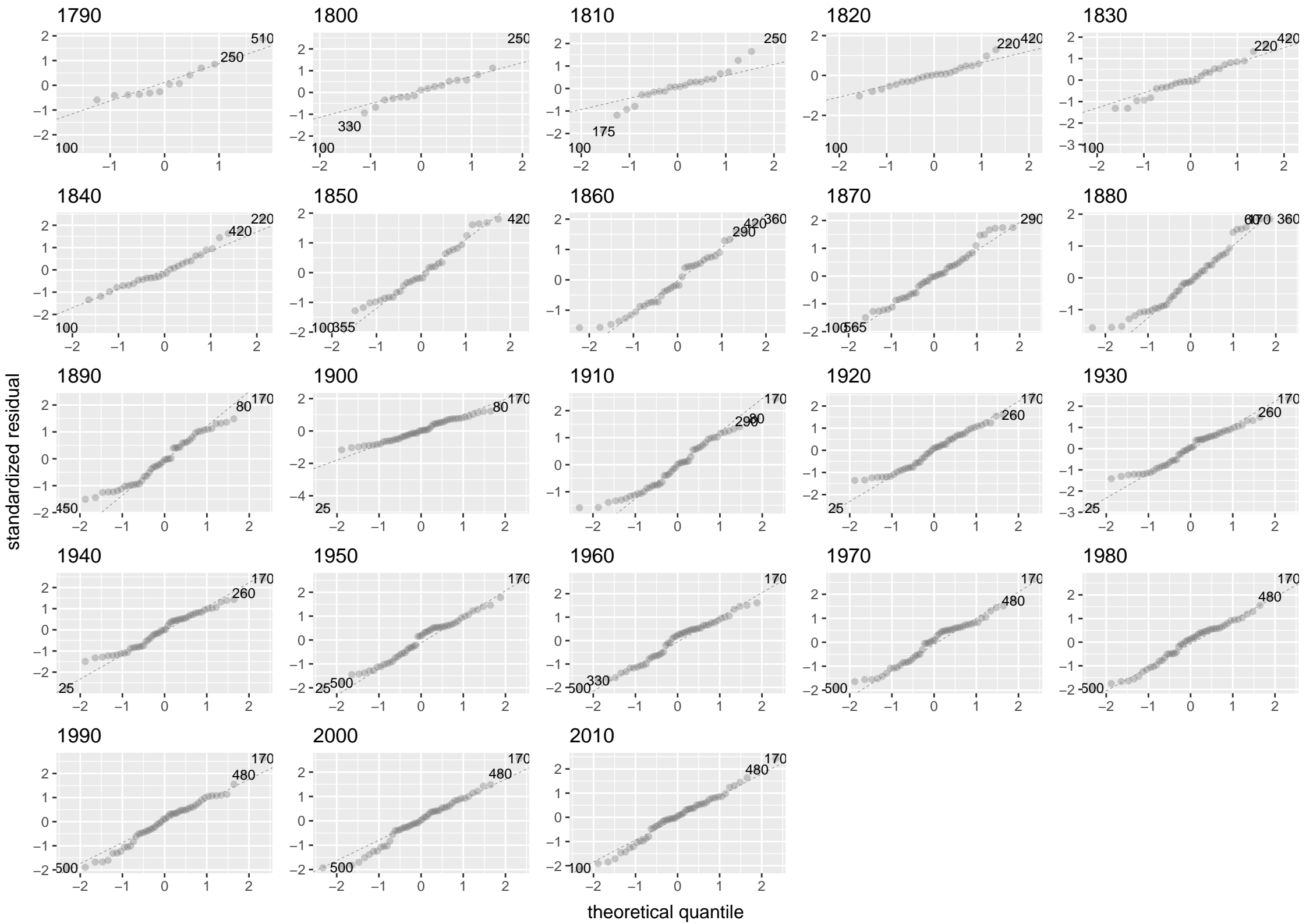

Supplement: S14 Fig — (PDF) [file pone.0226096.s015.pdf]

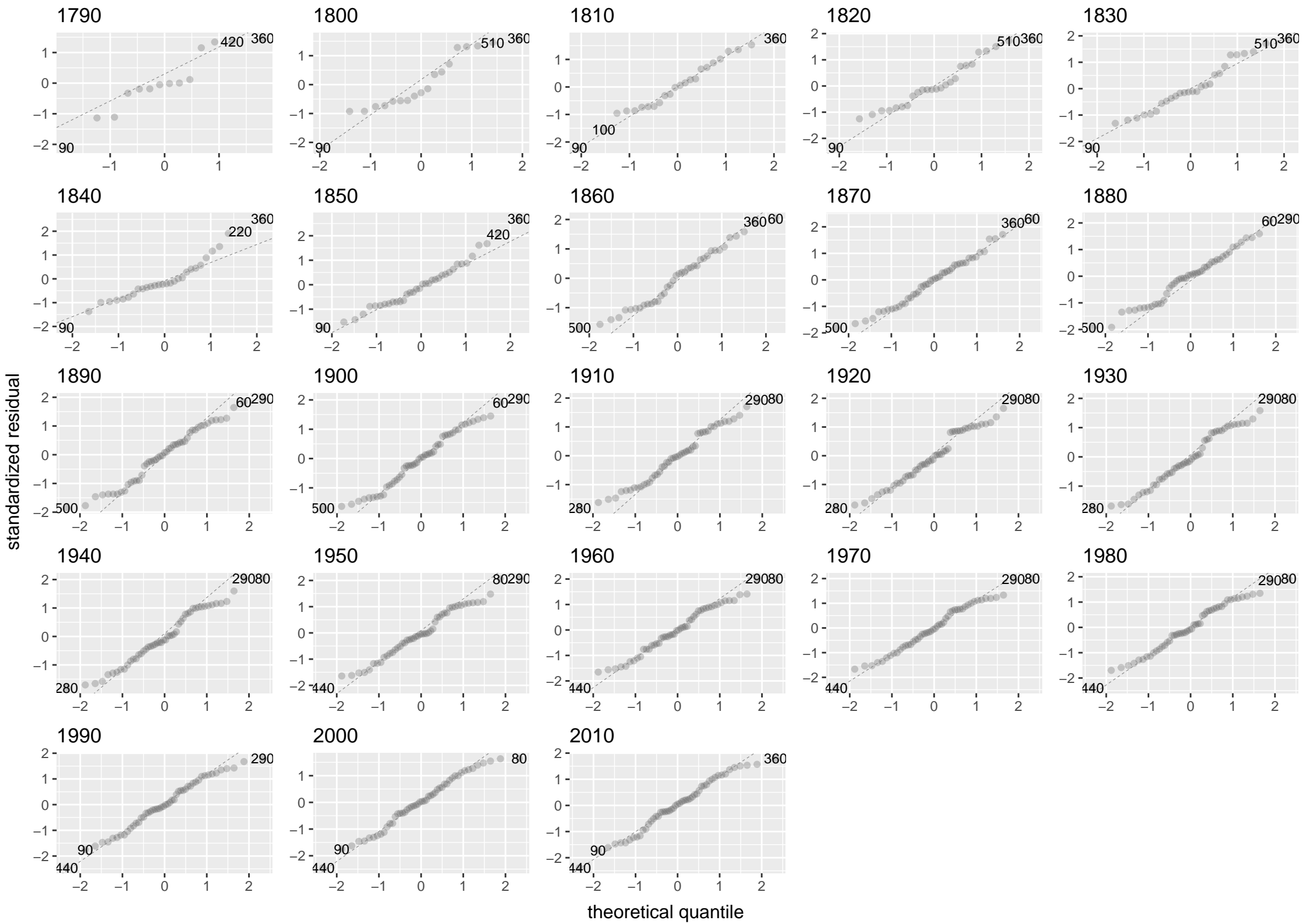

Supplement: S15 Fig — (PDF) [file pone.0226096.s016.pdf]

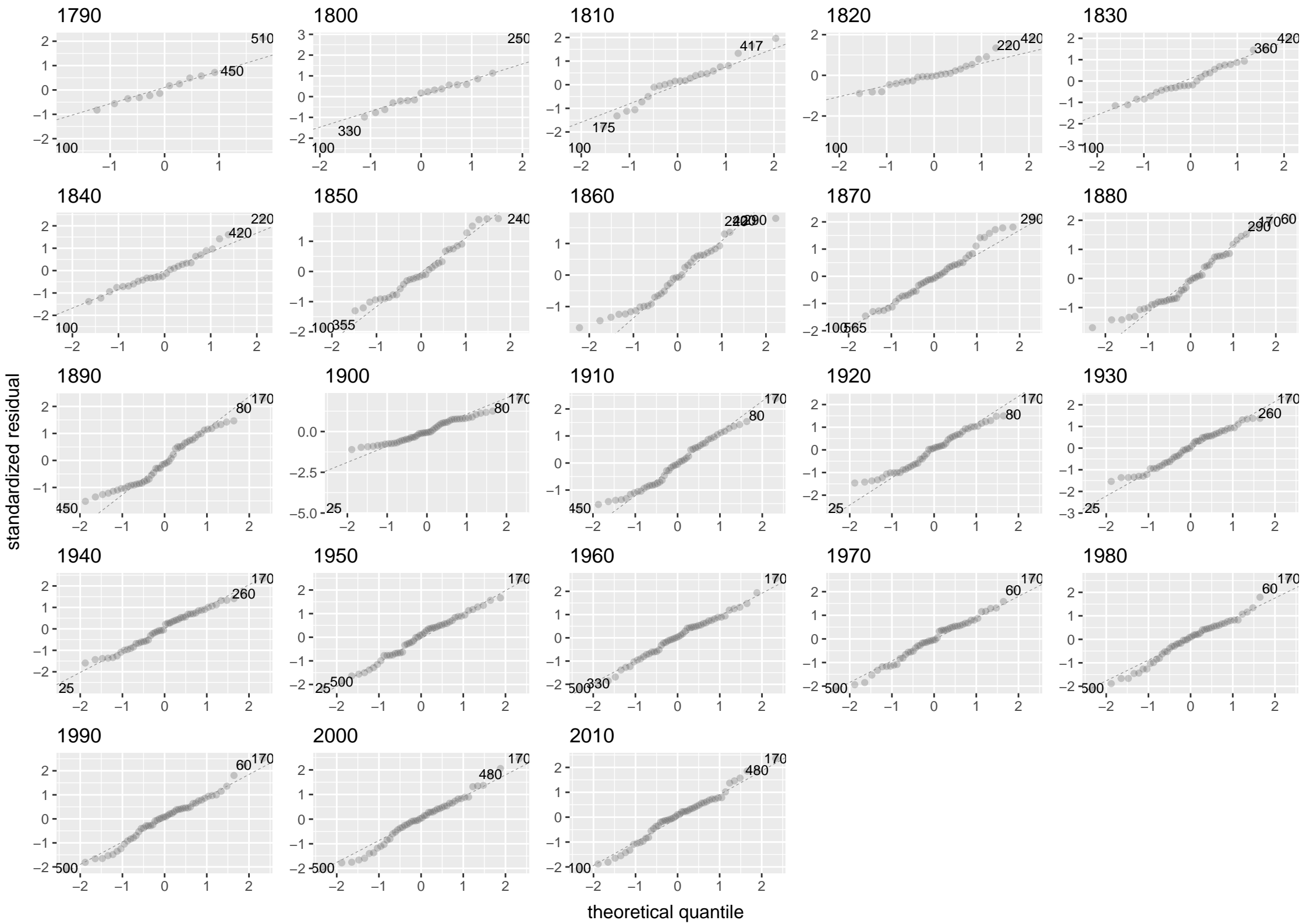

Supplement: S16 Fig — (PDF) [file pone.0226096.s017.pdf]

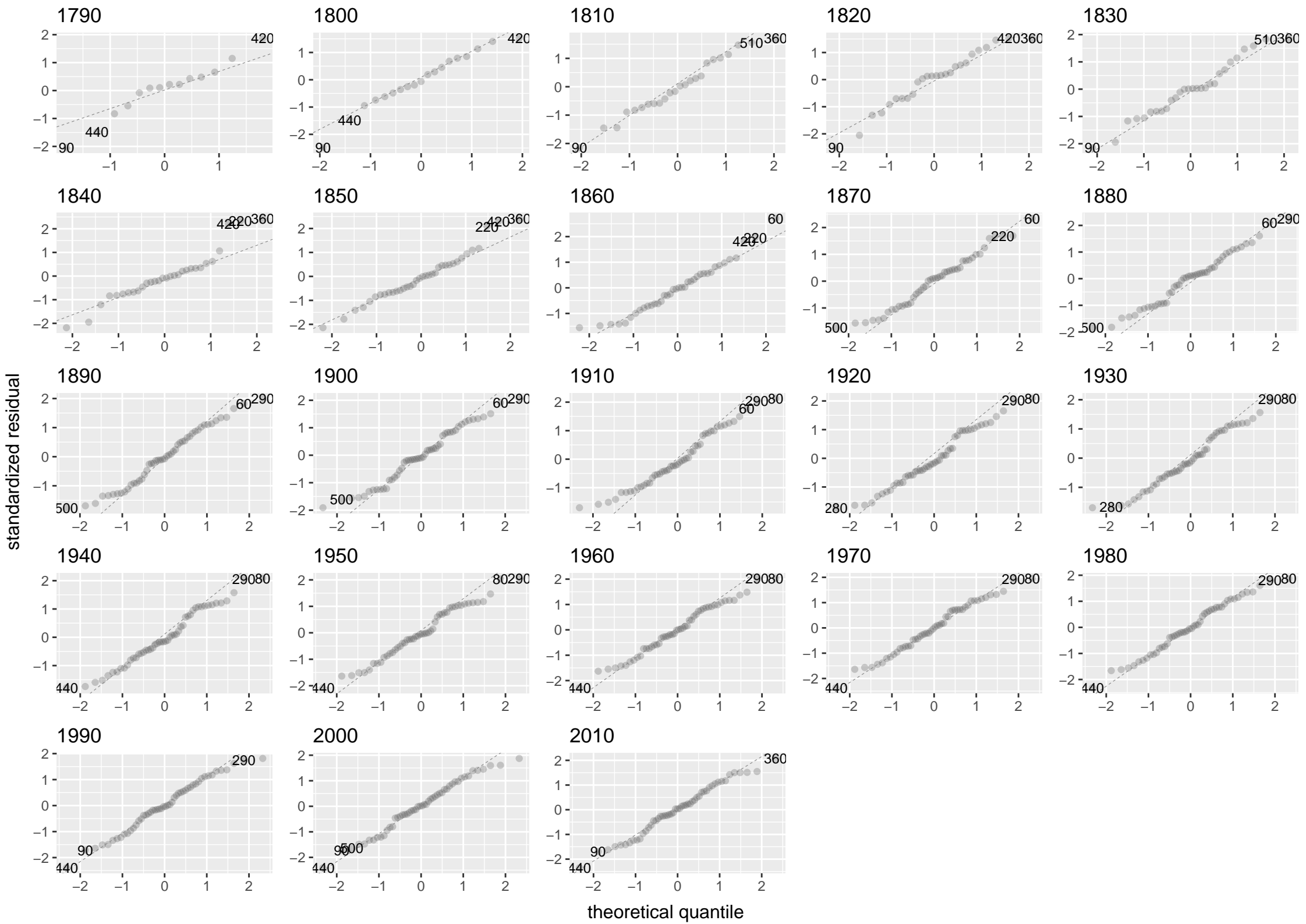

Supplement: S17 Fig — (PDF) [file pone.0226096.s018.pdf]

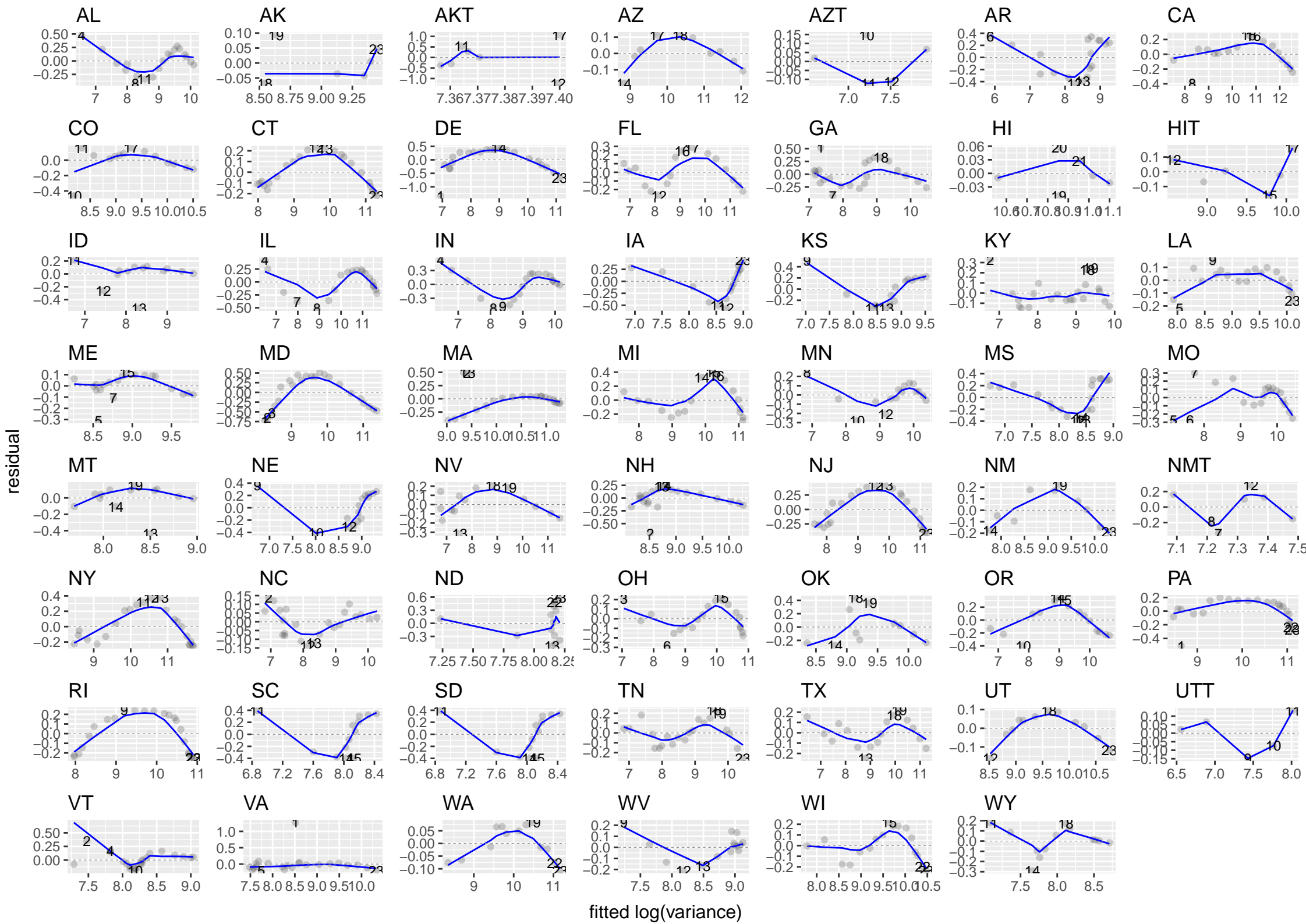

Supplement: S18 Fig — (PDF) [file pone.0226096.s019.pdf]

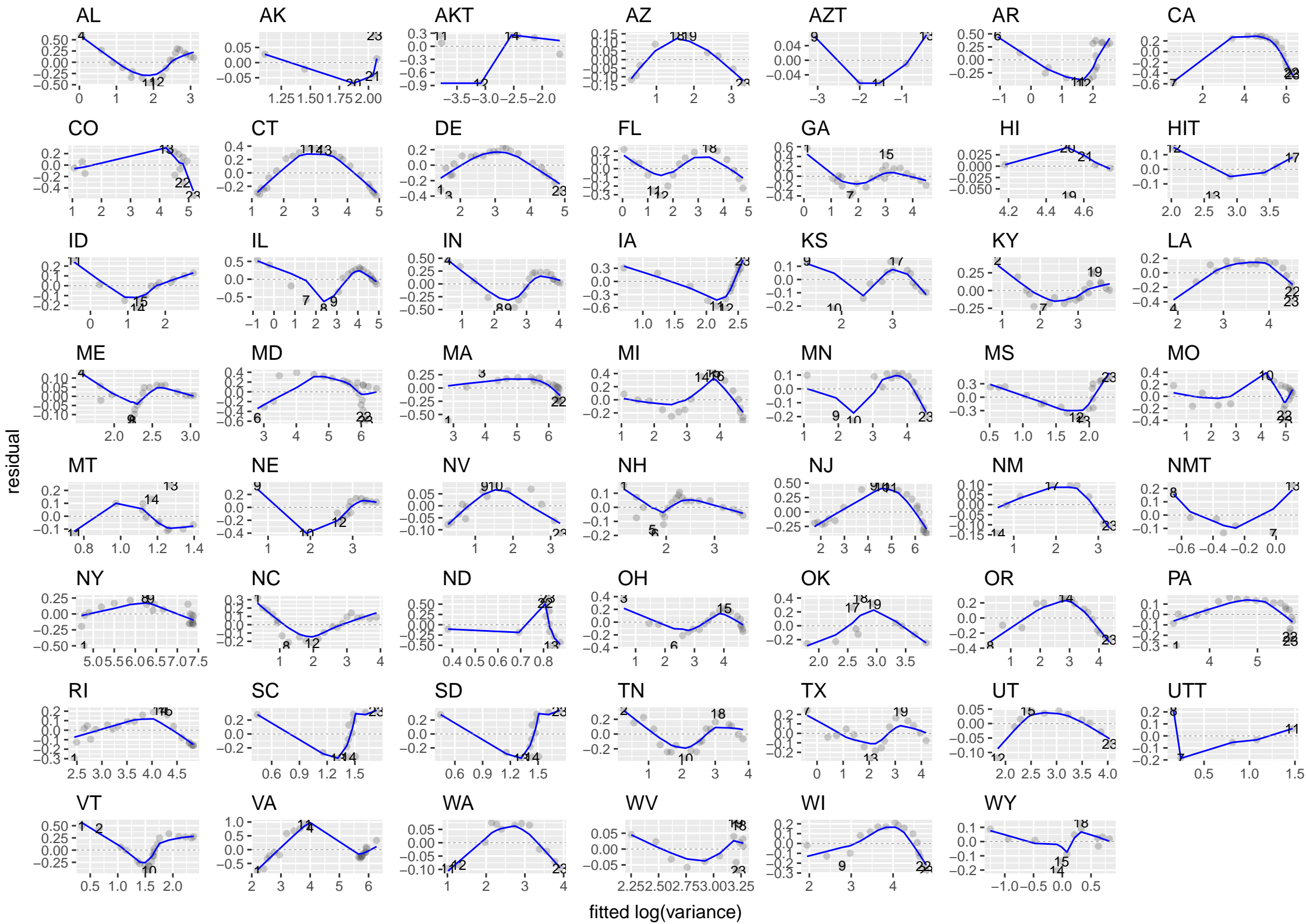

Supplement: S19 Fig — (PDF) [file pone.0226096.s020.pdf]

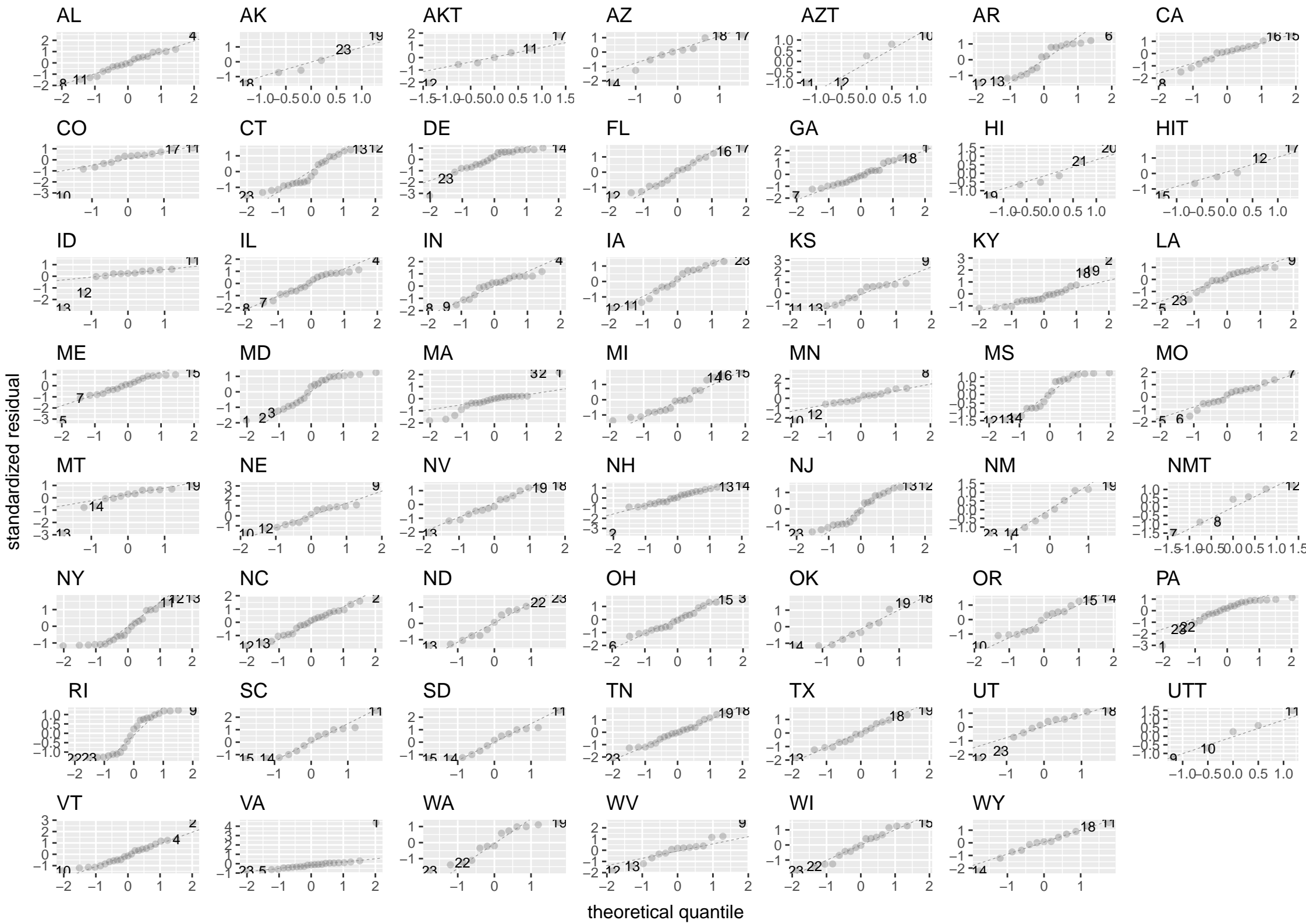

Supplement: S20 Fig — (PDF) [file pone.0226096.s021.pdf]

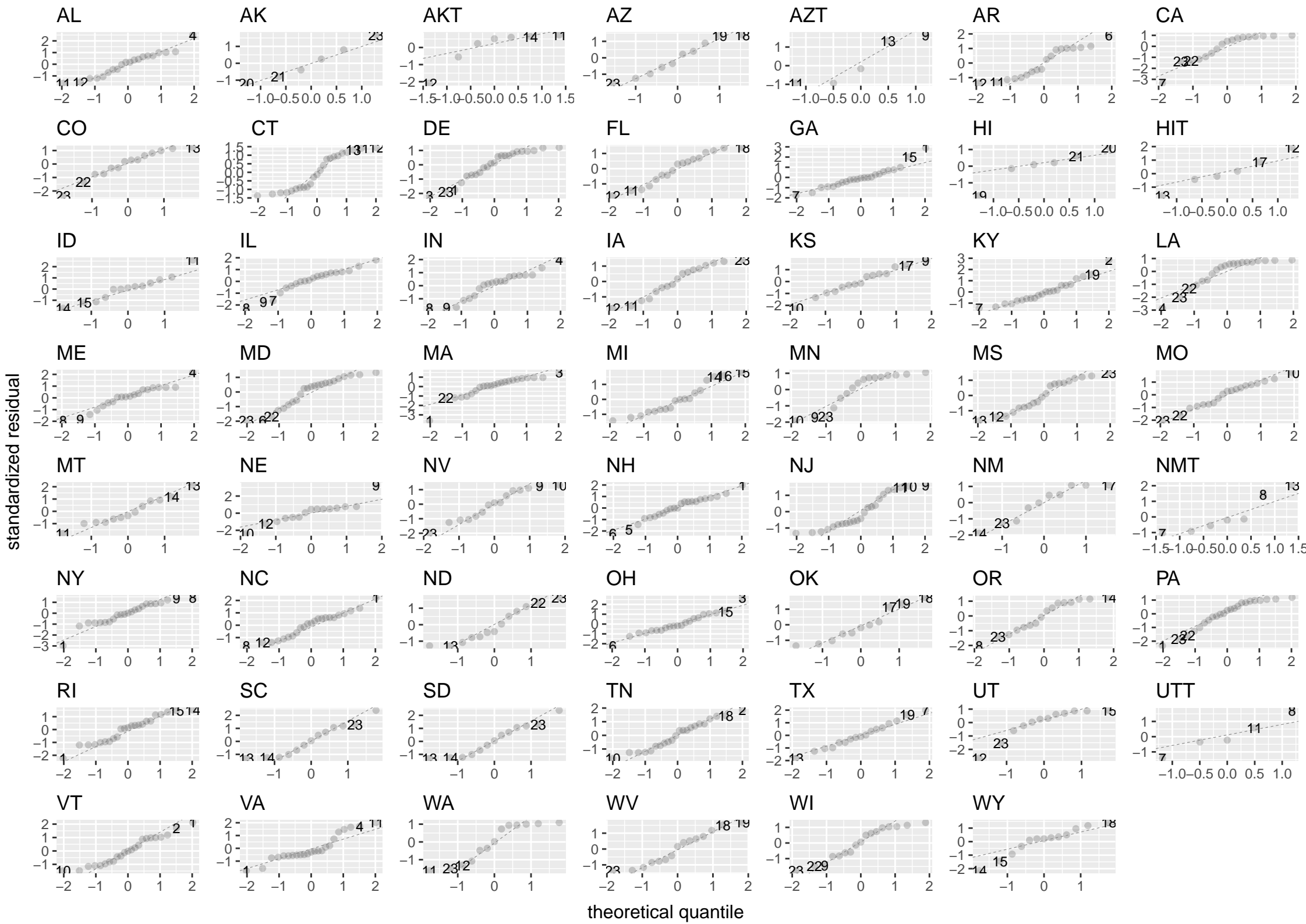

Supplement: S21 Fig — (PDF) [file pone.0226096.s022.pdf]

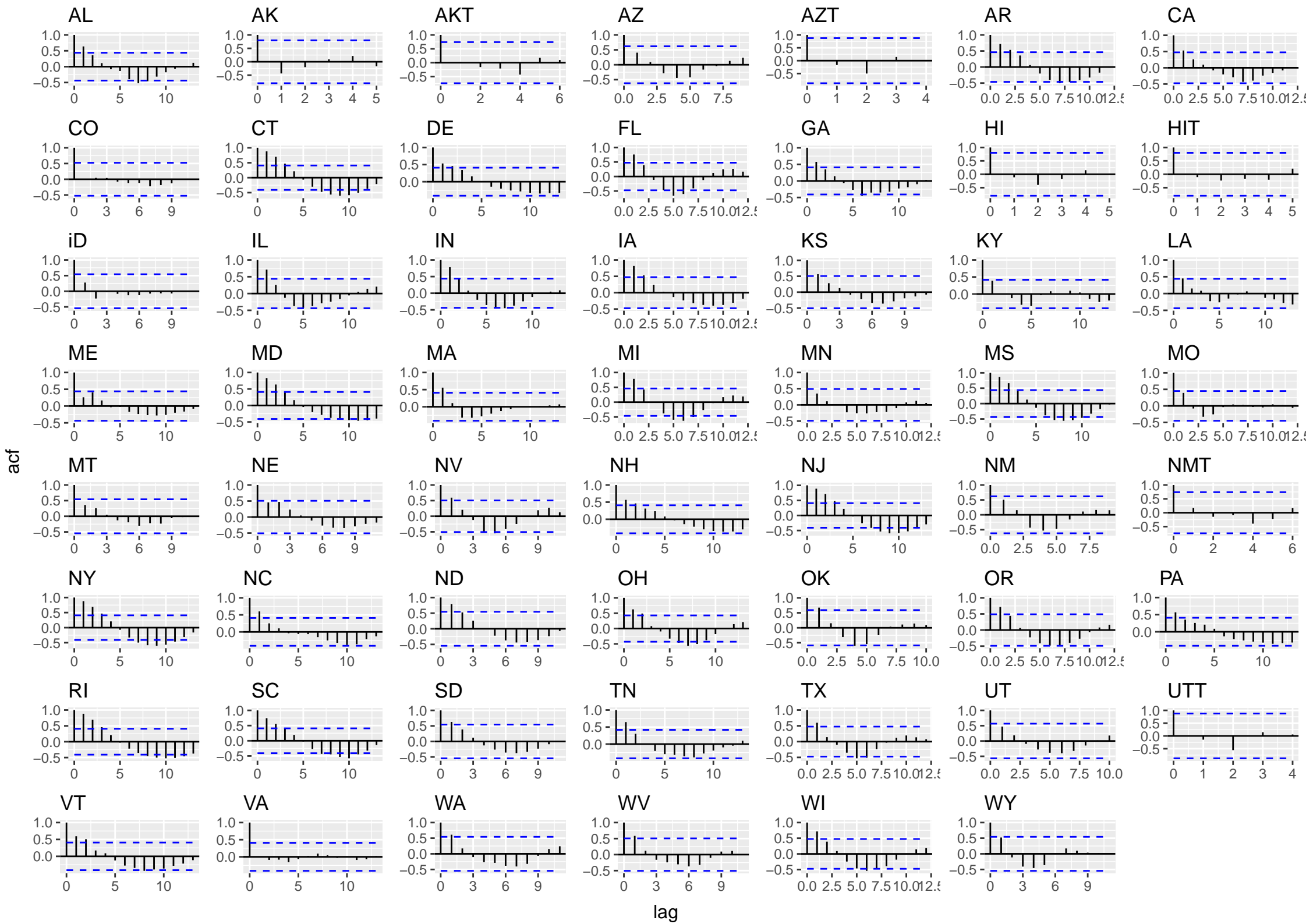

Supplement: S22 Fig — (PDF) [file pone.0226096.s023.pdf]

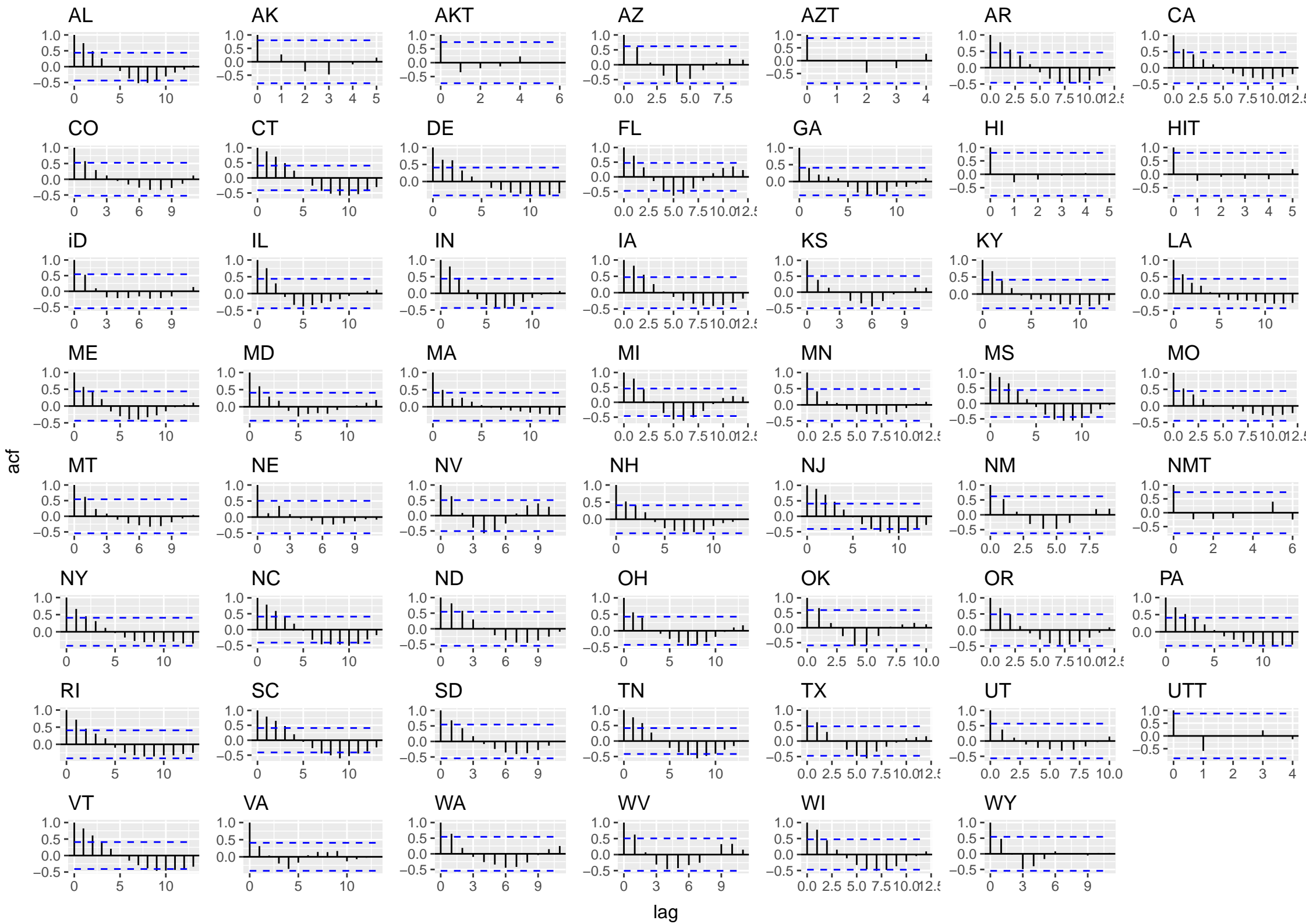

Supplement: S23 Fig — (PDF) [file pone.0226096.s024.pdf]

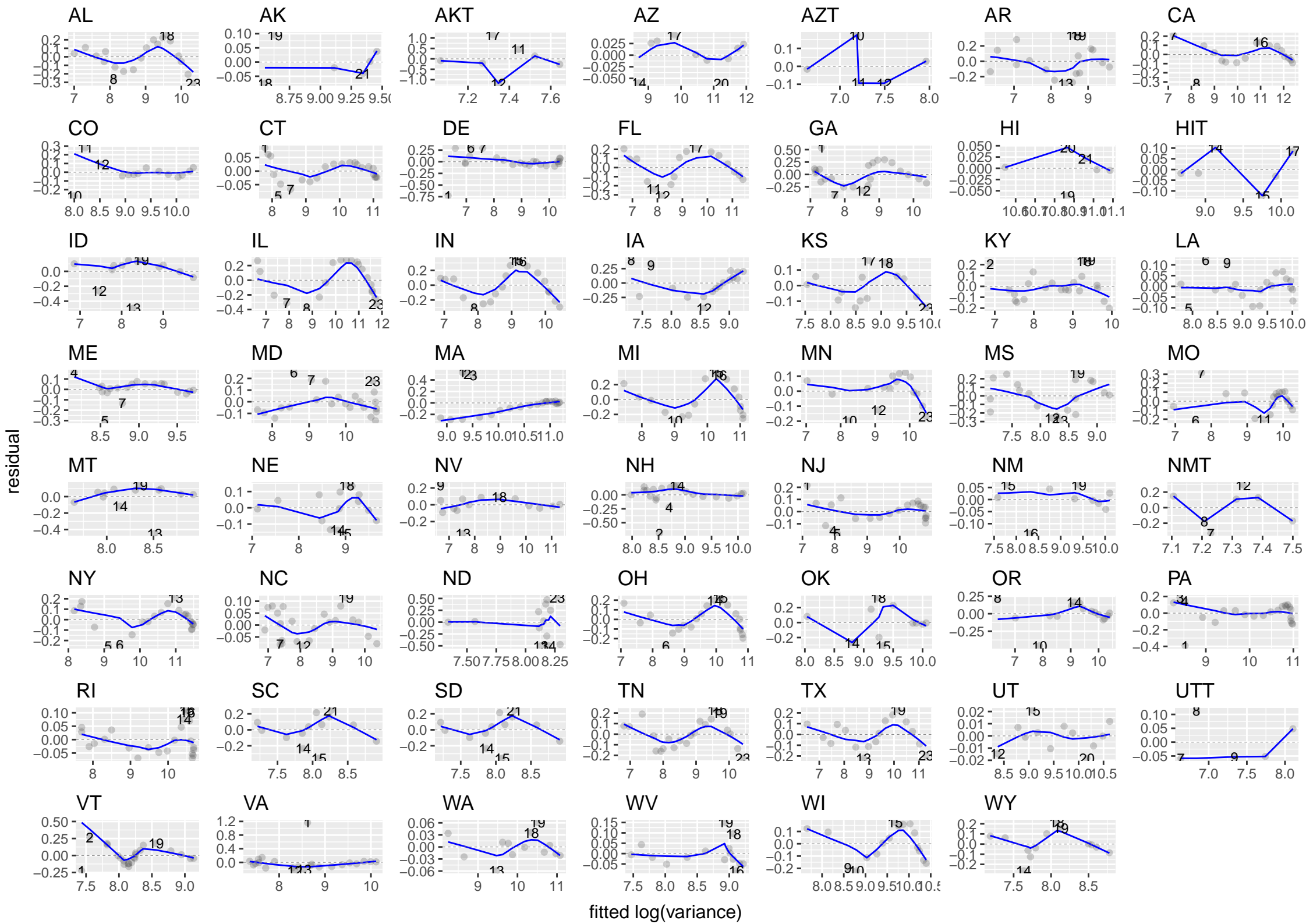

Supplement: S24 Fig — (PDF) [file pone.0226096.s025.pdf]

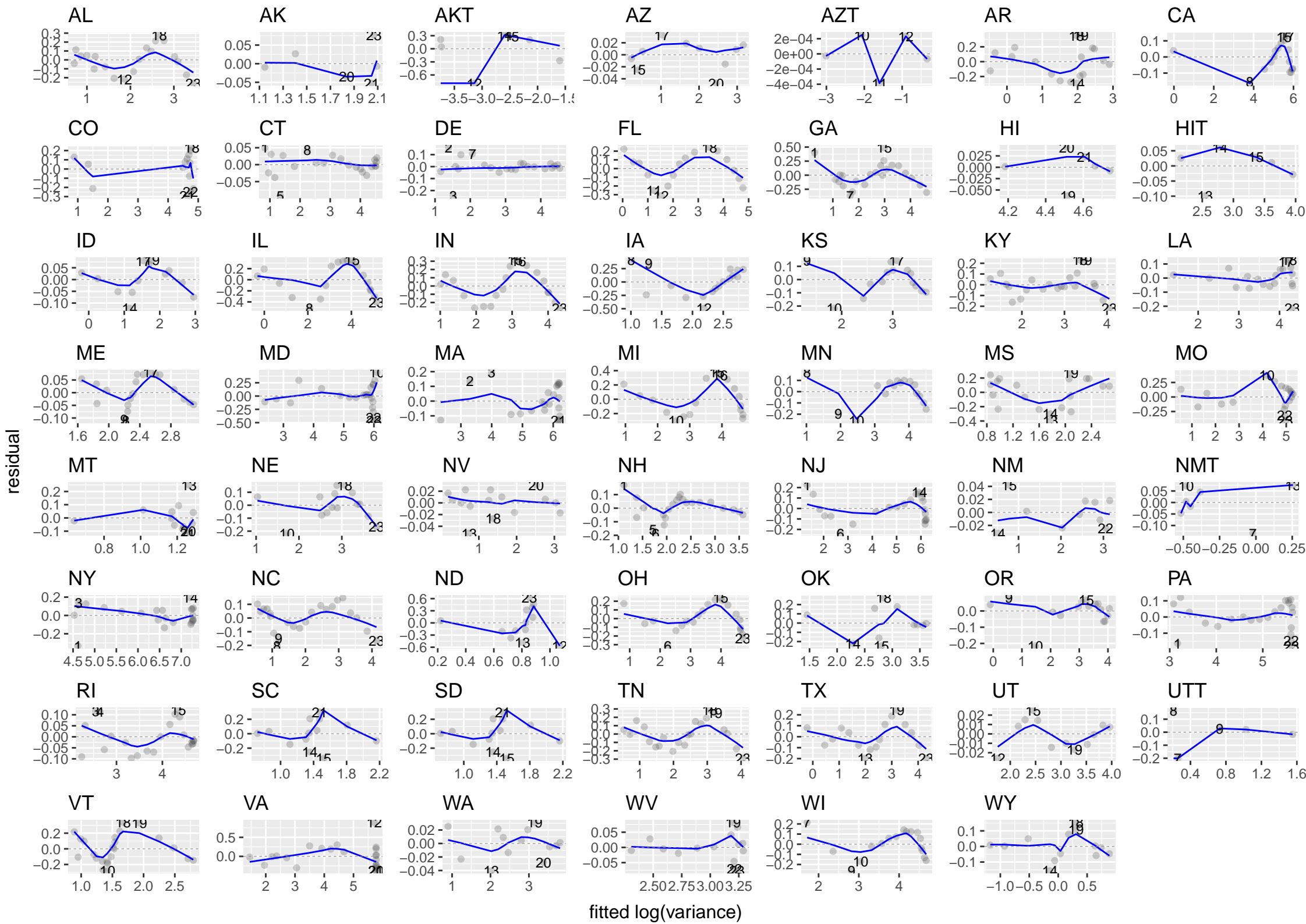

Supplement: S25 Fig — (PDF) [file pone.0226096.s026.pdf]

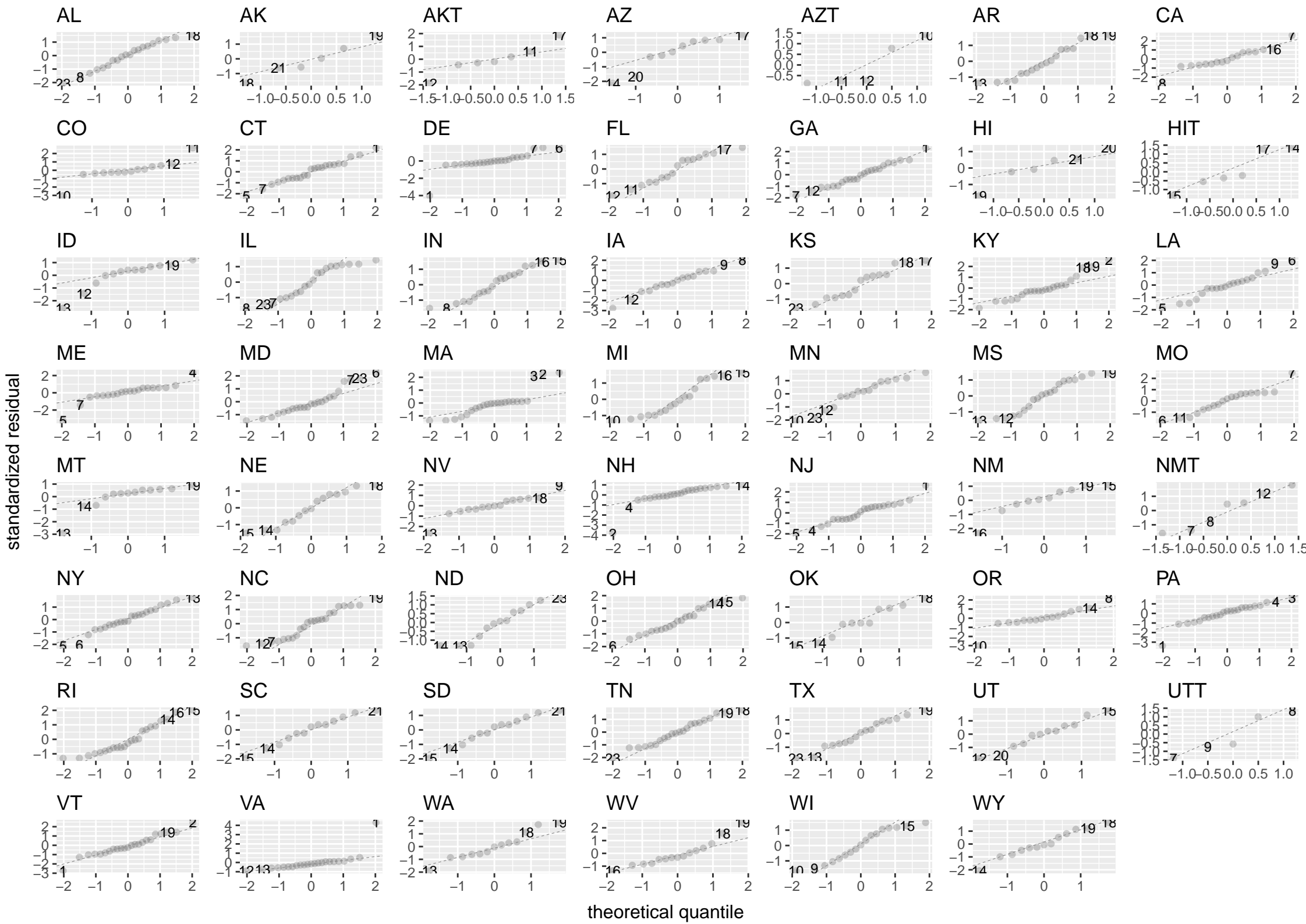

Supplement: S26 Fig — (PDF) [file pone.0226096.s027.pdf]

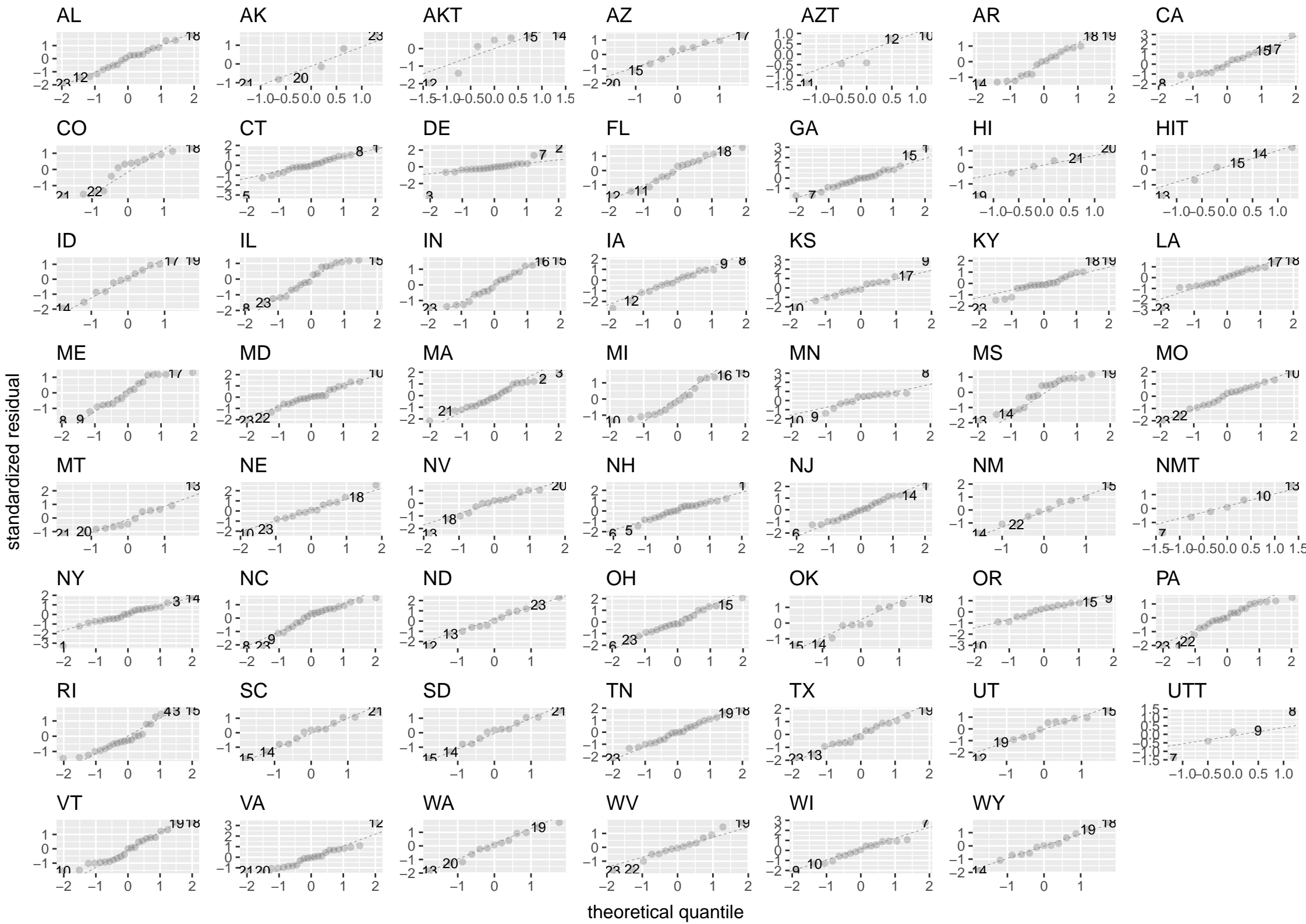

Supplement: S27 Fig — (PDF) [file pone.0226096.s028.pdf]

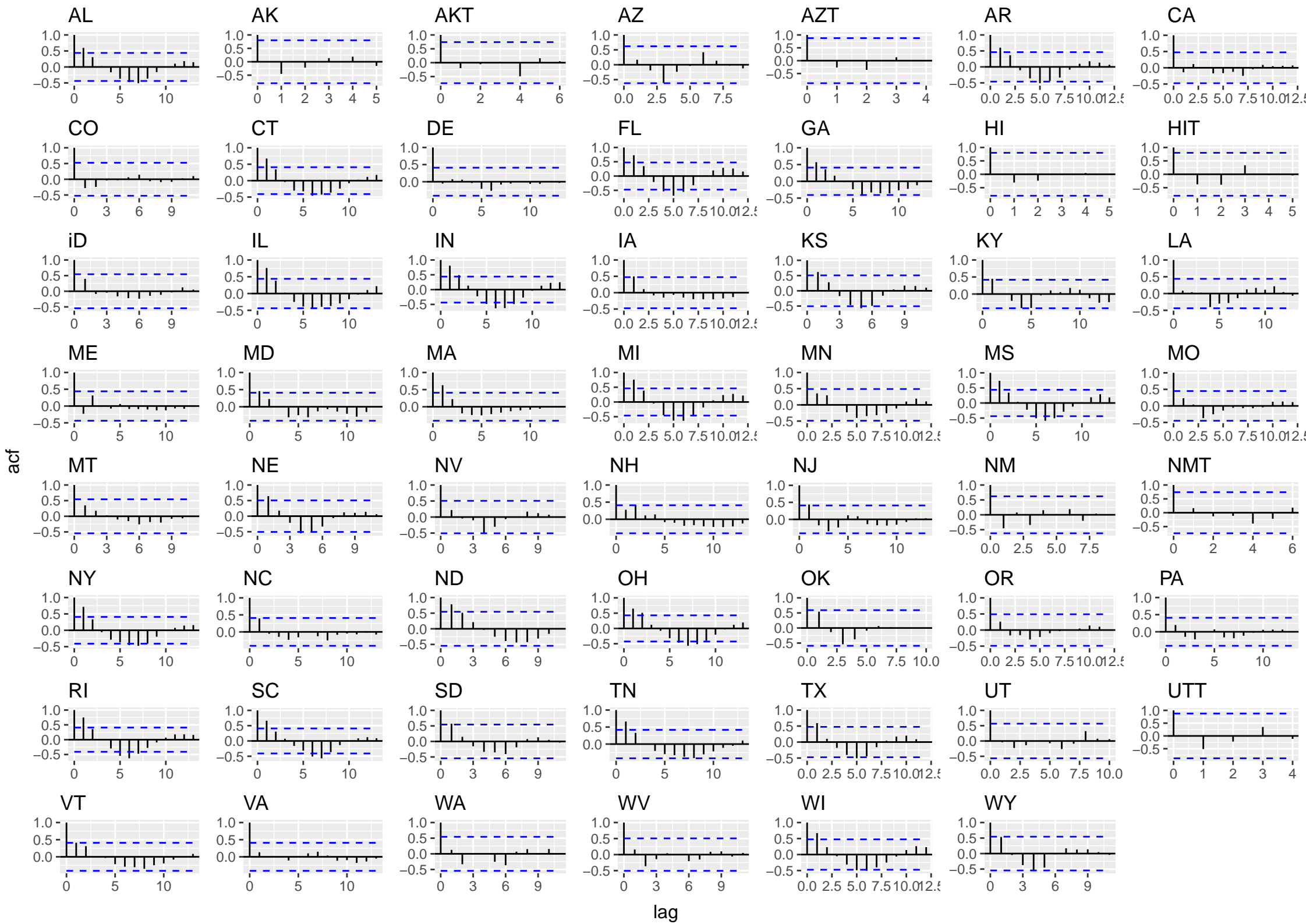

Supplement: S28 Fig — (PDF) [file pone.0226096.s029.pdf]

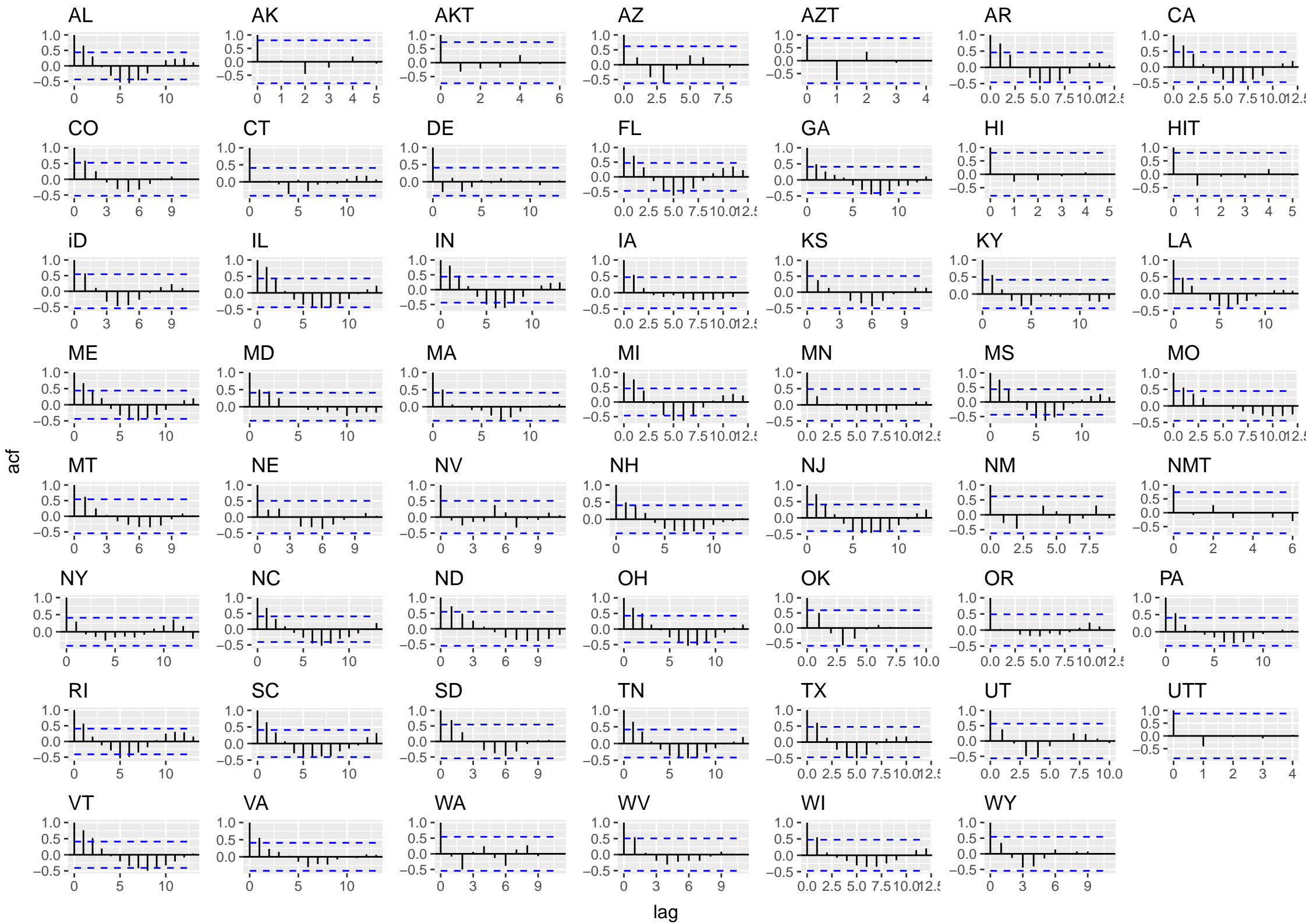

Supplement: S29 Fig — (PDF) [file pone.0226096.s030.pdf]

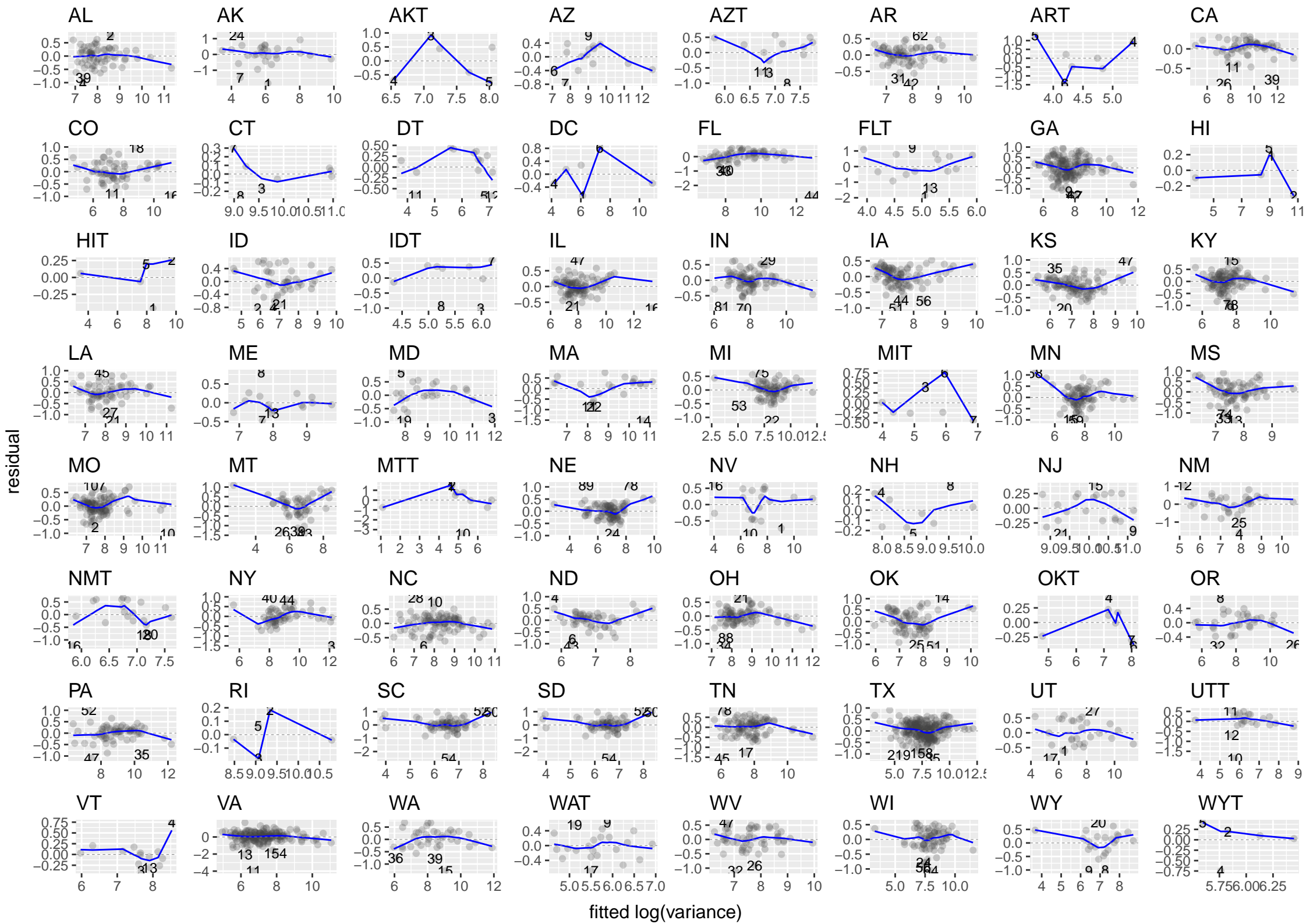

Supplement: S30 Fig — (PDF) [file pone.0226096.s031.pdf]

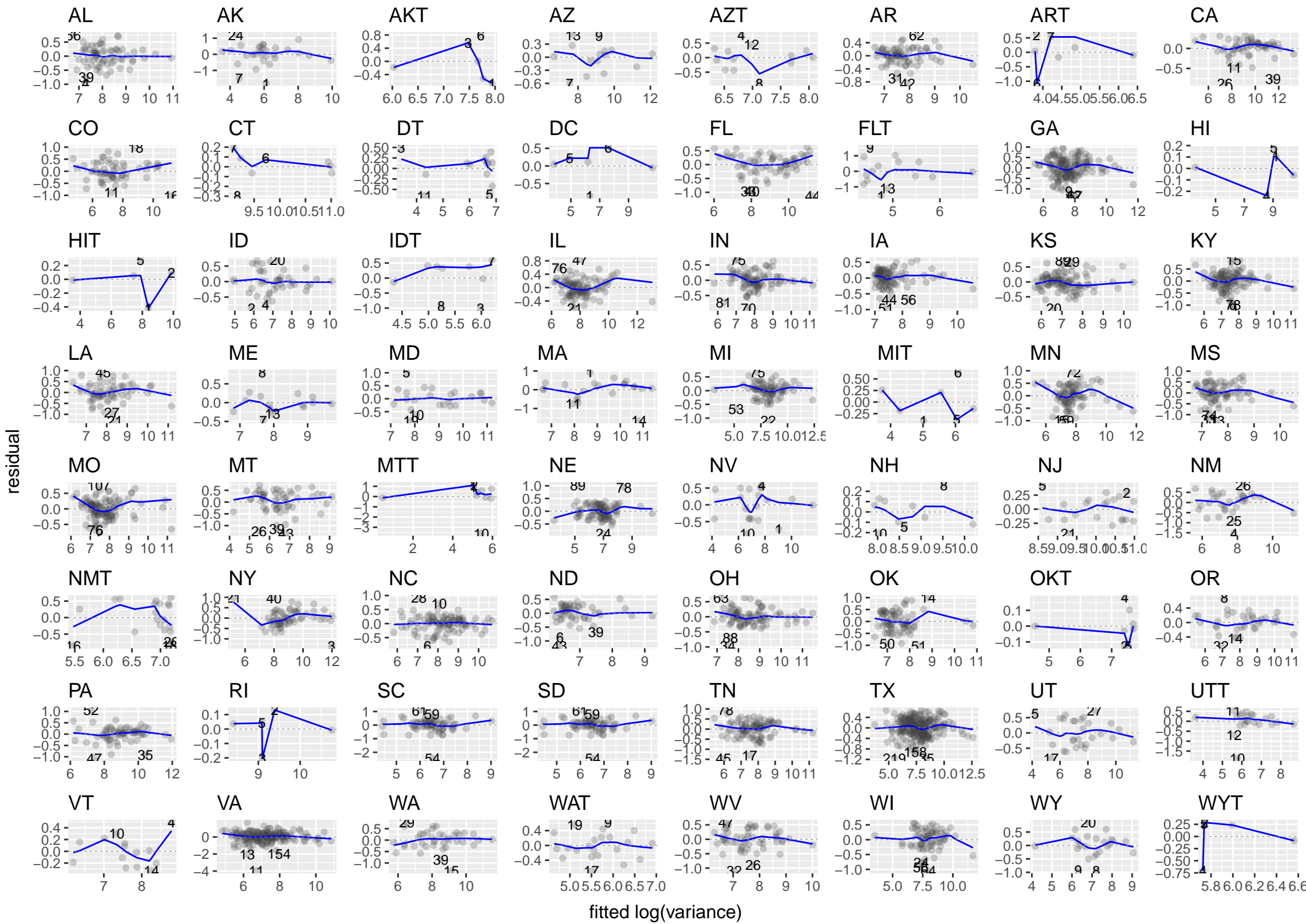

Supplement: S31 Fig — (PDF) [file pone.0226096.s032.pdf]

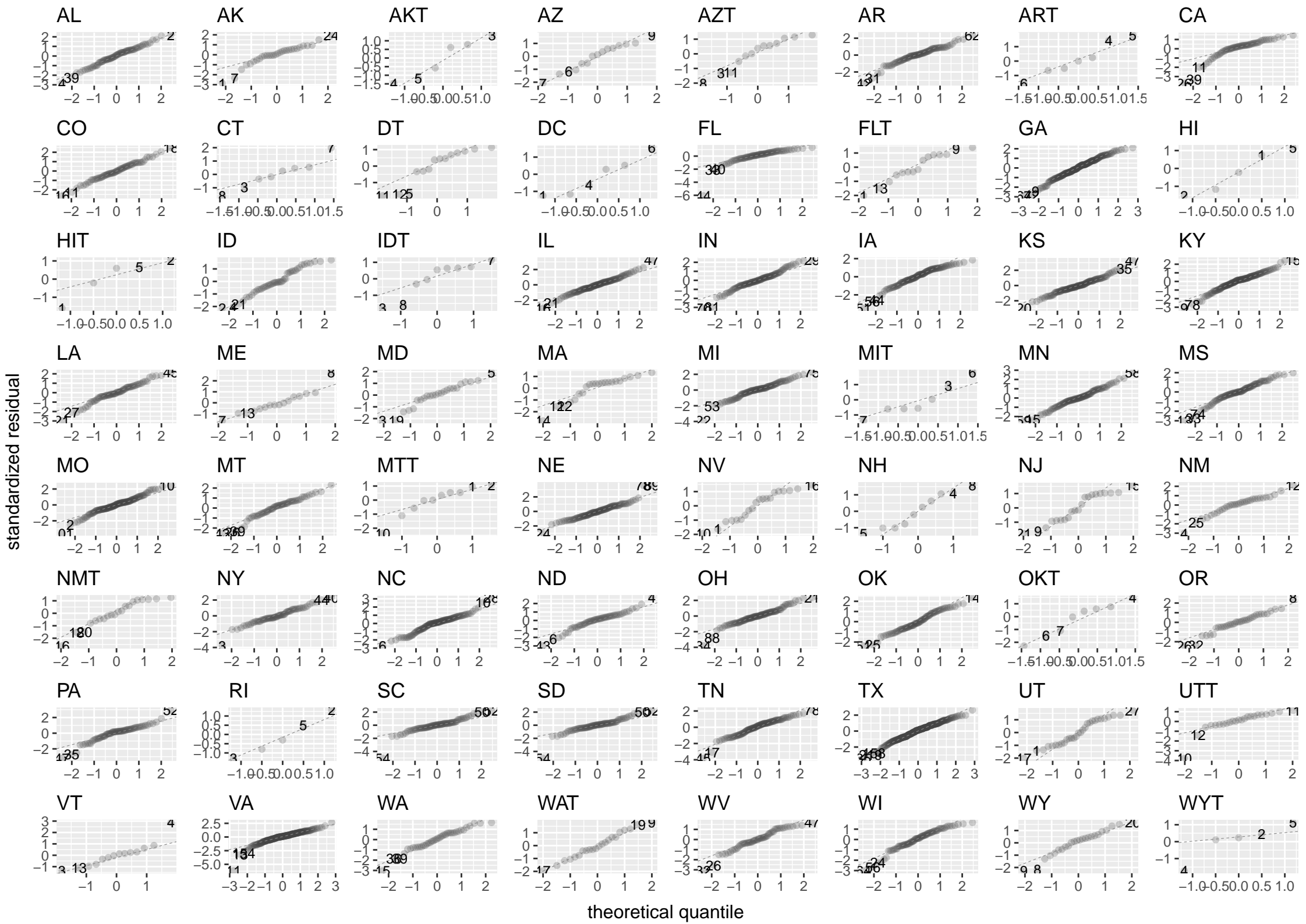

Supplement: S32 Fig — (PDF) [file pone.0226096.s033.pdf]

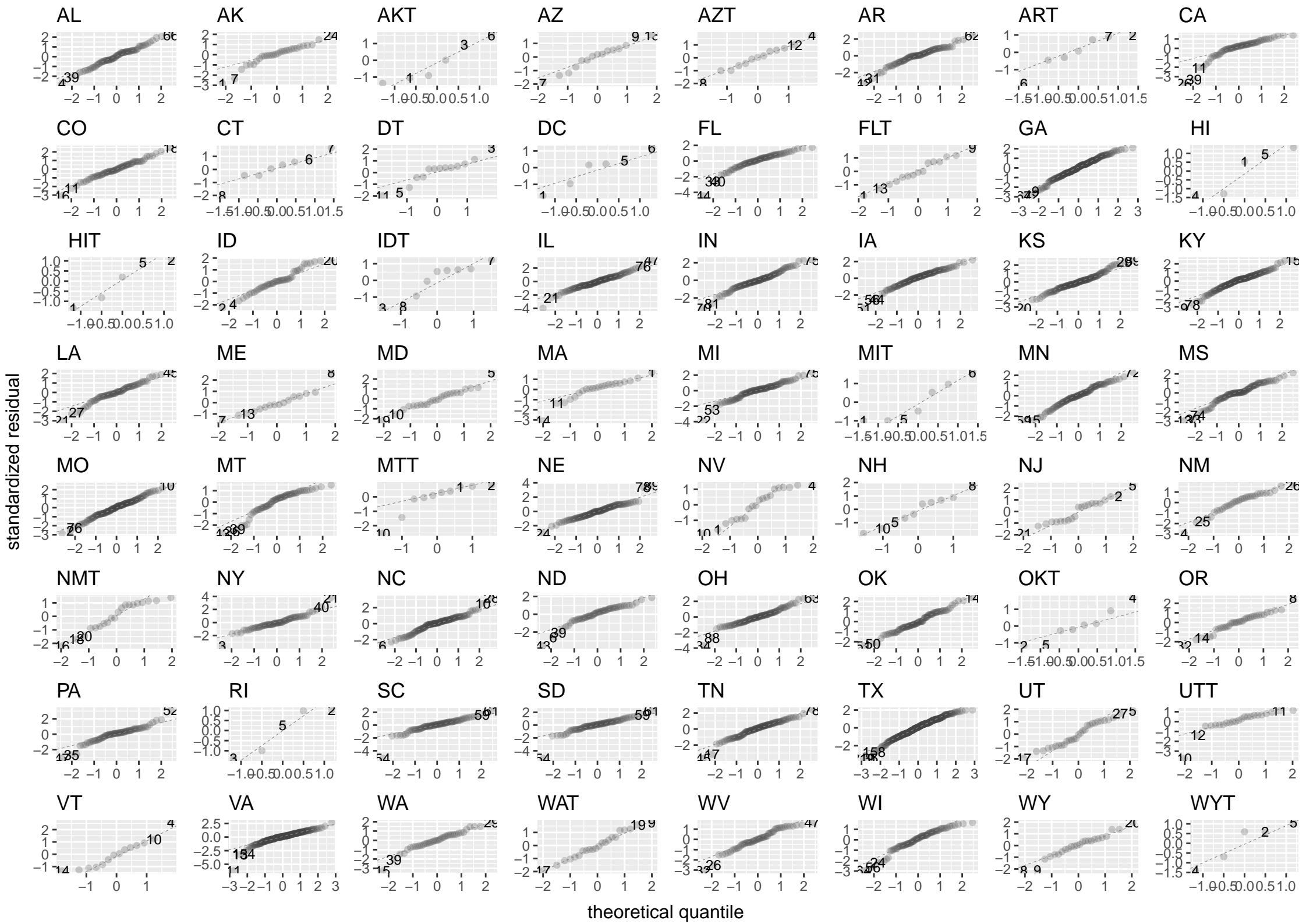

Supplement: S33 Fig — (PDF) [file pone.0226096.s034.pdf]

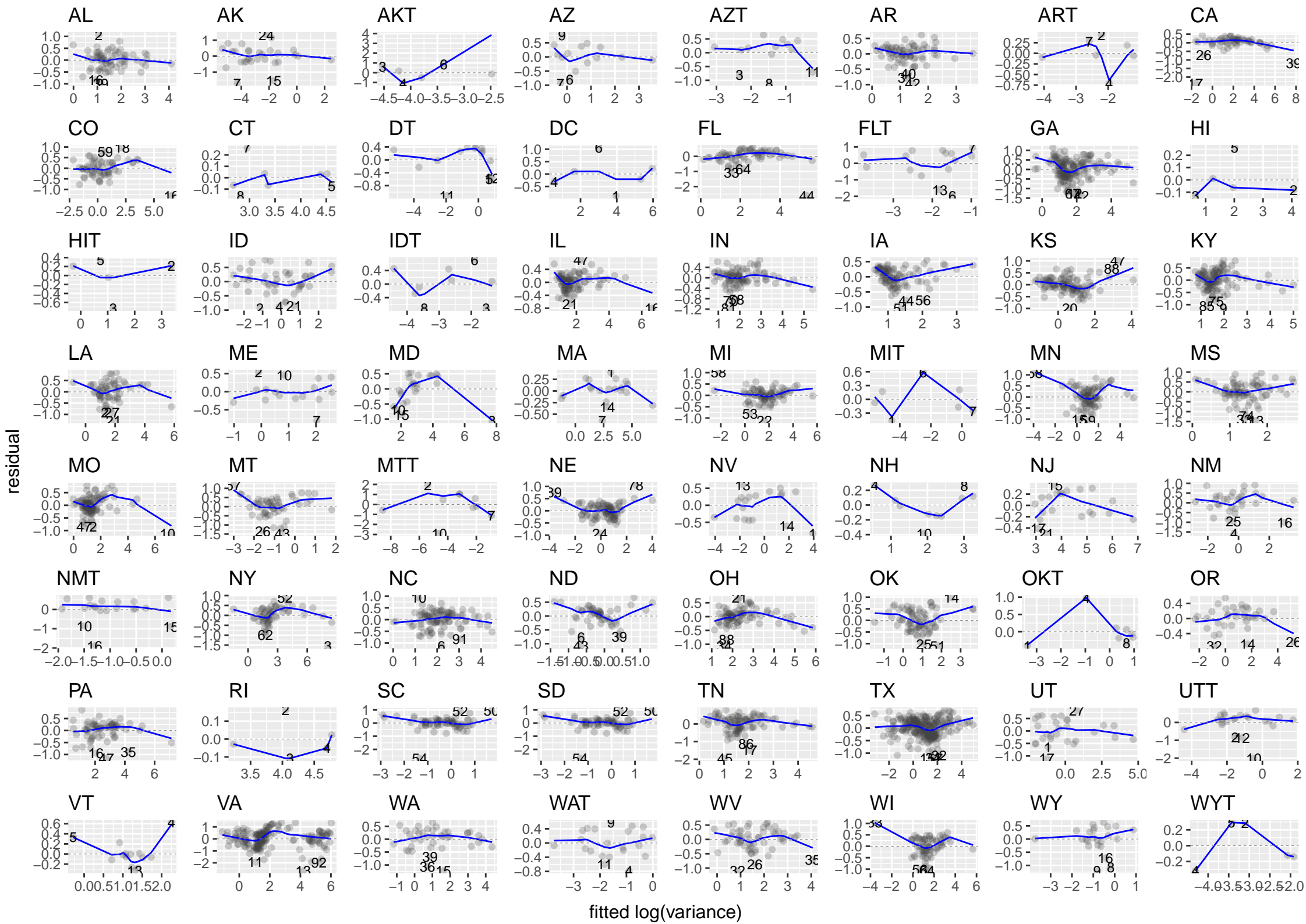

Supplement: S34 Fig — (PDF) [file pone.0226096.s035.pdf]

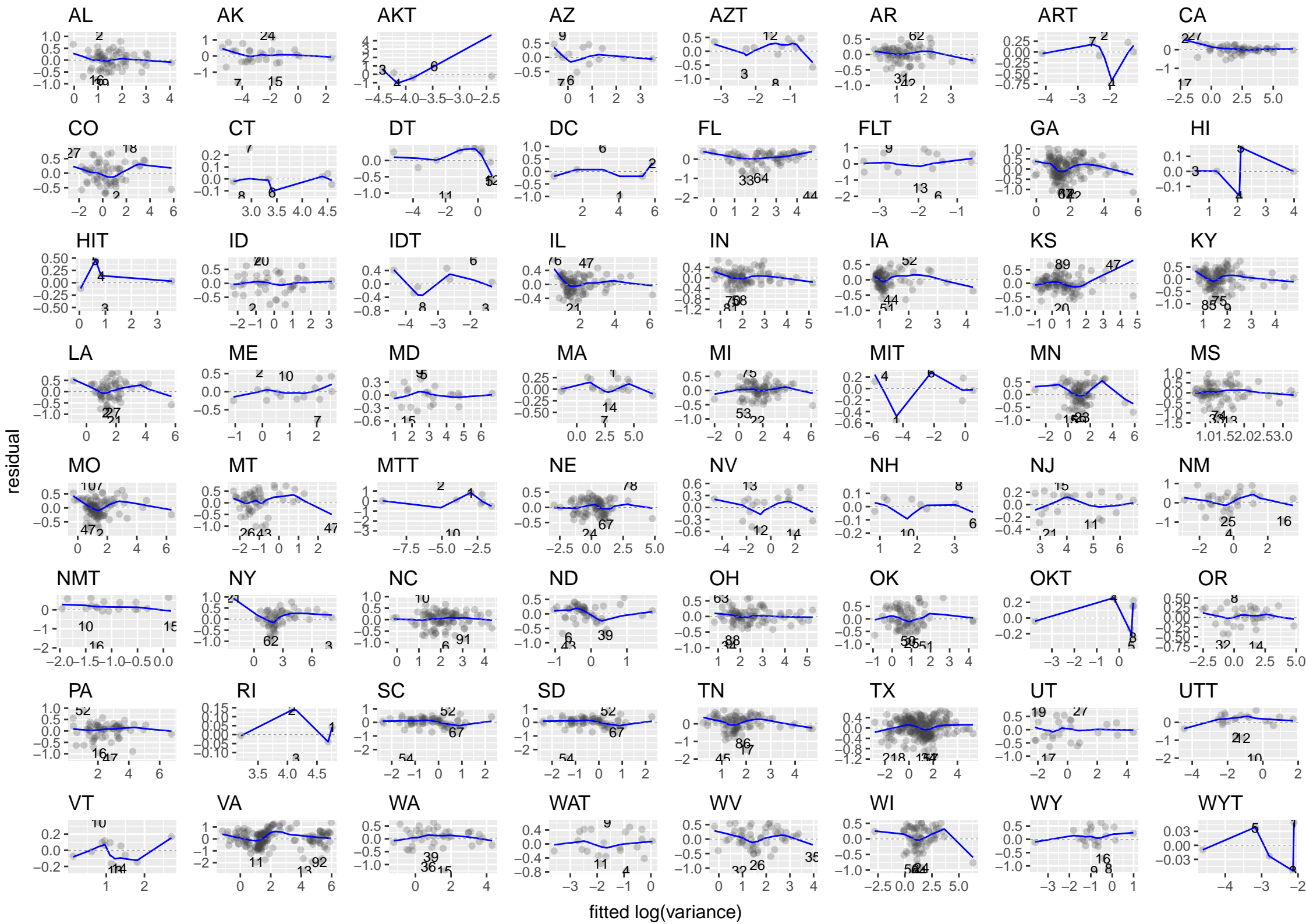

Supplement: S35 Fig — (PDF) [file pone.0226096.s036.pdf]

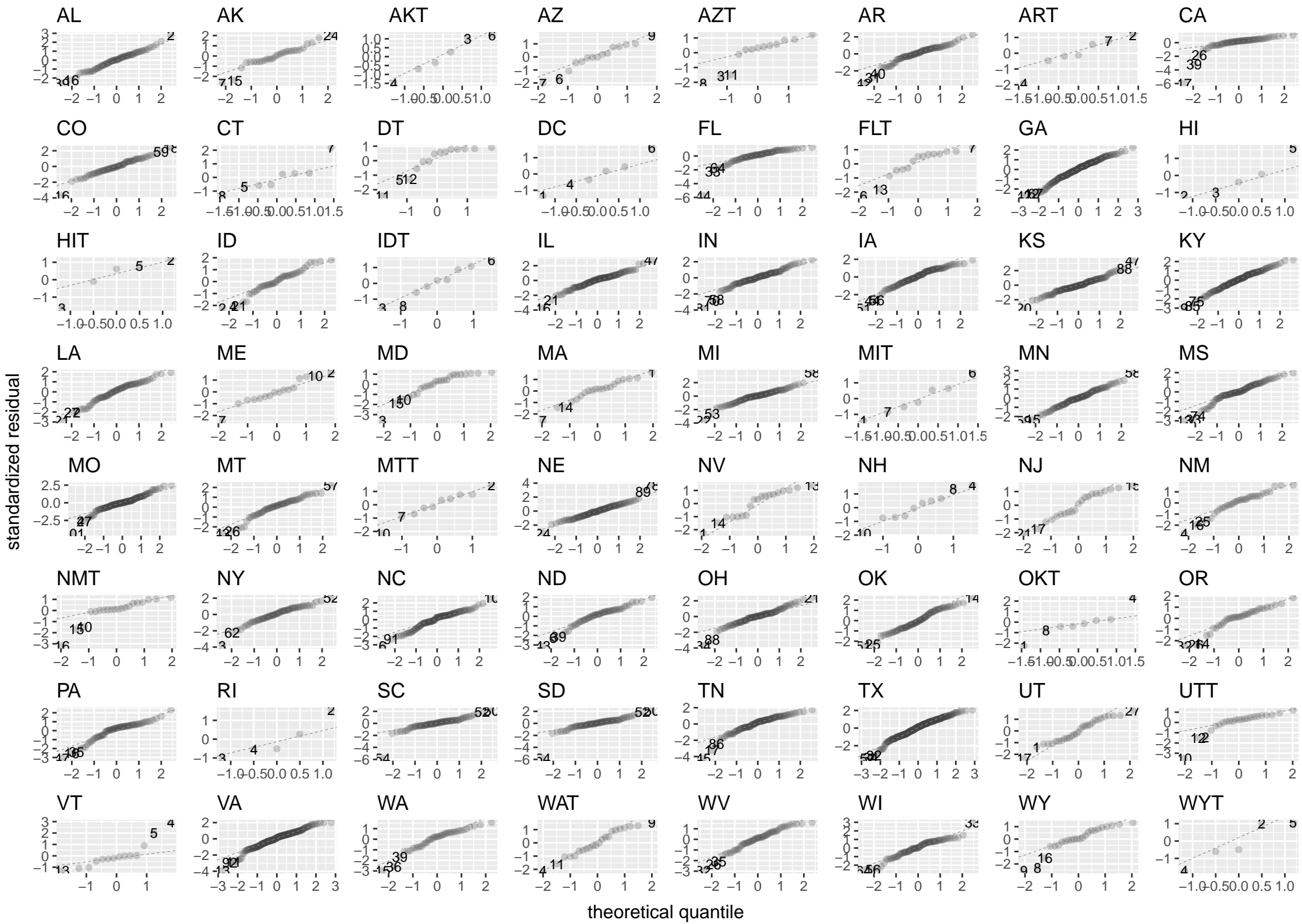

Supplement: S36 Fig — (PDF) [file pone.0226096.s037.pdf]

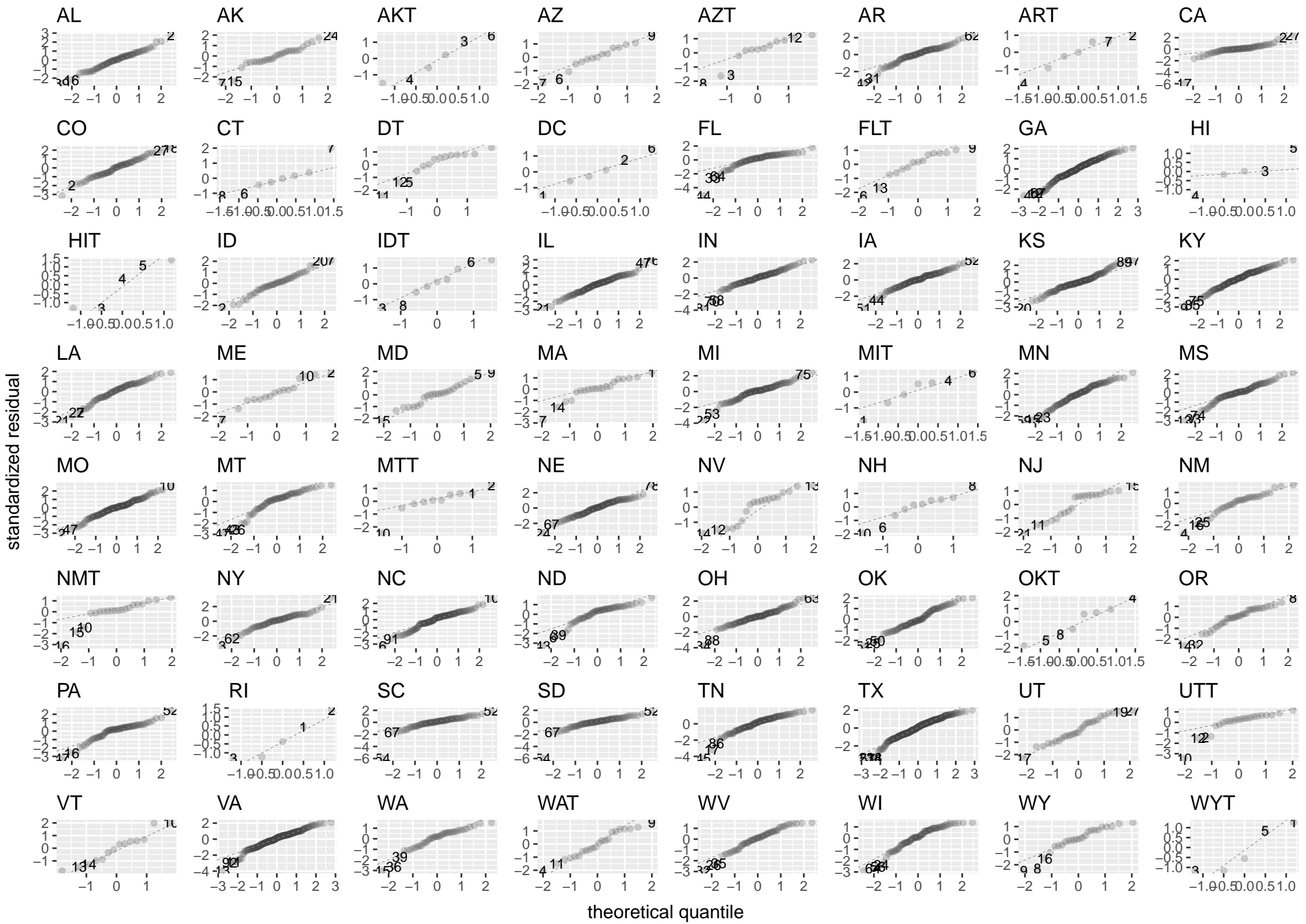

Supplement: S37 Fig — (PDF) [file pone.0226096.s038.pdf]

spatial variance of county population count

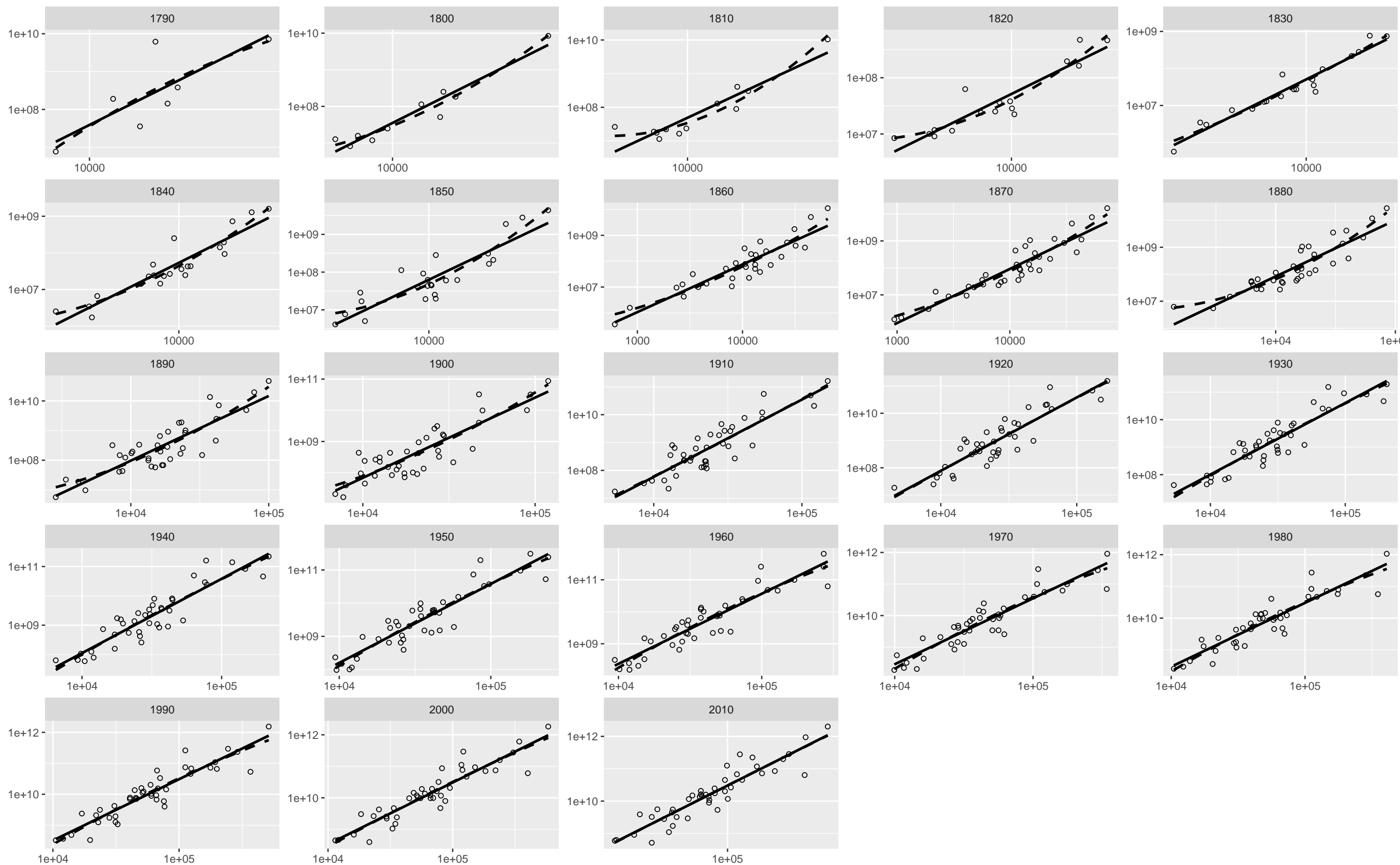

spatial mean of county population count

Supplement: S38 Fig — (PDF) [file pone.0226096.s039.pdf]

spatial variance of county population density

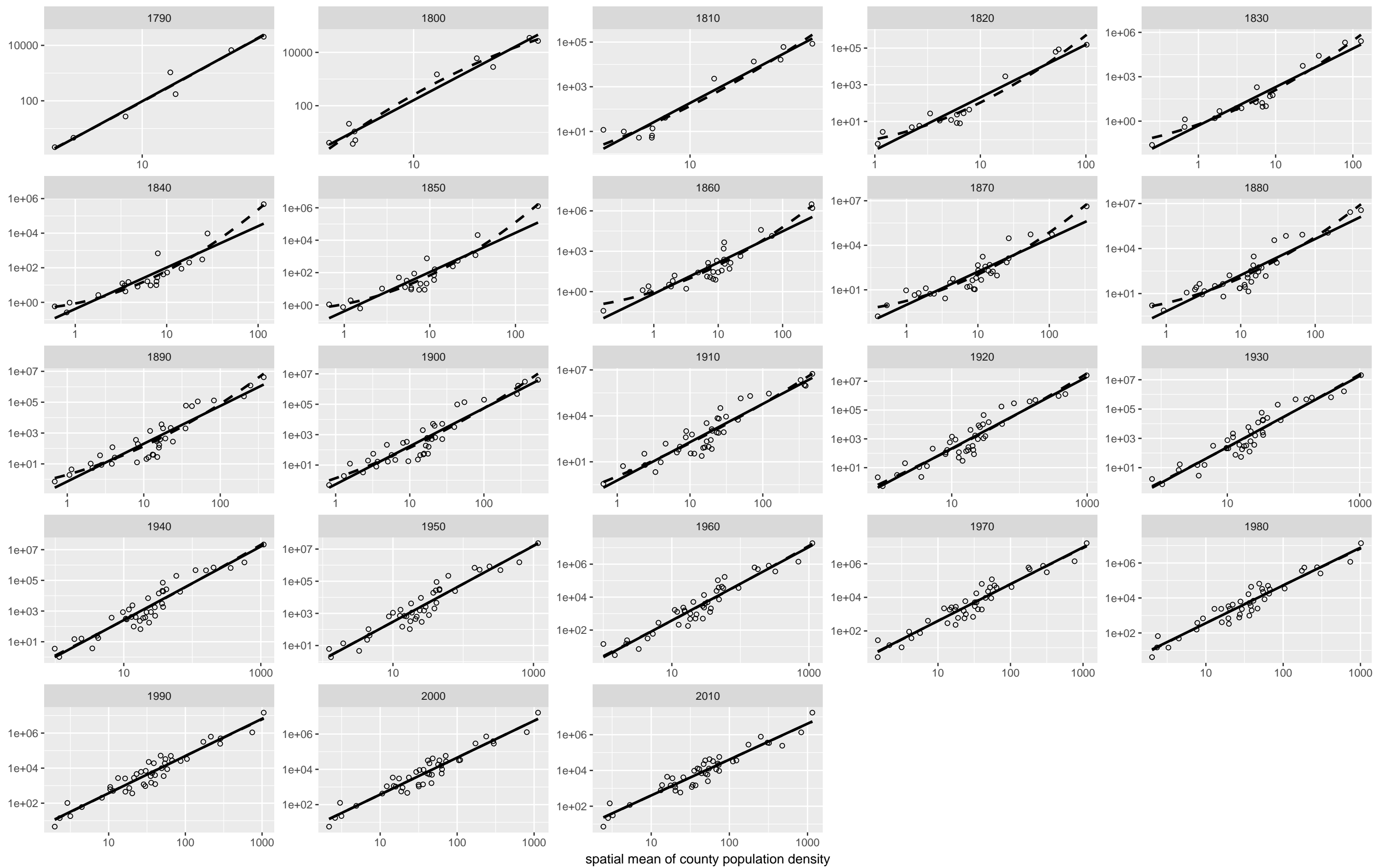

Supplement: S39 Fig — (PDF) [file pone.0226096.s040.pdf]

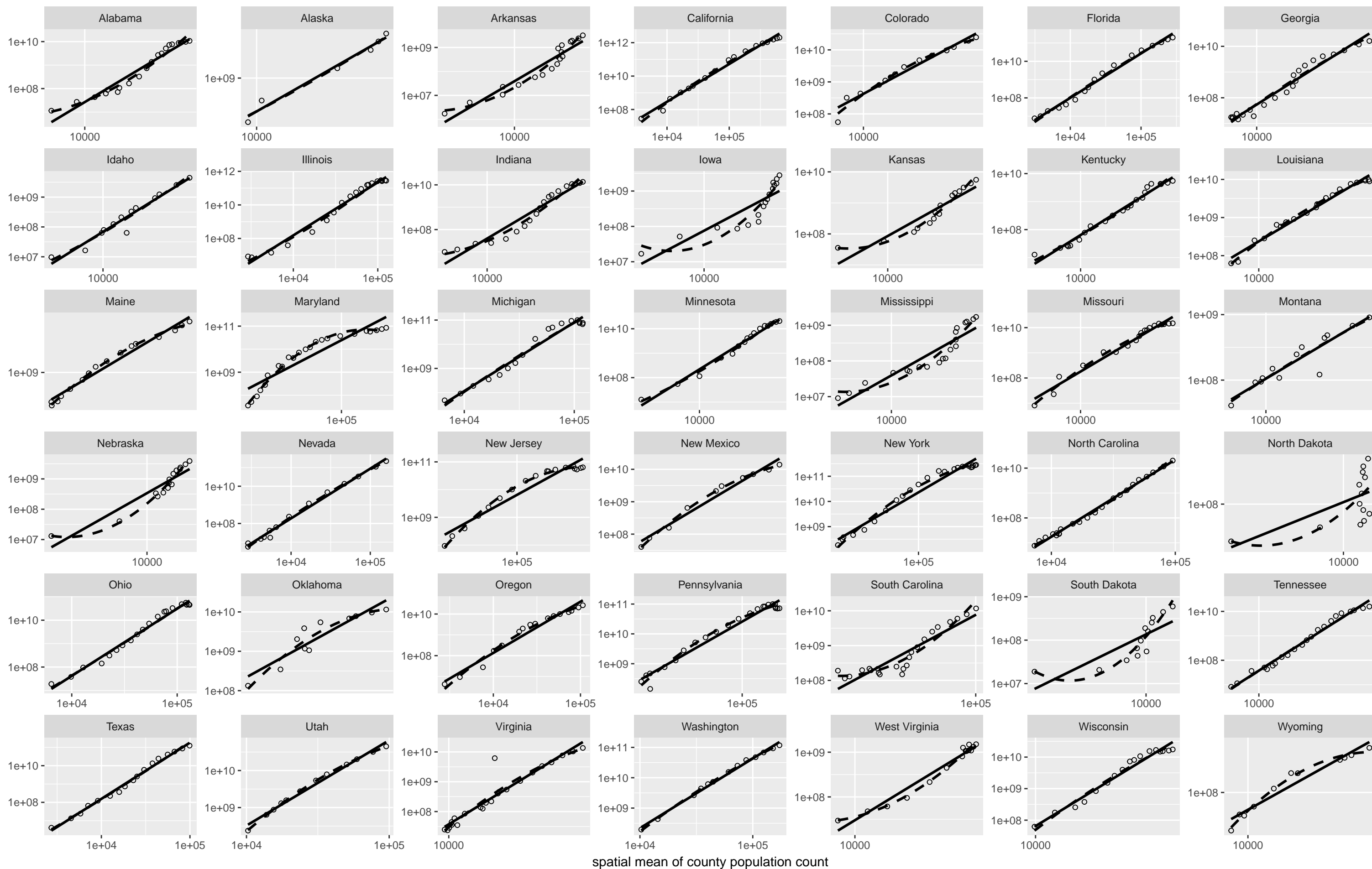

Supplement: S40 Fig — (PDF) [file pone.0226096.s041.pdf]

spatial variance of county population density

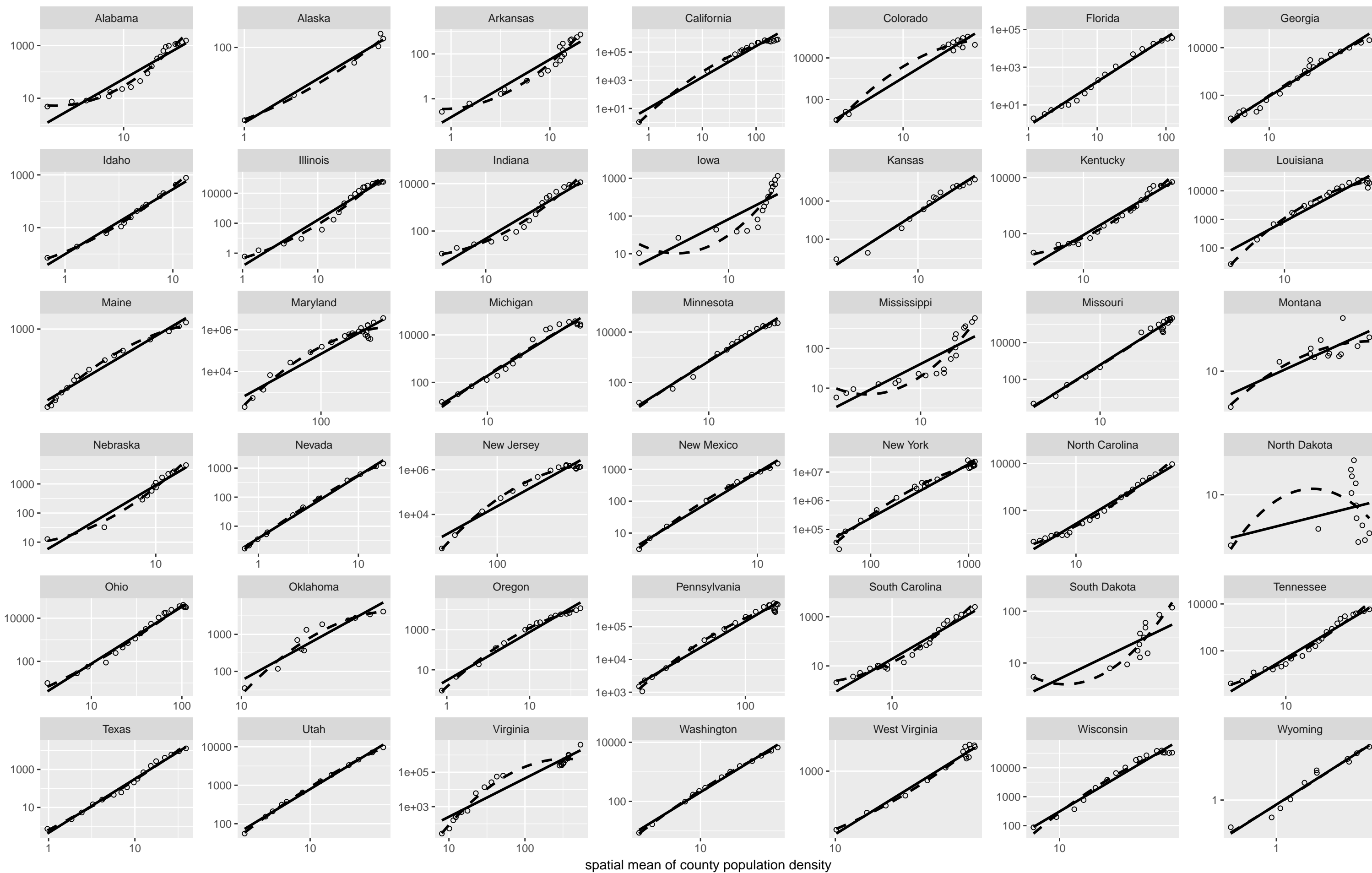

spatial mean of county population density

Supplement: S41 Fig — (PDF) [file pone.0226096.s042.pdf]

temporal variance of county population density

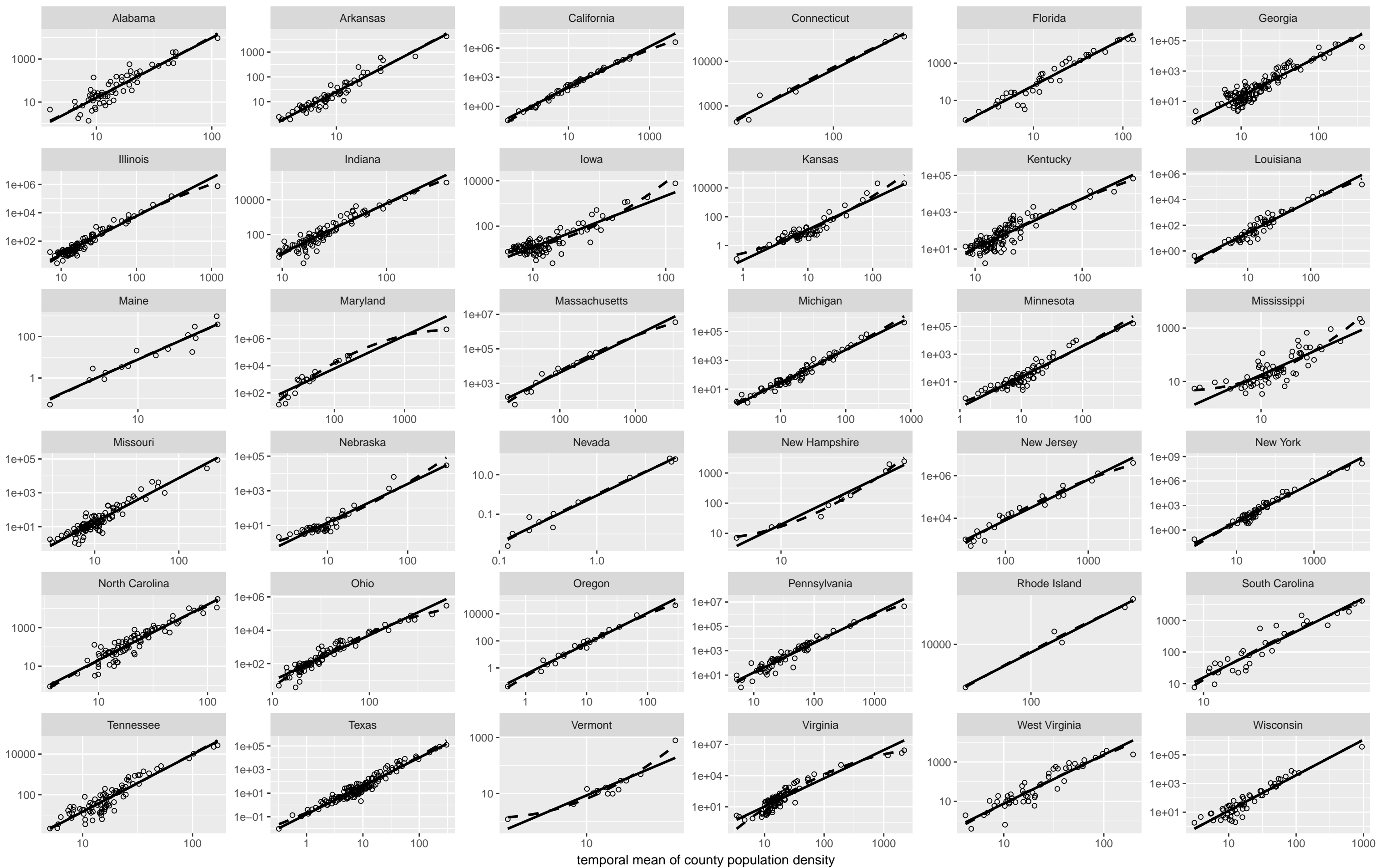

Supplement: S43 Fig — (PDF) [file pone.0226096.s044.pdf]

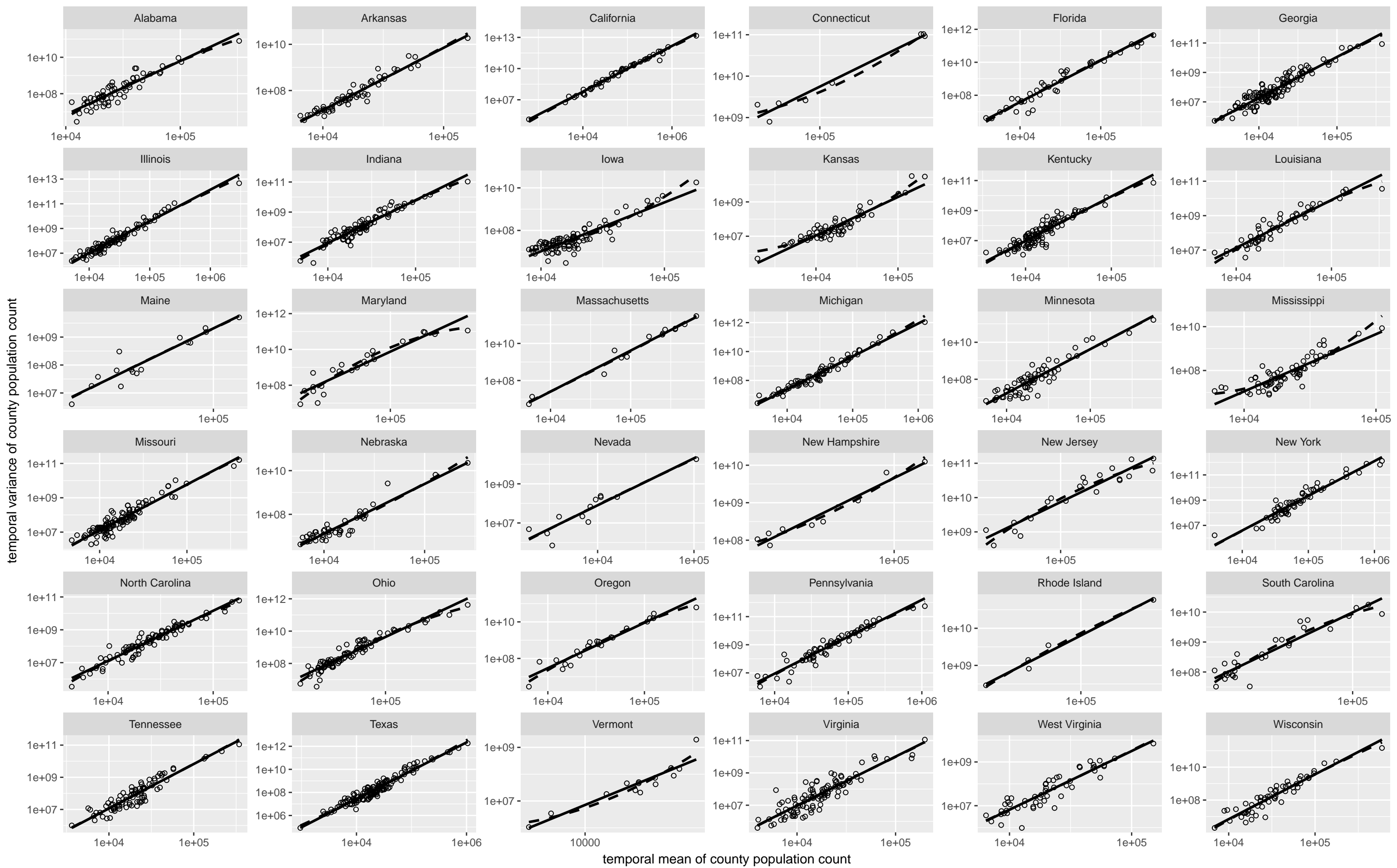

Supplement: S44 Fig — (PDF) [file pone.0226096.s045.pdf]

**(a)**

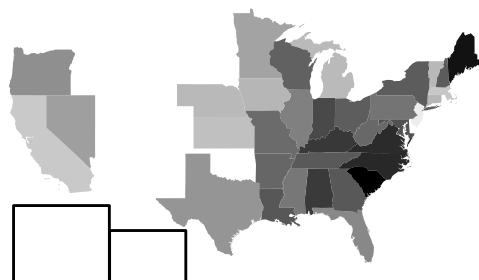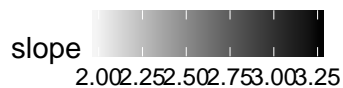

**(b)**

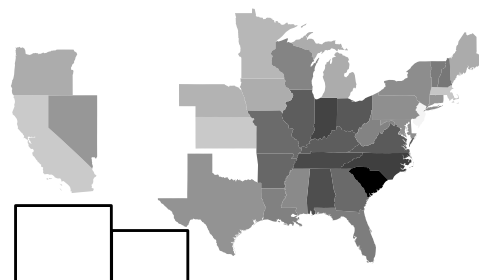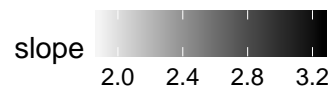

**(c)**

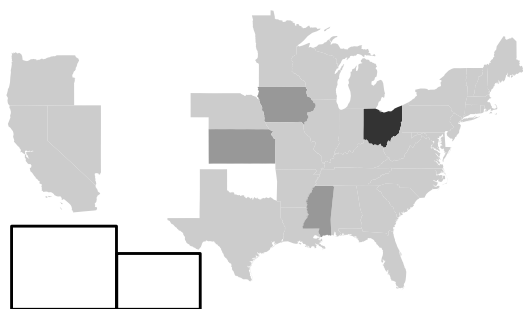

quadratic coefficient

minus plus zero

**(d)**

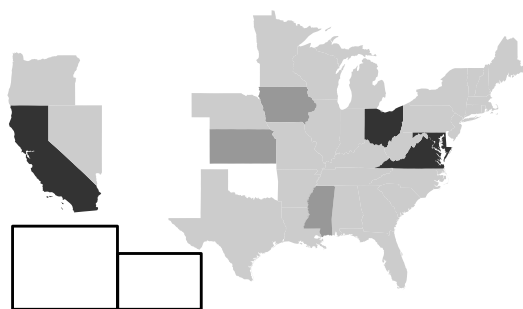

quadratic coefficient

minus plus zero

Supplement: S45 Fig — Point estimates of the slope ((a) and (b)) of ols linear regressions and the sign of the quadratic coefficient ((c) and (d)) of ols quadratic regressions for spatial TL for each state, using count ((a) and (c)) and density ((b) and (d)). (PDF) [file pone.0226096.s046.pdf]

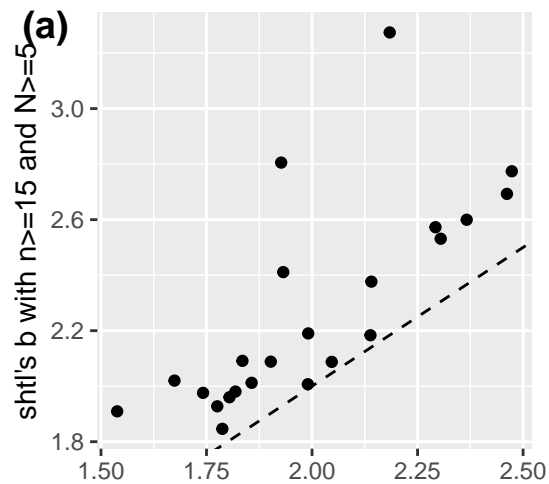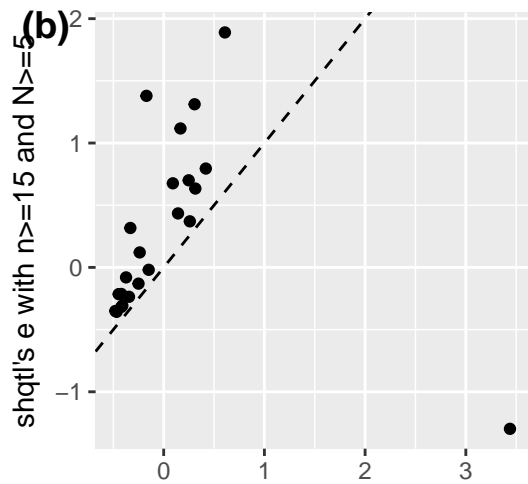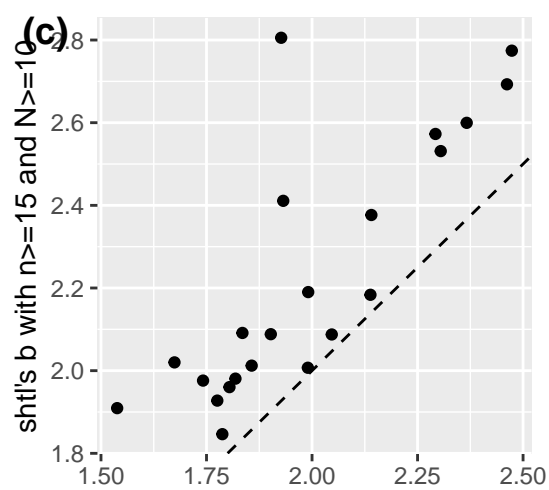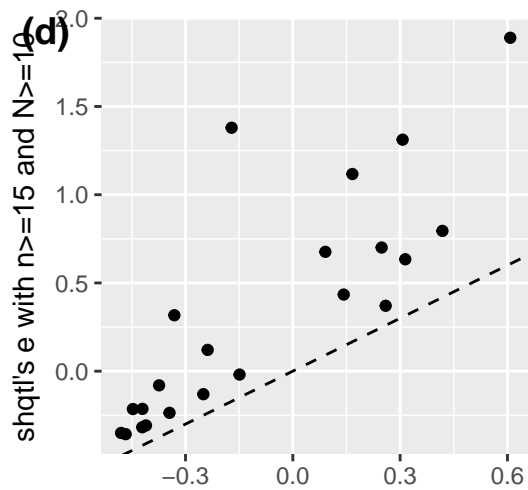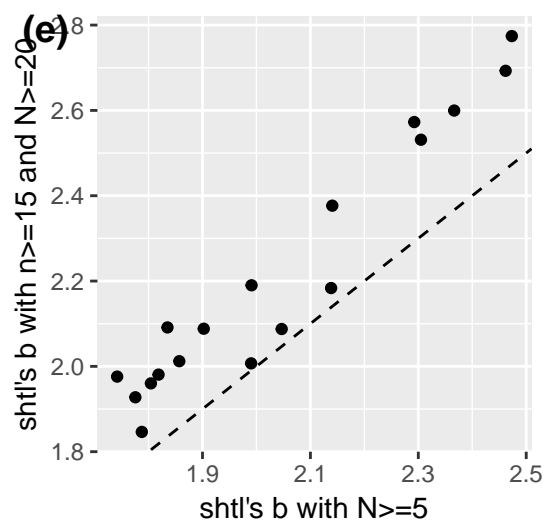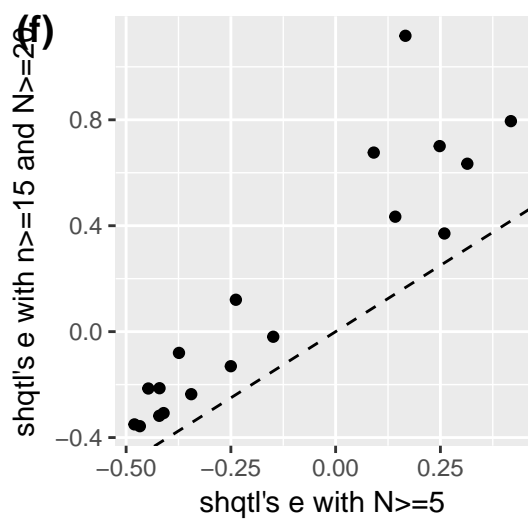

Supplement: S46 Fig — (PDF) [file pone.0226096.s047.pdf]

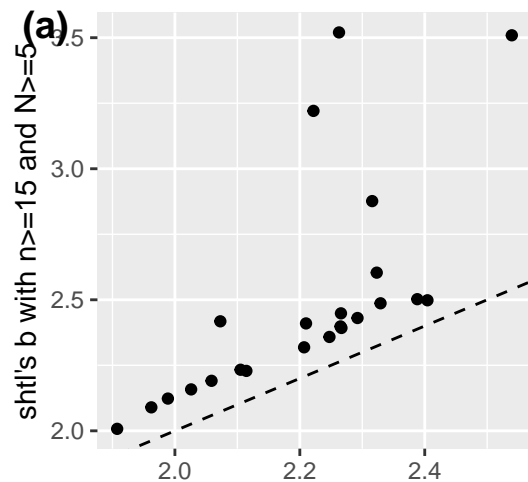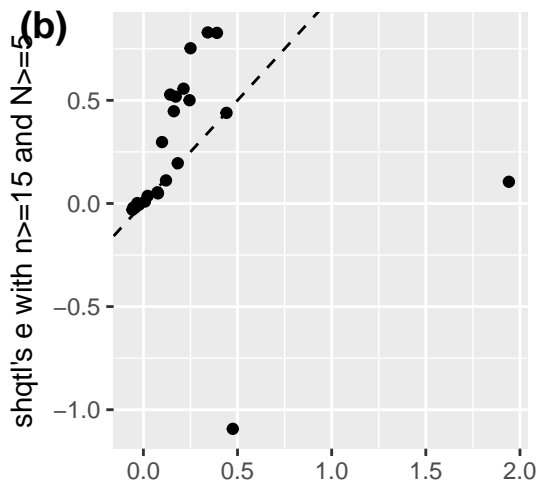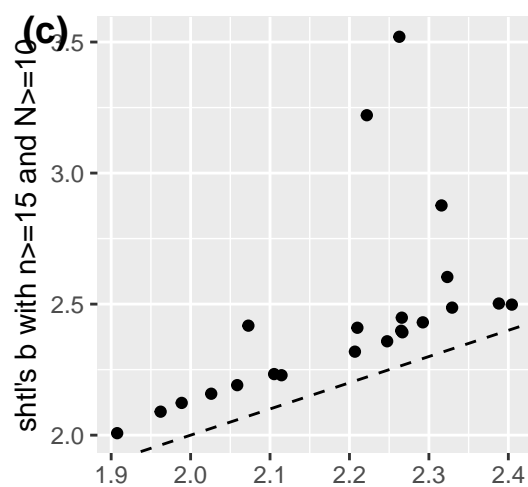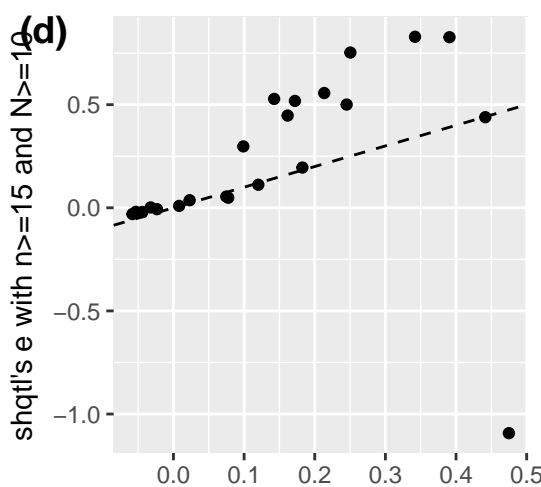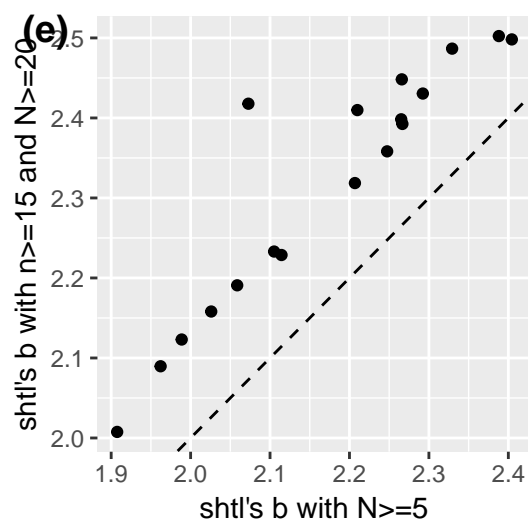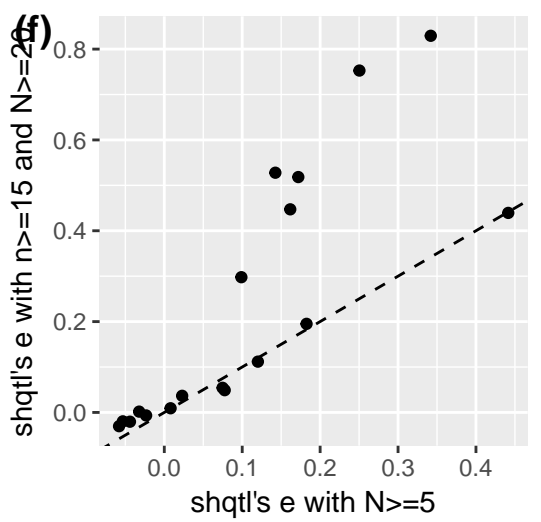

Supplement: S47 Fig — (PDF) [file pone.0226096.s048.pdf]

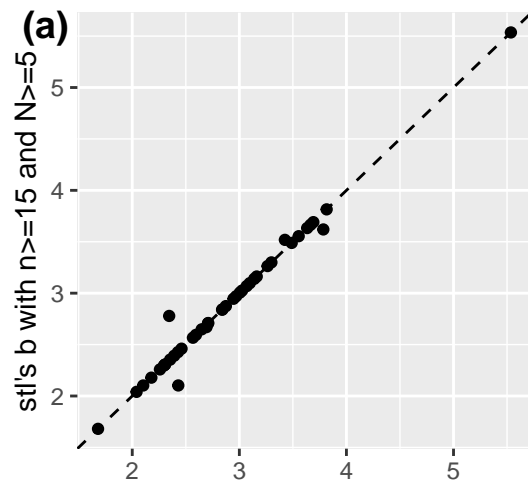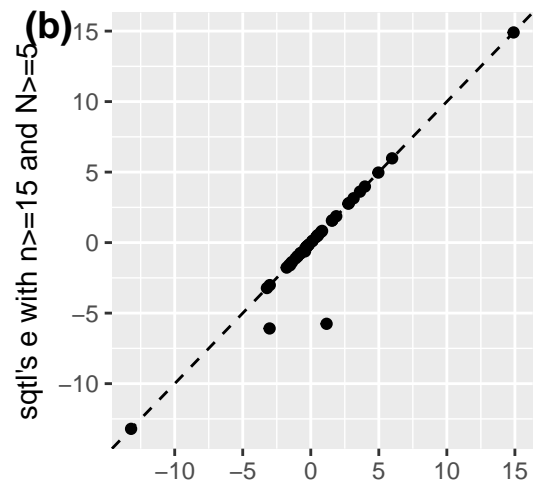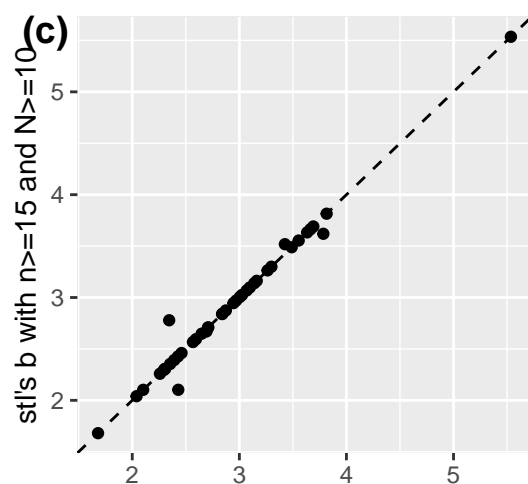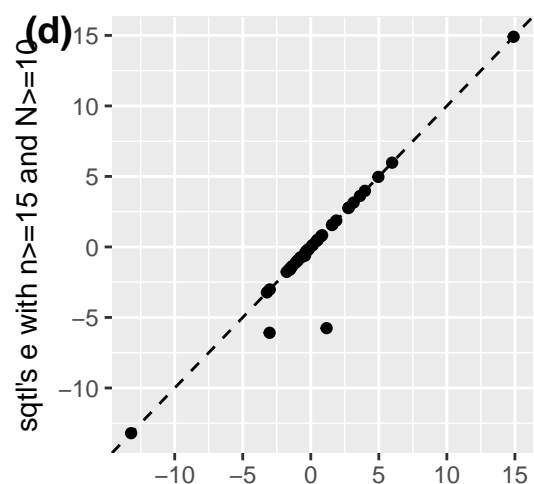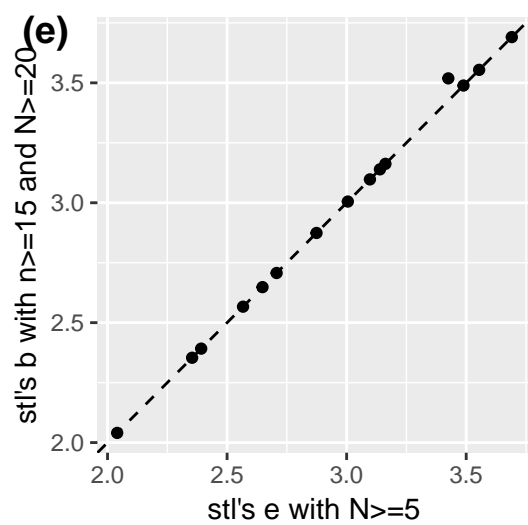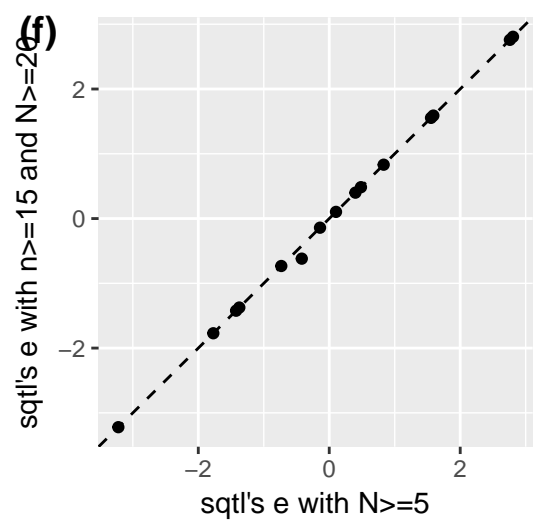

Supplement: S48 Fig — (PDF) [file pone.0226096.s049.pdf]

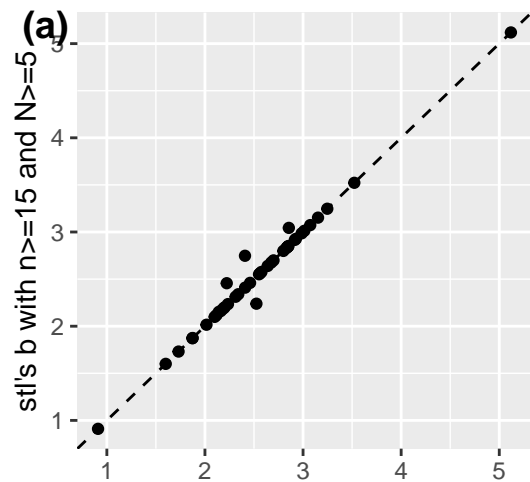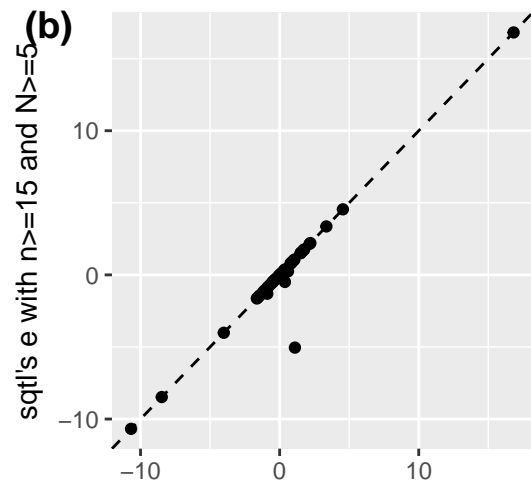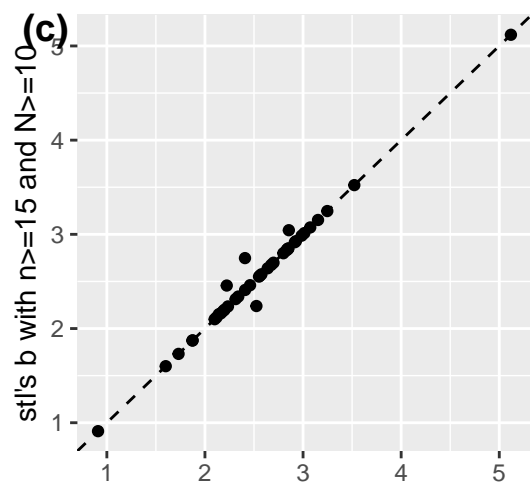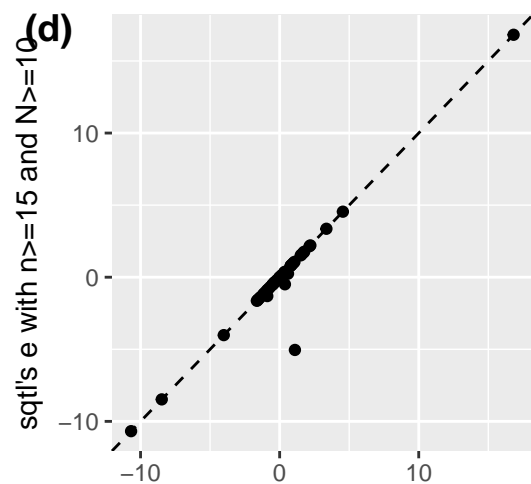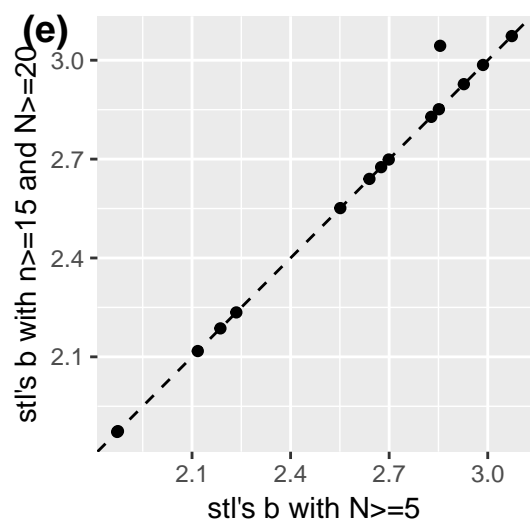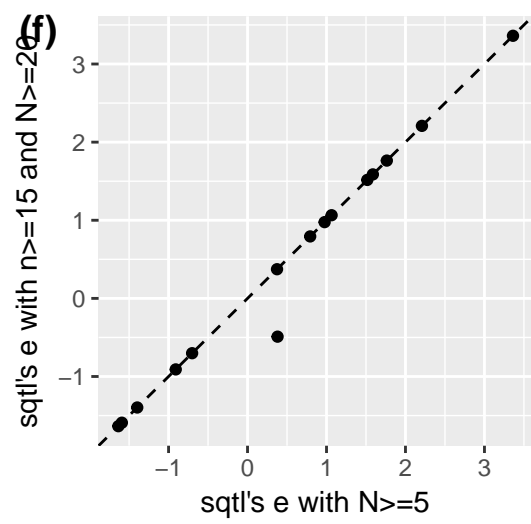

Supplement: S49 Fig — (PDF) [file pone.0226096.s050.pdf]

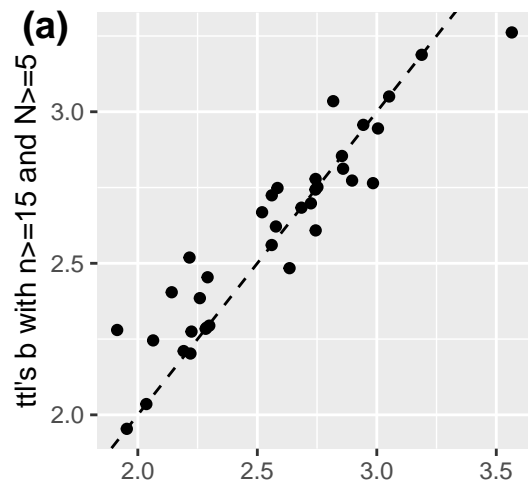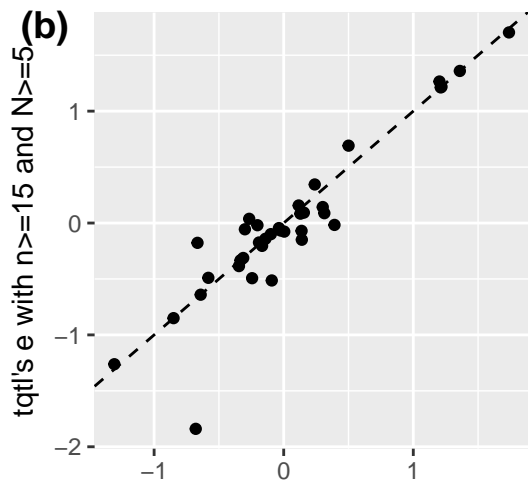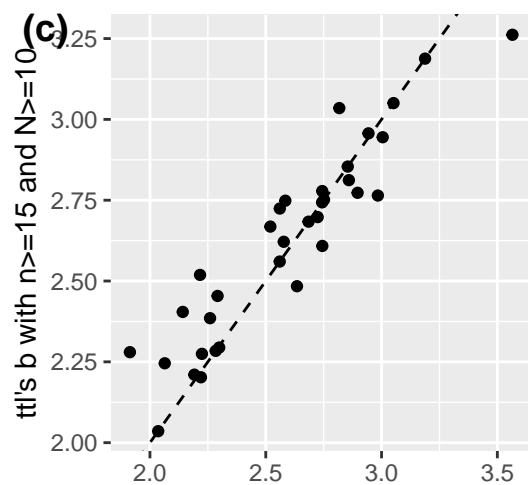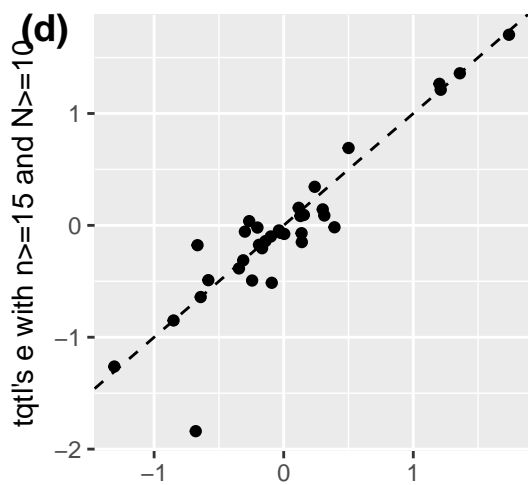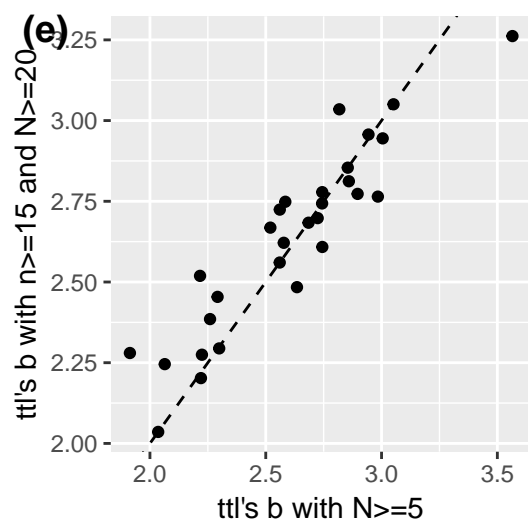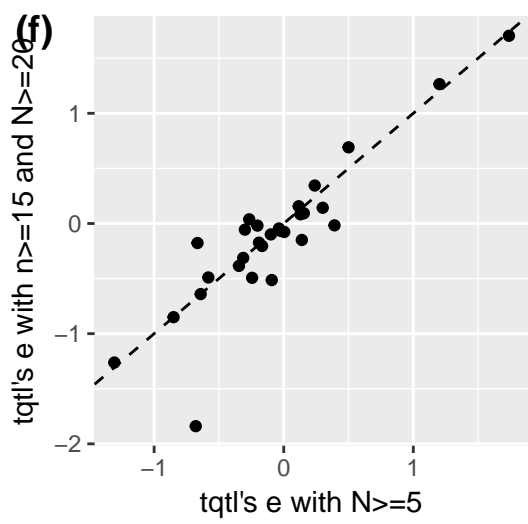

Supplement: S50 Fig — (PDF) [file pone.0226096.s051.pdf]

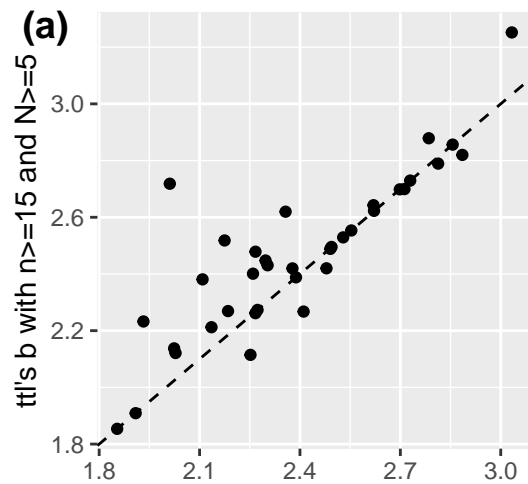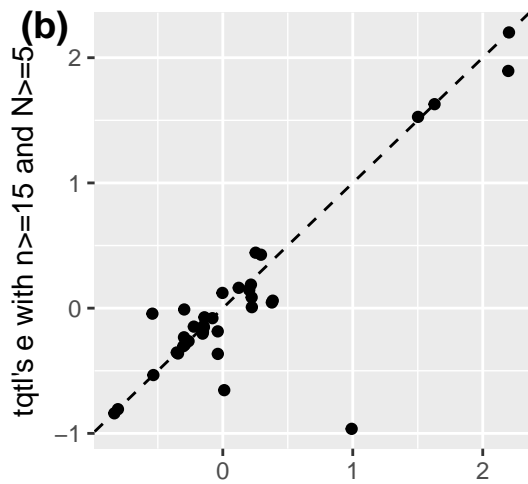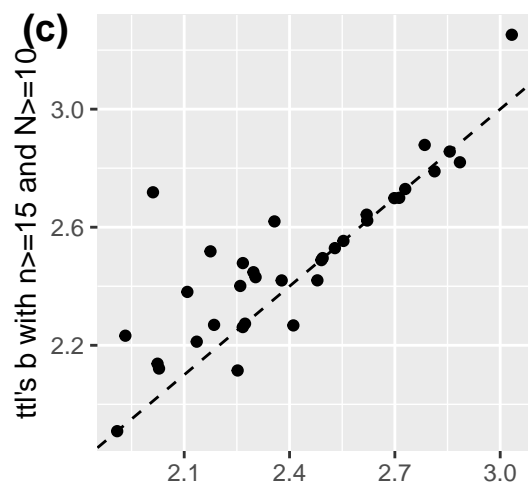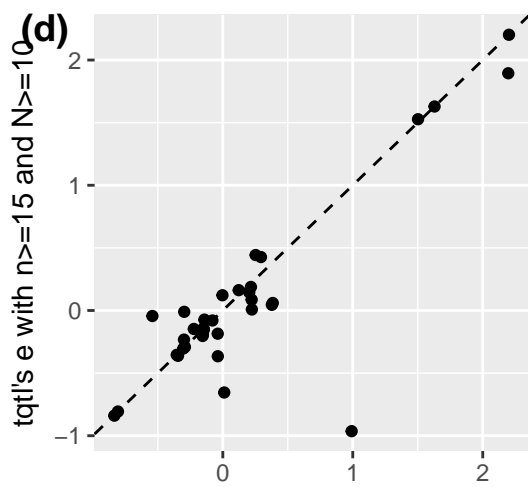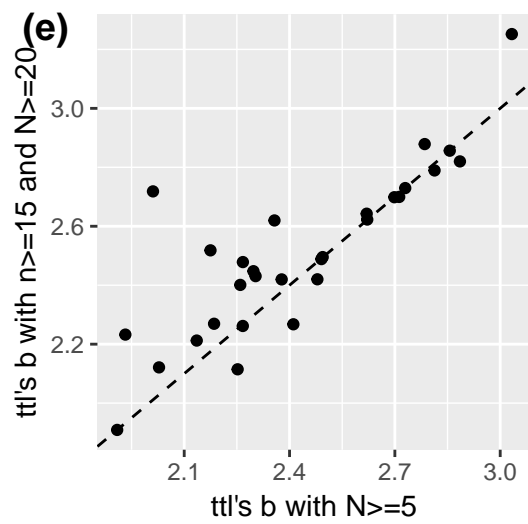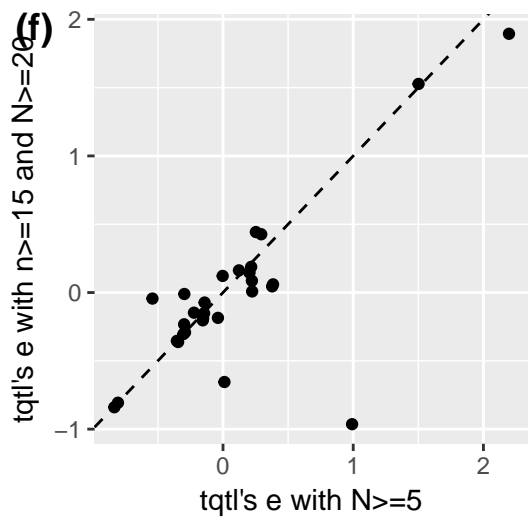

Supplement: S51 Fig — (PDF) [file pone.0226096.s052.pdf]
