# Supplementary material for: Analyzing and interpreting spatial and temporal variability of the United States county population distributions using Taylor's law
Source: PLoS One. 2019 Dec 11;14(12):e0226096. doi: 10.1371/journal.pone.0226096 (PMC6905577; doi:10.1371/journal.pone.0226096)
Supplement: S42 Fig — Point estimates of the slope ((a) and (b)) of ols linear regressions and the sign of the quadratic coefficient ((c) and (d)) of ols quadratic regressions for spatial TL for each state that occurs in at least 5 censuses, using count ((a) and (c)) and density ((b) and (d)). (PDF) [file pone.0226096.s043.pdf]

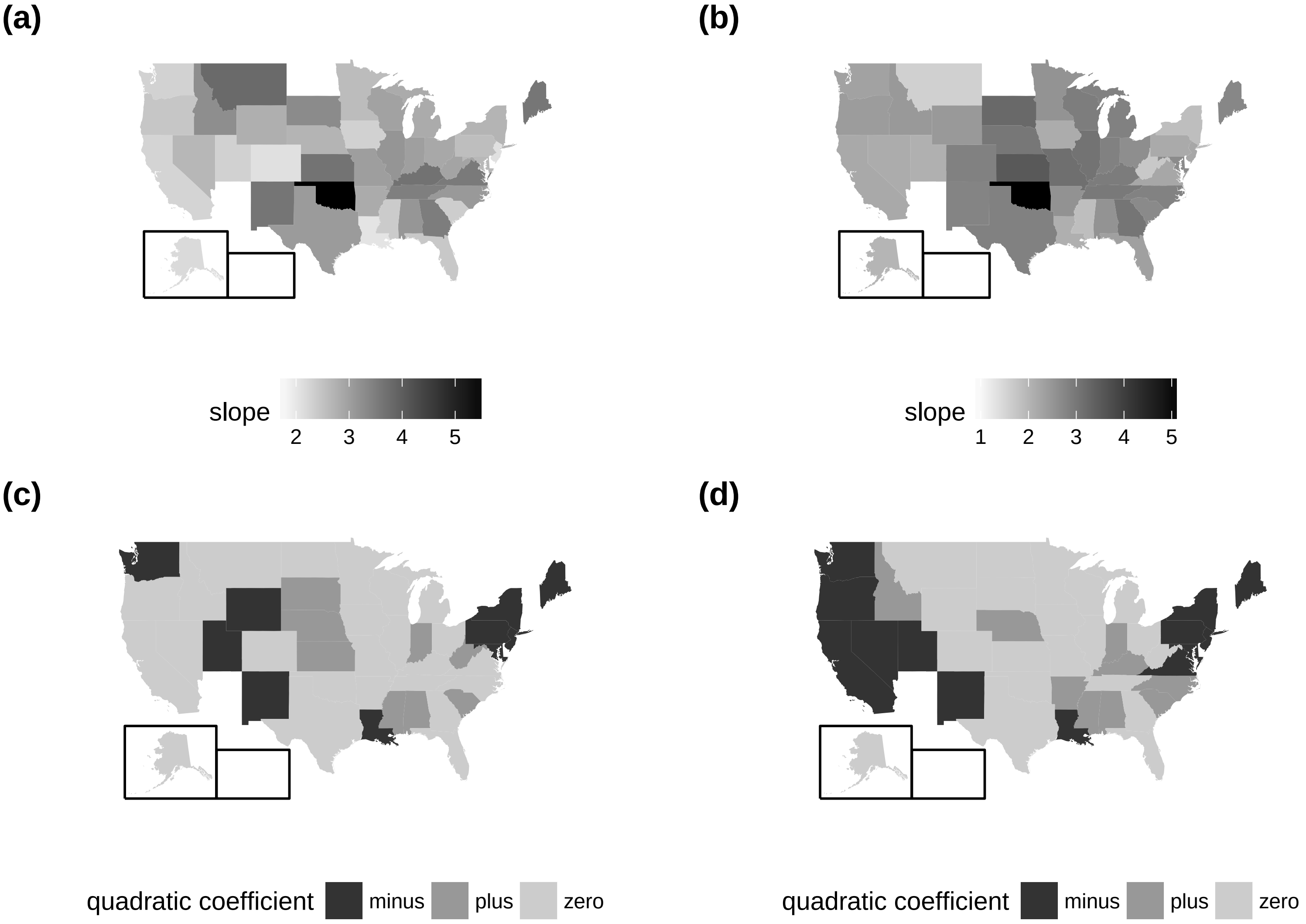
(a)
slope
2
3
4
5
(b)
slope
1
2
3
4
5
(c)
quadratic coefficient
minus
plus
zero
(d)
quadratic coefficient
minus
plus
zero
